# Quinone-heme π-π interactions bridge the biotic-abiotic interface for enhanced methanogenesis

**DOI:** 10.1038/s41467-026-77276-8

**Published:** 2026-09-01

**Authors:** Zhihao Jiang, Zhiqiang Zhao, Qilin Yu, Wenlong Lin, Yaobin Zhang

**Affiliations:** 1 Key Laboratory of Industrial Ecology and Environmental Engineering (Dalian University of Technology), Ministry of Education, School of Environmental Science and Technology, Dalian University of Technology, Dalian, China; 2 Department of Water Supply and Drainage, Taiyuan University of Technology, Taiyuan, China

**Keywords:** Biocatalysis, Electrocatalysis

## Abstract

Quinone-based conductive materials promote extracellular electron transfer and methanogenesis during anaerobic digestion, in which the materials mediates the transfer of electrons produced by exoelectrogens to electrotrophic methanogens. However, the electron transfer mechanism between quinone and electrotrophic methanogens at the biotic-abiotic interface remains elusive. In this study, an interface between reduced anthraquinone and *Methanosarcina barkeri* is established in a microbial electrolysis cell. Biomimetic experiments and characterization of isolated extracellular polymeric substances reveal electronic complementarity between the electron-donating π-system of reduced anthraquinone and the electron-accepting iron-porphyrin ring of heme, which may be released by cell lysis and retained within extracellular polymeric substances. This complementarity may promote π-π interactions that retain redox-active heme and facilitate electron transfer, consistent with lower interfacial resistance (34.3 versus 225 Ω) and 10.6-fold enhancement in cathodic methanogenesis. This study provides insight into quinone-mediated electron transfer at the biotic-abiotic interface and supports the design of electrode materials for bioelectrochemical systems.

## Abstract (web summary)

Quinone-based conductive materials promote extracellular electron transfer and methanogenesis during anaerobic digestion, in which the material mediates the transfer of electrons produced from exoelectrogens to electrotrophic methanogens, but the underlying mechanism remains elusive. Here, the authors establish the electron transfer interface between reduced anthraquinone (H₂AQC) and *Methanosarcina barkeri* in a microbial electrolysis cell, and reveal an intrinsic electronic complementarity between the electron donating π-system of H₂AQC and the electron-accepting iron-porphyrin ring of labile heme b.

## Introduction

Anaerobic digestion is an important approach for recovering resources and energy from organic waste. During conventional anaerobic digestion, syntrophic oxidation of fermentation products depends on methanogenic consumption of H₂ to maintain thermodynamically favorable conditions^1,2^. Alternatively, extracellular electron transfer can provide an efficient pathway to circumvent this thermodynamic barrier through direct or indirect electron connection between electrotrophic methanogens and exoelectrogens^3,4^. Conductive materials have been widely reported to enhance methane production by mediating the long-range extracellular electron transfer^5,6^. This process can be divided into two steps: (i) electron transfer within the conductive material, the efficiency of which depends primarily on the conductivity of the material (usually rapid); and (ii) electron transfer from the material surface to the methanogens, representing the electron transfer at the biotic-abiotic interface.

Conductive materials modified with redox-active functional groups, such as quinones, facilitate electron transfer via the quinone/hydroquinone redox cycle^7^, thereby enhancing electron uptake by methanogens from the material (step ii)^8^. For instance, previous studies have reported a positive correlation between methanogenic efficiency and the quinone content of biochar during anaerobic digestion^9^. Similarly, modifying carbon felt electrode surfaces with quinones promotes cathodic electron uptake by methanogens^10,11^. Generally, the contribution of quinone groups is attributed to the enhancement of electron storage capacity of the material surface resulting from their pseudocapacitive properties^12,13^. It is noteworthy, however, that methanogens (e.g., *Methanosarcina barkeri*) are typically encapsulated by a layer of weakly electroactive extracellular polymeric substances (EPS) composed primarily of polysaccharides and non-conductive proteins, ranging from tens to hundreds of nanometers in thickness^14^. Therefore, the enhanced electron storage capacity of the material surface alone is insufficient to explain the efficient electron transfer from immobilized quinones to methanogens, given the presence of EPS. So far, the mechanism of biotic-abiotic interface electron transfer by which immobilized quinones bridge the barrier of poorly conductive EPS to deliver electrons to methanogens remains elusive^8,15^.

To elucidate the electron transfer mechanism at the biotic-abiotic interface, it is necessary to identify the potential electroactive components within the EPS that accept electrons from quinone groups. Although the EPS layer of methanogens generally exhibits low electroactivity, it harbors a variety of redox mediators derived from extracellular secretions and cell lysis residues^16,17^. These potential electroactive components primarily include redox-active metalloproteins (e.g., cytochromes and iron-sulfur cluster proteins) and free electron-carrying coenzymes (e.g., coenzyme F420)^18^. However, most of these mediators struggle to maintain electroactivity within the EPS matrix. For instance, iron-sulfur (Fe-S) clusters rely on cysteine coordination within their protein scaffolds and are prone to inactivation following removal from their native protein environment or proteolytic disruption of the scaffold^19^. Similarly, the highly water-soluble coenzyme F420 tends to diffuse into the bulk solution and leach out, making it difficult to retain within the EPS^20,21^. In contrast, heme prosthetic groups derived from cytochromes, owing to their rigid porphyrin ring structure, may retain their structural stability for extended periods even after the degradation of the protein scaffold^22^. Furthermore, their hydrophobicity retards their diffusion into the aqueous phase. Therefore, these residual heme moieties within the EPS layer may serve as the bridge connecting quinones to methanogens.

The essence of the quinone-heme interaction lies in the electron transfer between a quinone molecule (reduced quinone) and the active center of heme (iron porphyrin ring). Structurally, both the reduced quinone and the iron porphyrin ring are planar aromatic molecules possessing extensive π-systems^23^. Electronically, they exhibit the complementarity: the hydroxyl (-OH) groups on the reduced quinone exert an electron-donating effect, rendering its π-system an electron donor, while the central iron ion exerts an electron-withdrawing effect, allowing the π-system to act as an electron acceptor^24–26^. Based on this, we hypothesize that quinone moieties specifically capture and immobilize labile heme prosthetic groups released into the EPS via π-π interactions. This interaction may stabilize heme, maintain its redox activity and limit its diffusion, thereby increasing the electroactivity of the EPS. Moreover, this inherent donor-acceptor electrostatic attraction may promote the association between of two molecules and enhance the overlap of their π-electron clouds, thereby promoting interfacial electron transfer^27,28^. This proposed mechanism provides a possible explanation for the observed quinone-mediated enhancement of electron transfer at the biotic-abiotic interface.

In this study, a biotic-abiotic interface between reduced quinone and *M. barkeri* was constructed to investigate the interaction Evidence consistent with an association between the π-systems of reduced quinone and heme in the EPS was obtained using a combination of scanning electrochemical microscopy (SECM), X-ray photoelectron spectroscopy (XPS), ultraviolet-visible (UV-Vis) spectroscopy, Raman spectroscopy, Fourier transform infrared (FTIR) spectroscopy, and density functional theory (DFT) calculations. These findings provide a perspective for understanding and regulating complex biotic-abiotic interfacial electron transfer processes.

## Results

### Construction of the M. b-H_2_AQC@CF interface

**Fig. 1:**
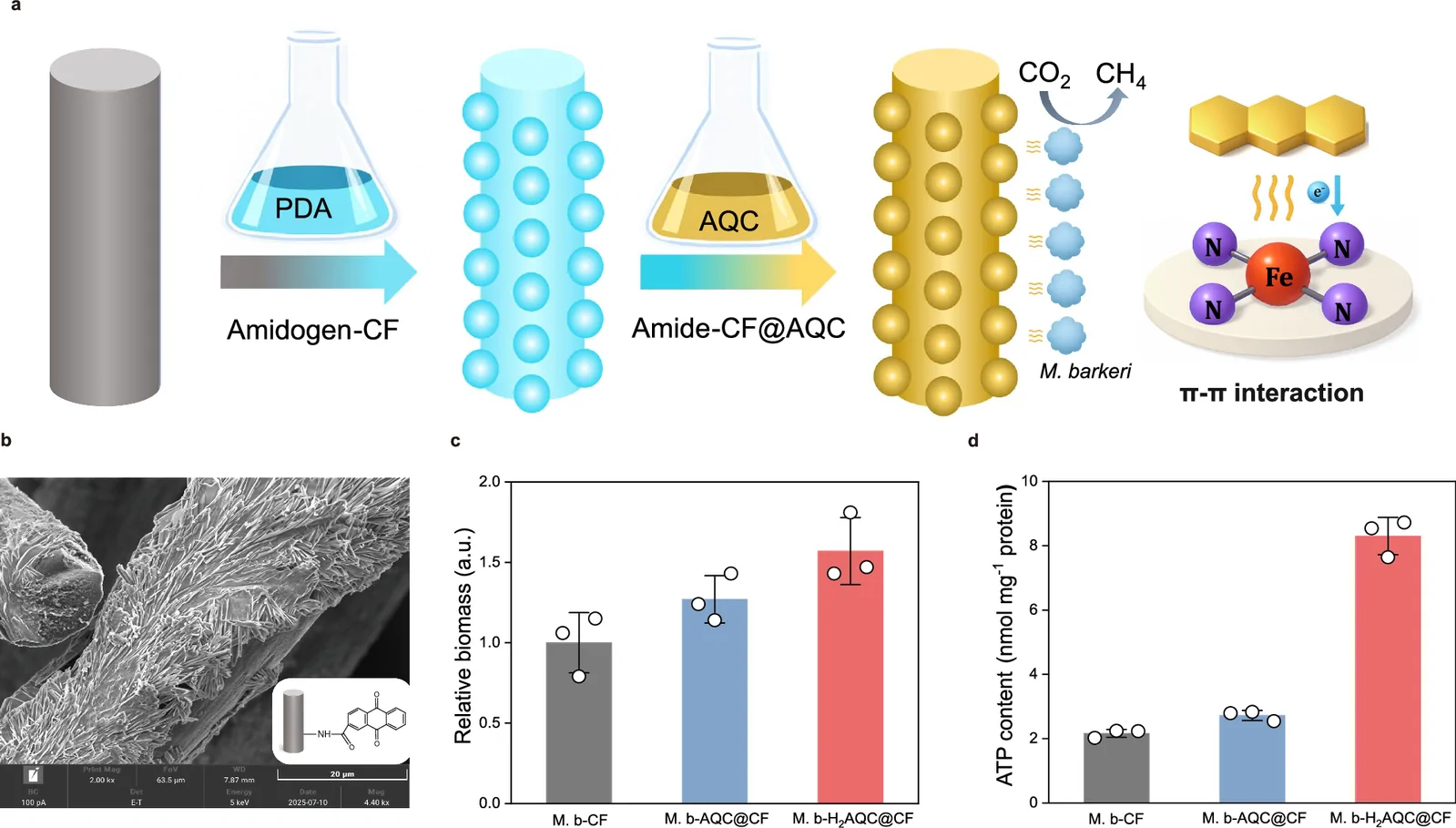
Fabrication and characterization of the AQC-modified biotic-abiotic interface. Schematic illustration of AQC@CF electrode synthesis (**a**). Scanning electron microscopy image of the AQC@CF electrode (**b**). Relative biomass of *M. barkeri* (**c**). Adenosine triphosphate (ATP) content of *M. barkeri* (**d**). AQC anthraquinone-2-carboxylic acid, H₂AQC reduced AQC, CF carbon felt, M. b *M. barkeri*. Data in (**c**) and (**d**) are presented as mean ± SD; circles represent individual biological replicates (*n* = 3 independently prepared biohybrid cathodes per group). The SEM imaging shown in (**b**) was independently repeated three times with similar results. Source data are provided as a Source Data file.

To construct a redox-active electrode interface, anthraquinone-2-carboxylic acid (AQC) was immobilized on carbon felt (CF), yielding the AQC@CF electrode (Fig. 1a). The fabrication consisted of a two-step process: initially, amine groups (-NH₂) were grafted onto the CF surface via the self-polymerization of dopamine. Subsequently, AQC was covalently attached to the aminated surface using N-hydroxysuccinimide (NHS)/1-ethyl-3-(3-dimethylaminopropyl)carbodiimide (EDC) coupling chemistry, forming stable amide bonds (O=C-NH) between its carboxyl group and the surface amine groups^29^ (Fig. 1b). The successful formation of an amide linkage was confirmed by Fourier transform infrared spectroscopy (FTIR) analysis^30^ (Supplementary Fig. 1). The electrochemical stability of the AQC@CF electrode was evaluated by cyclic voltammetry (CV) (Supplementary Fig. 2). The CV scans showed a characteristic reduction peak at approximately −0.59 V (vs. Ag/AgCl) and an oxidation peak at approximately −0.45 V (vs. Ag/AgCl). Neither the peak potentials nor the current densities showed appreciable decreases or shifts after multiple cycles, indicating the stability of the immobilized AQC.

Three groups of *M. barkeri* biohybrids with different biotic-abiotic interfaces were created by gradually attaching suspended *M. barkeri* to the cathode surfaces of H-type microbial electrolysis cells during a 30-day static cultivation. The performance of these three distinct biotic-abiotic interfaces for the electrocatalytic reduction of carbon dioxide to methane was subsequently tested. In the experimental M. b-H₂AQC@CF group, a constant potential of −0.8 V versus Ag/AgCl was applied to continuously reduce surface-bound AQC to H₂AQC. In the M. b-AQC@CF control, the electrode was maintained under open-circuit conditions. In the M. b-CF control, a constant potential of −0.8 V versus Ag/AgCl was applied to bare carbon felt.

Scanning electron microscopy (SEM) imaging confirmed the formation of *M. barkeri* biofilms across all three cathode surfaces (Supplementary Fig. 3). The abundance of the *mcrA* gene of the M. b-H₂AQC@CF biohybrid, which encodes the alpha subunit of methyl-coenzyme M reductase, was 1.57 and 1.24 times that in the M. b-CF and M. b-AQC@CF groups, respectively (Fig. 1c). Notably, the adenosine triphosphate (ATP) content of the M. b-H₂AQC@CF biohybrid exhibited an even greater increase, reaching 3.71 and 3.03 times higher than those of M. b-CF and M. b-AQC@CF, respectively (Fig. 1d). *M. barkeri* exhibited higher ATP content at the H₂AQC@CF interface, suggesting that the reduced quinones could bridge the poorly electroactive EPS barrier, thereby accelerating electron flux from the cathode to the cells.

### CO₂-to-methane conversion by M. b-H₂AQC@CF

The performance of the three cultured biohybrids in converting CO₂ to methane was evaluated in a microbial electrosynthesis system. These experiments were conducted at applied potentials ranging from −0.8 to −1.1 V versus Ag/AgCl. Under these donor-free test conditions, cathodic electrons served as the intended external source of reducing equivalents for CO₂ reduction by *M. barkeri*.

**Fig. 2:**
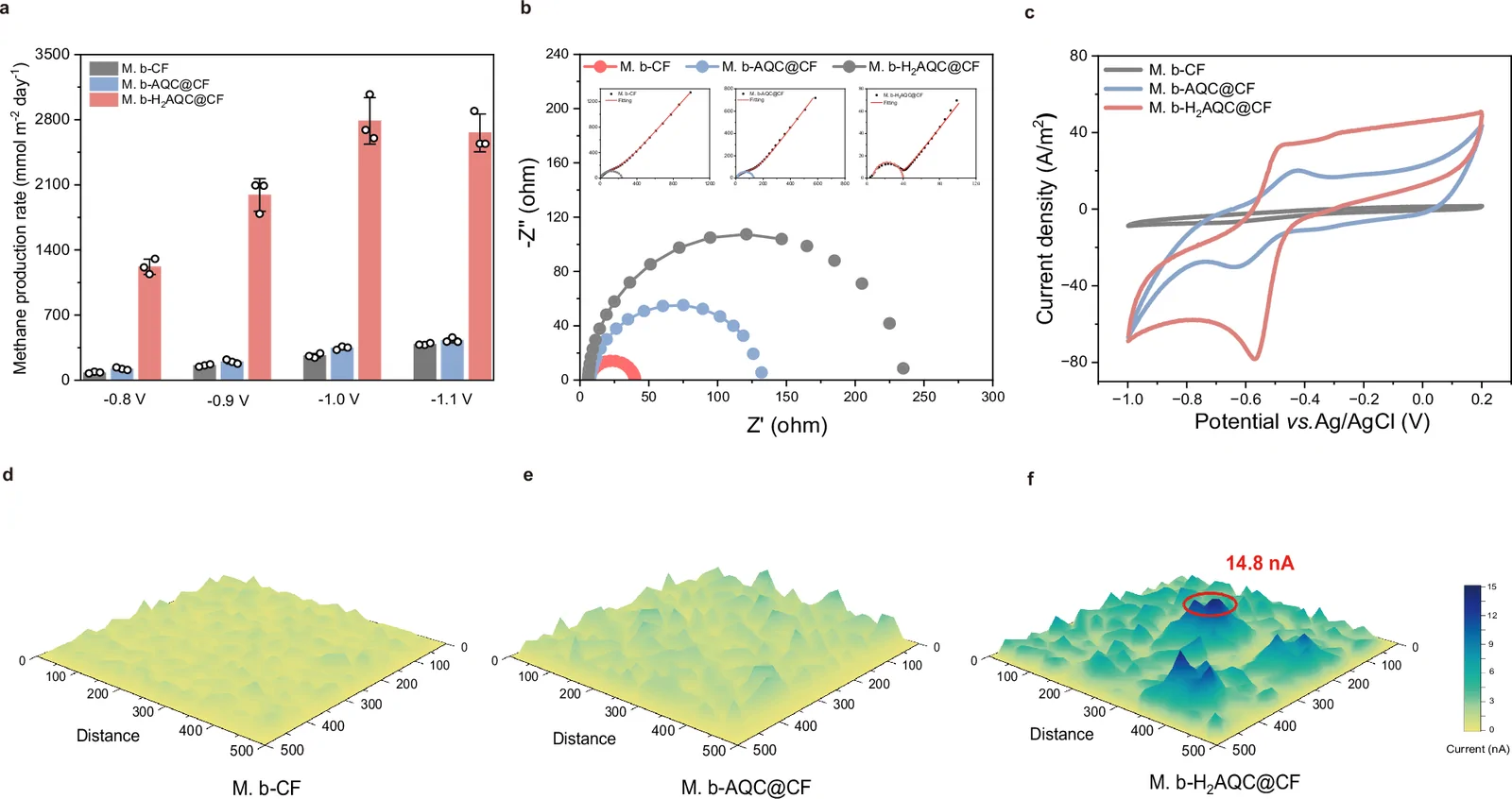
Performance and electrochemical characterization of the three biohybrids for CO₂-to-methane conversion. Methane production rate (**a**), electrochemical impedance spectroscopy (EIS) Nyquist plots (**b**), cyclic voltammetry (CV) curves (**c**), and three-dimensional scanning electrochemical microscopy (SECM) current-response maps (**d**–**f**). AQC anthraquinone-2-carboxylic acid, H₂AQC reduced AQC, CF carbon felt, M. b *M. barkeri*. In (**a**), data are presented as mean ± SD; circles represent individual biological replicates (*n* = 3 independently prepared biohybrid cathodes per group). Source data are provided as a Source Data file.

As shown in Fig. 2a, the M. b-H₂AQC@CF group exhibited a higher methane production rate than either control group at all tested potentials. The performance difference was most pronounced at an optimal potential of −1.0 V, where the M. b-H₂AQC@CF group achieved a peak production rate of 2787 mmol m⁻² day⁻¹. The methane production rate was 8.1 and 10.6 times those of the M. b-AQC@CF (346 mmol m⁻² day⁻¹) and M. b-CF (263 mmol m⁻² day⁻¹) control groups, respectively. A comparison with previously reported bioelectrochemical methane production systems is provided in Supplementary Fig. 4 and Supplementary Table S2. Consistently, the M. b-H₂AQC@CF group generated a peak current density of 29.02 A m⁻² (Supplementary Fig. 5), which was higher than those of the M. b-AQC@CF (3.95 A m⁻²) and M. b-CF (3.03 A m⁻²) groups. This current density corresponded to a maximum faradaic efficiency of 85.8% (Supplementary Fig. 5).

To investigate interfacial electron transfer in the three biohybrids, a series of electrochemical analyses was conducted. Fitting the electrochemical impedance spectroscopy data to a Randles equivalent circuit yielded a charge-transfer resistance (R_ct_) of 34.3 Ω for the M. b-H₂AQC@CF biohybrid, compared with 126.7 Ω for M. b-AQC@CF and 225.0 Ω for M. b-CF (Fig. 2b). These results are consistent with more efficient interfacial electron transfer in the presence of reduced AQC.

CV analysis revealed distinct differences in the electron transfer mechanisms among the three biohybrid systems (Fig. 2c). The M. b-AQC@CF biohybrid (blue line) exhibited a pair of reversible redox peaks consistent with the AQC molecule (Supplementary Fig. 2): a reduction peak at −0.59 V (reduction of AQC to H₂AQC) and an oxidation peak at −0.45 V (oxidation of H₂AQC to AQC). The reversibility suggests that during the potential sweep, the majority of H₂AQC generated was re-oxidized at the electrode. In contrast, the CV curve of the M. b-H₂AQC@CF biohybrid displayed a characteristic irreversible electrocatalytic profile: the reduction peak current at −0.59 V was enhanced, while the corresponding oxidation peak at −0.45 V was nearly abolished. This irreversibility, characterized by the absence of a re-oxidation peak, is consistent with increased electrochemical turnover of surface-bound H₂AQC coupled to interfacial electron transfer. As a reference, no discernible redox peaks were observed for the M. b-CF biohybrid. These findings indicate that the two control interfaces have greater kinetic limitations, resulting in less efficient electron transfer from the cathode to *M. barkeri*.

Furthermore, scanning electrochemical microscopy (SECM) was employed to visualize regions of enhanced electrochemical activity at the biotic-abiotic interfaces on the micrometer scale^31^. Specifically, a potential of −0.8 V (vs. Ag/AgCl) was applied to drive AQC reduction. SECM mapping of the M. b-H₂AQC@CF biohybrid revealed localized high-current regions (Fig. 2d–f). The peak current in these regions reached 14.8 nA, substantially higher than the corresponding peak currents in the M. b-AQC@CF (3.8 nA) and M. b-CF (2.1 nA) biohybrids, suggesting the presence of localized regions with enhanced electrochemical activity at the biotic-abiotic interface in the M. b-H₂AQC@CF biohybrid. Consistent with the EIS results, the average SECM current of the M. b-H₂AQC@CF biohybrid was markedly higher than that in the two control groups (Supplementary Fig. 6). These results support increased interfacial electron transfer in the presence of reduced AQC.

According to Marcus electron transfer theory, the electron transfer rate depends on the degree of electronic coupling (i.e., electron cloud overlap) between the electron donor and acceptor^32,33^. The dimensions of these electrochemically active regions were comparable to those of *M. barkeri* cells. This spatial correspondence supports close spatial coupling between H₂AQC and electroactive sites associated with the cell interface, although SECM alone does not identify the molecular components responsible for electron transfer. Together with the CV and EIS results, these observations support enhanced interfacial electron transfer in the M. b-H₂AQC@CF biohybrid.

### Extracellular heme as an electron-transfer site

**Fig. 3:**
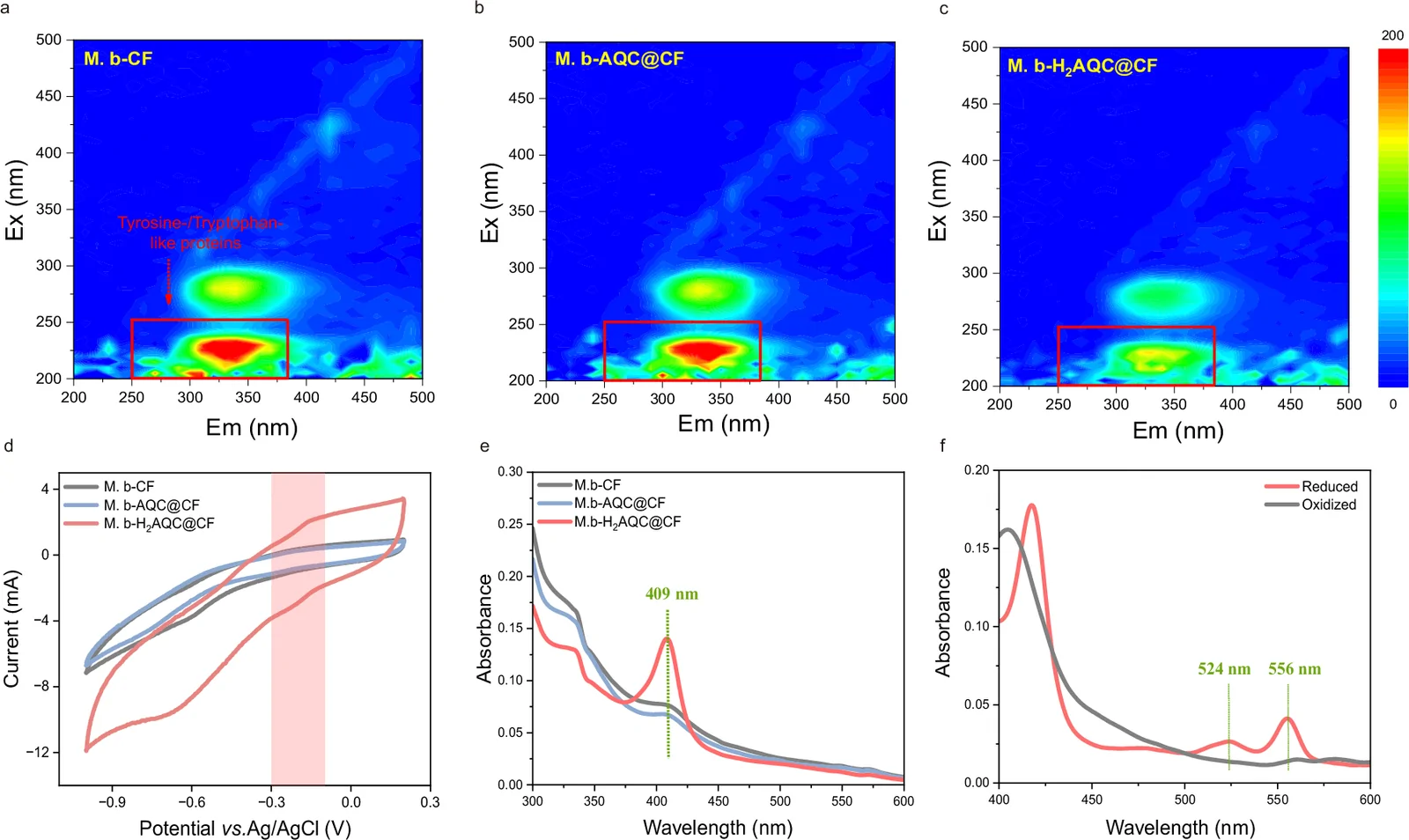
Spectroscopic and electrochemical characterization of extracellular polymeric substances from the three biohybrid groups. Three-dimensional excitation-emission matrix (3D-EEM) fluorescence spectra of extracellular polymeric substances (EPS) from the three biohybrid groups (**a**–**c**), cyclic voltammograms of the EPS (**d**), ultraviolet-visible (UV–Vis) absorption spectra of the EPS (**e**), and UV–Vis spectra of EPS from the M. b-H₂AQC@CF group in the oxidized and chemically reduced states (**f**). AQC anthraquinone-2-carboxylic acid, H₂AQC reduced AQC, CF carbon felt, M. b *M. barkeri*. Source data are provided as a Source Data file.

Because EPS form part of the interface between *M. barkeri* and the H₂AQC@CF cathode, EPS fractions from the three biohybrids were analysed^16^. The relative protein content of the M. b-H₂AQC@CF EPS fraction did not differ markedly from that in the two control groups (Supplementary Fig. 7). However, the results of three-dimensional excitation-emission matrix (3D-EEM) fluorescence spectroscopy revealed an different  trend: the characteristic fluorescence intensity of aromatic protein-like substances (tryptophan and tyrosine) in the EPS of M. b-H₂AQC@CF biohybrid was lower than that of the two control groups^34^ (2.65 a.u. in the M. b-H₂AQC@CF group, compared with 4.03 and 4.19 a.u. in the M. b-AQC@CF and M. b-CF groups, respectively, Fig. 3a–c, Supplementary Fig. 8). CV analysis further revealed that the EPS from the M. b-H₂AQC@CF group exhibited a pair of clear and reversible redox peaks from −0.15 V to −0.25 V (Fig. 3d), closely matching the characteristic redox potential of electroactive heme cofactors reported in the literature^35,36^. Notably, heme is a large iron-porphyrin π-conjugated system whose absorption spectrum overlaps with the emission spectra of aromatic amino acids, allowing it to quench fluorescence via electron transfer^37,38^. This property suggested the possible presence of heme within the EPS of the M. b-H₂AQC@CF biohybrid, which could account for the aforementioned observation of comparable protein content but low fluorescence intensity.

To assess this possibility, several complementary measurements were performed. As shown in Fig. 3e, ultraviolet-visible (UV-Vis) spectroscopy revealed a sharp Soret band at 409 nm exclusively in the EPS of the M. b-H₂AQC@CF group, consistent with monomeric ferric (Fe³⁺) heme, while the control groups exhibited only weak and broad absorption in this region, possibly attributable to trace levels of heme b below the threshold for reliable spectroscopic resolution. Upon treatment with pyridine and chemical reduction (pyridine hemochrome assay), characteristic α/β bands appeared at 556 nm and 524 nm (Fig. 3f), representing the classical pyridine hemochrome signatures of Fe²⁺ heme^39,40^. To distinguish heme b from heme c, bovine heart cytochrome c (a heme c containing reference standard) was measured in parallel, yielding α/β bands at 550 and520 nm (Supplementary Fig. 9), consistent with established literature values. These results provide spectral evidence that the observed heme is heme b^41^. Together, these spectral features are consistent with the presence of redox-active heme b. Further corroborating evidence was provided by heme-specific lithium dodecyl sulfate-polyacrylamide gel electrophoresis (LDS-PAGE) staining, which revealed a positive signal in the low molecular weight region exclusively in the M. b-H₂AQC@CF group, consistent with labile heme released following cell lysis, while both control groups were negative (Supplementary Fig. 10)^42^. Together, these results indicate the presence of redox-active heme within the EPS of the M. b-H₂AQC@CF biohybrid.

The electron paramagnetic resonance (EPR) spectrum displayed a discernible peak at g = 3.04 (Supplementary Fig. 11), characteristic of a low-spin ferric (Fe³⁺) heme center^43^. Chemical reduction with sodium dithionite completely abolished this signal, supporting the assignment of this signal to a reducible Fe³⁺ heme center. Notably, free ferric heme b typically exhibits a high-spin EPR signal near g ≈ 6. The absence of this feature, together with the signal at g = 3.04, is consistent with the presence of a six-coordinate low-spin ferric heme species. Such a state could be stabilized by axial coordination to imidazole- or amine-containing components of the EPS or by partial retention of axial ligands from the parent hemoprotein^44^. Electrochemical infrared spectroscopy provided additional evidence that the extracellular heme-associated species retained electrochemical activity and participated in electron transfer (Supplementary Fig. 12). As the applied potential was scanned from +0.2 V to −0.8 V, the absorption intensities of both the amide II (~1550 cm⁻¹) and amide I (~1650 cm⁻¹) bands increased in the M. b-H₂AQC@CF biohybrid^31^, while the spectral responses of the two control groups were markedly weaker, indicating that the H₂AQC@CF interface facilitated electron transfer from the cathode to the heme active center. Together, these results suggest that labile heme may have been retained at the H₂AQC@CF interface and remained electrochemically active.

### Biomimetic experiments and DFT analysis of the H₂AQC-heme interaction

To elucidate the interaction between H₂AQC and heme at the molecular level, a biomimetic model system was developed: FeTCPP (iron porphyrin, mimicking the active site of heme) was electrodeposited onto the surface of an H₂AQC-loaded CF electrode, thereby constructing a directional electron transfer chain from CF to H₂AQC and to FeTCPP (Supplementary Fig. 13).

**Fig. 4:**
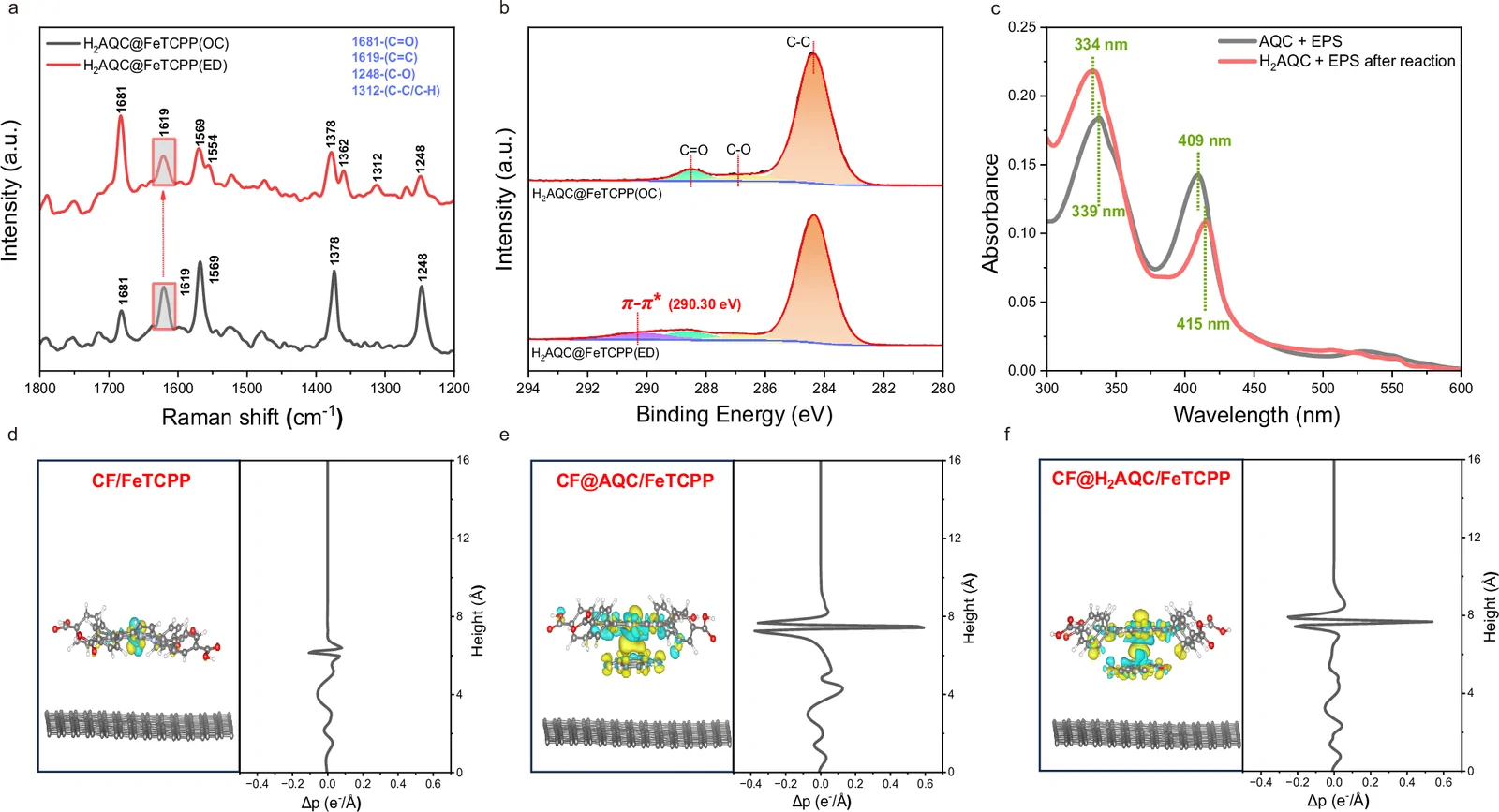
Spectroscopic and computational characterization of the H_2_AQC-FeTCPP interaction. Raman spectra of H_2_AQC@FeTCPP(OC) and H₂AQC@FeTCPP(ED) (**a**); C 1*s* X-ray photoelectron spectroscopy (XPS) spectra (**b**). H₂AQC@FeTCPP(OC) denotes the physically mixed control, whereas H₂AQC@FeTCPP(ED) denotes the electrodeposited sample. Ultraviolet-visible (UV-Vis) spectra of AQC + EPS and H₂AQC + EPS after reaction (**c**). Density functional theory (DFT) analysis and charge-density-difference (CDD) plots for CF/FeTCPP (**d**), CF@AQC/FeTCPP (**e**), and CF@H₂AQC/FeTCPP (**f**). In **d**–**f**, the left side displays the three-dimensional isosurface plot, and the right side presents the plane-averaged charge-density-difference curve along the z-axis. Yellow and cyan indicate electron accumulation and depletion, respectively (isosurface value = 0.002 atomic units (a.u.)). FeTCPP iron meso-tetrakis(4-carboxyphenyl)porphyrin, AQC anthraquinone-2-carboxylic acid, H₂AQC reduced AQC, CF carbon felt, EPS extracellular polymeric substances. Source data are provided as a Source Data file.

Raman spectroscopy was used to examine interactions within the biomimetic interface. In the physically mixed H₂AQC@FeTCPP(OC) sample, the bands at 1569 and 1378 cm⁻¹ were assigned to the ν₂ and ν₄ modes of Fe(III)TCPP, respectively^45^. In the electrodeposited H₂AQC@FeTCPP(ED) sample, these bands were attenuated, whereas bands assigned to Fe(II)TCPP at 1554 and 1362 cm⁻¹ increased^46^. These spectral changes are consistent with partial reduction of Fe(III)TCPP to Fe(II)TCPP and support electron transfer from H₂AQC to FeTCPP. Attenuation of the aromatic C=C stretching band at 1619 cm⁻¹ is also consistent with an interaction between their π-systems^47^, although it does not by itself establish a specific stacking geometry. In agreement, the C 1*s* spectra from XPS analysis revealed a satellite peak at 290.30 eV, attributed to π-π interactions, was observed for H₂AQC@FeTCPP(ED), whereas this peak was absent from H₂AQC@FeTCPP(OC) (Fig. 4b)^48^.

To validate the proposed quinone-heme interaction, UV-Vis spectroscopy were conducted. Pure AQC (oxidized) and H₂AQC (reduced) exhibit absorption peaks at 339 nm and 407 nm, respectively (Supplementary Fig. 14). Mixing AQC with EPS (M. b-H₂AQC@CF) maintained the 339 nm peak, indicating that the EPS matrix does not inherently perturb AQC absorption. In contrast, adding H₂AQC to the EPS fraction (M. b-H₂AQC@CF) produced spectral changes consistent with a redox reaction in which H₂AQC was oxidized to AQC by Fe³⁺ heme b, resulting in an AQC peak at 334 nm (Fig. 4c). This 5 nm blue shift is consistent with that H₂AQC may initially form a π-π complex with heme b through electronic overlap, which allows the resulting AQC to remain retained at the interaction site and preserve the π-π stacked configuration^49,50^.

Density functional theory (DFT) calculations were performed to evaluate the proposed association between reduced quinone (H₂AQC) and the heme model. When H₂AQC was in proximity to FeTCPP, the charge density difference plot showed marked interfacial charge redistribution (Fig. 4f). A clear electron change was observed from the H₂AQC region (electron depletion, cyan) to the FeTCPP region (electron accumulation, yellow). Quantitative charge analysis indicated a net charge transfer of 0.158 *e* (Supplementary Fig. 15). This phenomenon was attributed to the interaction between H₂AQC and FeTCPP, in which acted as the electron donor and electron acceptor, respectively. Specifically, the two electron-donating hydroxyl (-OH) groups within the H₂AQC created an electron-rich π-conjugated system^25,26^, thereby enabling it to function as an electron donor. Conversely, the electron-withdrawing effect of the iron center contributed to the electron-accepting character of  FeTCPP. This calculated donor-acceptor relationship supports an association between the π-systems of H₂AQC and the heme model that may contribute to electron transfer. In contrast, the π-system of AQC only functioned as an electron acceptor because of its two electron-withdrawing carbonyl (C=O) groups^26^. Therefore, the interaction between AQC and FeTCPP induced electron transfer in the opposite direction, from FeTCPP to AQC. In comparison, the calculated charge transfer between bare CF and FeTCPP was 0.022 *e* (Supplementary Fig. 15).

DFT calculations indicated electronic complementarity between reduced quinone as an electron donor and FeTCPP as an electron acceptor. The calculated charge redistribution and net charge transfer support a π-π association between the two molecules. This association may contribute to electron transfer and heme retention at the H₂AQC@CF interface.

### Transcriptomic analysis of electron transfer and methanogensis pathways

**Fig. 5:**
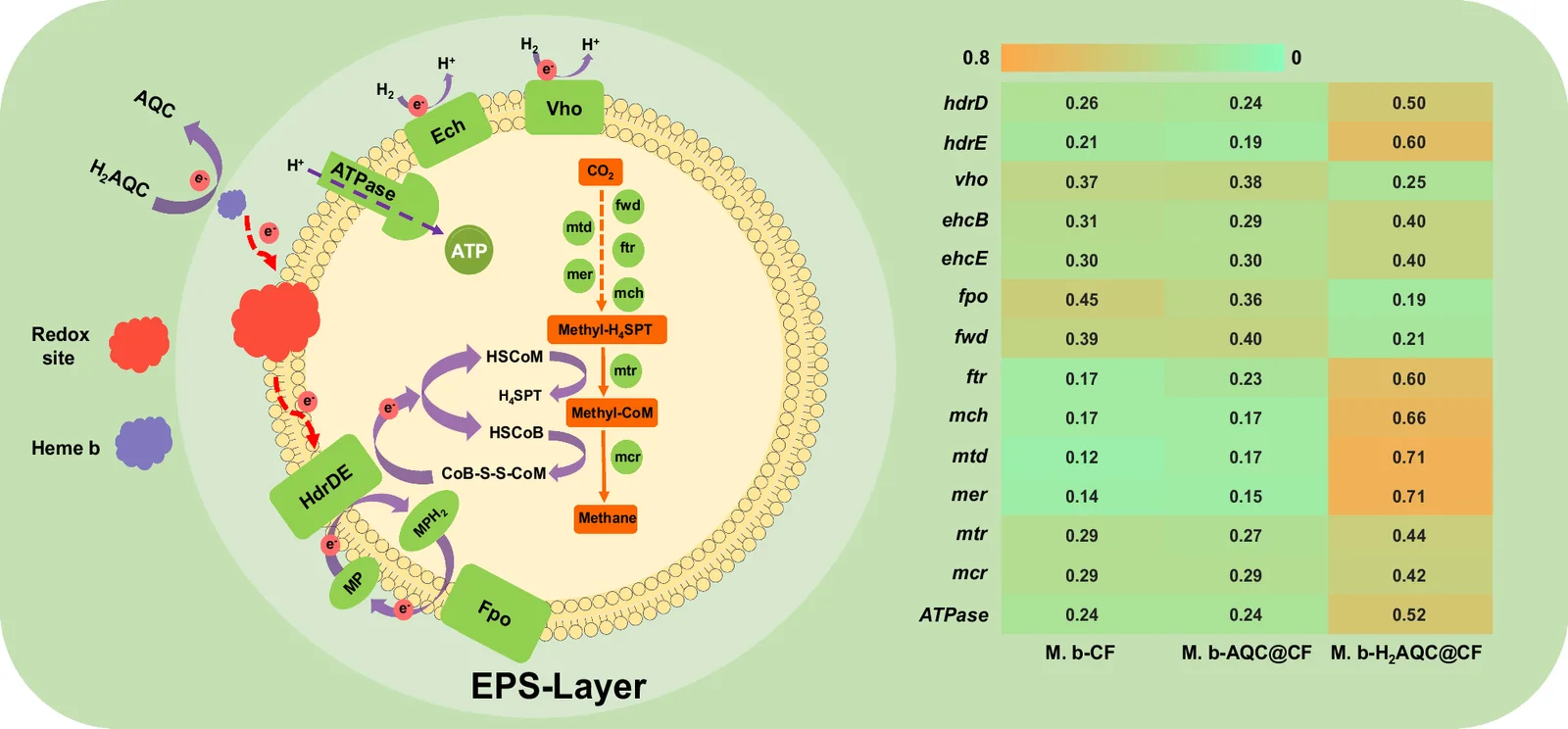
Relative transcript levels of genes encoding membrane electron transfer components and key enzymes in the methanogenic pathway in *M. barkeri*. The heatmap displays normalized reads per kilobase of transcript per million mapped reads (RPKM) values, scaled to a range of 0-0.8 within each gene row, for the three biohybrid groups. The schematic illustrates the proposed electron transfer pathway at the biotic-abiotic interface. Arrows indicate the proposed direction of electron transfer. EPS extracellular polymeric substances, AQC anthraquinone-2-carboxylic acid, H₂AQC reduced AQC, CF carbon felt, M. b M. barkeri. Gene symbols: *hdrD* and *hdrE* heterodisulfide reductase subunits D and E, *vho* F₄₂₀-non-reducing hydrogenase, *echB* and *echE* energy-converting hydrogenase subunits B and E, *fpo* F₄₂₀H₂ dehydrogenase, *fwd* formylmethanofuran dehydrogenase, *ftr* formylmethanofuran-tetrahydromethanopterin N-formyltransferase, *mch* methenyltetrahydromethanopterin cyclohydrolase, *mtd* methylenetetrahydromethanopterin dehydrogenase, *mer* methylenetetrahydromethanopterin reductase, *mtr* methyltransferase, *mcr* methyl-coenzyme M reductase. Source data are provided as a Source Data file.

Transcriptomic analysis, visualized via a heatmap, provided a comprehensive profile of the membrane electron transfer components *(hdrDE*, *vho*, *echBE*, *fpo*) and key enzymes in the methanogenic pathway (*fwd*, *ftr*, *mch*, *mtd*, *mer*, *mtr*, *mcr*)^51,52^. Among these genes, *hdrD* and *hdrE* showed higher relative transcript levels in the M. b-H₂AQC@CF biohybrid. Their normalized transcript abundance values were 0.50 and 0.60, respectively, compared with 0.24 and 0.19 in M. b-AQC@CF and 0.26 and 0.21 in M. b-CF (Fig. 5).

The higher expression of *hdrD* and *hdrE* may represent an adaptive response to the interfacial environment and may be associated with the interaction between H₂AQC and heme centers. Based on the established role of HdrDE in EET for *M. barkeri*, a potential electron transfer pathway is proposed (Fig. 5). Consistent with this enhanced electron influx, gene encoding key enzymes in the downstream methanogenic pathway also exhibited notably increased expression levels in the biohybrid system. Furthermore, HdrDE may contribute to the establishment of a transmembrane proton motive force during extracellular electron transfer, potentially supporting ATP synthesis^53,54^. Therefore, the higher transcript levels of *hdrD* and *hdrE* may account for the enhanced ATP synthesis and may be associated with the higher ATP content observed in Fig. 1d; the ATP content in the M. b-H₂AQC@CF group was 3.71 times that in the M. b-CF group. These results further supporte the establishment of an efficient electron transfer pathway involving π-π interactions (H₂AQC-heme) at the biotic-abiotic interface in the M. b-H₂AQC@CF biohybrid.

## Discussion

Cell turnover and lysis can release membrane-associated components into the EPS matrix^55,56^. Unlike covalently bound c-type cytochrome hemes, non-covalently bound heme b can readily dissociate from its protein scaffold upon cell disruption^57^, entering the EPS as labile heme. Consistent with this possibility, extracellular heme has previously been observed in *M. barkeri* cultures^58,59^. Notably, the HdrE subunit harbors two heme b cofactors^18^, representing one plausible source of labile heme released into the EPS; other heme b-containing membrane complexes may also contribute labile heme upon cell disruption. Cellular turnover may therefore provide a source of heme-containing material at the interface.

Labile heme released into aqueous environments is typically prone to self-aggregation, forming electrochemically inactive aggregates. The sharp Soret band at 409 nm and the reversible redox response observed for the M. b-H₂AQC@CF EPS fraction are consistent with the retention of electrochemically active, relatively non-aggregated heme-associated species. This may arise because the H₂AQC interface competitively inhibits heme auto-dimerization via preferential π-π interactions. Such stabilization mechanisms have been documented for porphyrin immobilization on aromatic carbon interfaces^60,61^. The biomimetic experiments and DFT calculations further support an association between the π-systems of H₂AQC and an iron-porphyrin model. Such an association may contribute to heme retention and facilitate electron transfer, but the precise molecular geometry and binding stoichiometry remain to be established.

The higher transcript levels of *hdrD* and *hdrE*, together with increased ATP content and methane production, support a cellular response to the altered interfacial redox environment. Nevertheless, these correlations do not establish that electrons are transferred exclusively or directly through HdrDE, and other membrane redox components and changes in biofilm physiology cannot be excluded. Overall, the results support a model in which reduced surface-bound quinones interact with redox-active heme-associated species in the EPS and thereby contribute to enhanced interfacial electron transfer and methanogenesis.

## Methods

### Preparation of the AQC@CF electrode

Carbon felt was sonicated sequentially in acetone, ethanol, and deionized water to remove surface contaminants, followed by drying at 60 °C. For surface amination, the felt was immersed in a 2 mg/mL dopamine solution (10 mM tris(hydroxymethyl)aminomethane hydrochloride, pH 8.5) for 24 h. After washing, the resulting polydopamine-coated carbon felt (CF-PDA) was dried at 60 °C for 24 h. Finally, the CF-PDA was reacted for 24 h in the dark with an activated solution containing AQC (0.05 mmol), EDC (0.075 mmol), and NHS (0.06 mmol) in 20 mL of anhydrous N,N-dimethylformamide (DMF). The resulting AQC@CF electrode was purified via sonication in DMF, ethanol, and deionized water to remove residual reactants on the surface of the electrode, and subsequently dried at 80 °C for 24 h.

The electrochemically active AQC loading was determined by integrating the background-corrected cathodic redox peak obtained at a scan rate of 50 mV s⁻¹. The surface loading was calculated using Γ = Q/(nFA), where Q is the integrated charge, *n* = 2, F is the Faraday constant (96,485 C mol⁻¹), and A is the projected electrode area (1 cm²). The apparent electrochemically active AQC loading was 1.952 ± 0.136 nmol cm⁻², corresponding to 0.492 ± 0.034 μg cm⁻² (mean ± SD, *n* = 3 independently prepared electrodes). H₂AQC@CF was prepared by reducing AQC@CF and therefore had the same initial AQC loading. The unmodified CF control contained no AQC.

### Preparation of biohybrids and electrochemical tests

*Methanosarcina barkeri* DSM 800 was obtained from the Leibniz Institute DSMZ-German Collection of Microorganisms and Cell Cultures GmbH (Braunschweig, Germany). The strain was cultivated under strictly anaerobic conditions in a modified medium based on DSMZ Medium 120. The detailed medium composition is provided in Supplementary Table 1. All electrochemical tests were performed in a dual-chamber H-type cell (100 mL per chamber) separated by a proton exchange membrane. A standard three-electrode system was employed, using the as-prepared biohybrid electrode as the working electrode (cathode), an Ag/AgCl (saturated KCl) electrode as the reference electrode, and a ruthenium-plated iridium oxide electrode as the counter electrode (anode). The biohybrid cathodes were prepared by inoculating *Methanosarcina barkeri* cell suspension into the cathode chambers of three groups, with each group conducted in triplicate. The biohybrid cathodes were then cultivated for 30 days under otherwise identical static conditions. The experimental M. b-H₂AQC@CF group was maintained at −0.8 V versus Ag/AgCl to continuously reduce surface-bound AQC to H₂AQC. The M. b-AQC@CF control was maintained under open-circuit conditions to retain surface-bound AQC predominantly in its oxidized state, whereas the M. b-CF control consisted of unmodified carbon felt maintained at −0.8 V versus Ag/AgCl. To prevent substrate depletion and maintain microbial activity during biofilm enrichment, 10 mL of fresh medium containing methanol was added to each cathode chamber on days 10 and 20. The complete composition of the cathode medium is provided in Supplementary Table 1.

Prior to performance evaluation for electrocatalytic CO₂ reduction to methane, the electrolyte in both chambers was replaced with fresh, sterile medium free of exogenous organic electron donors, and the cathode headspace was subsequently flushed with 99.999% pure CO₂ to serve as the sole carbon source. These tests were conducted by applying a constant negative potential to the cathode using a CHI1030C electrochemical workstation (CH Instruments, Shanghai, China). Faradaic efficiency for methane production was determined at cathode potentials of −0.8, −0.9, −1.0 and −1.1 V versus Ag/AgCl. No iR compensation was applied to the electrochemical measurements.

### Extraction and analysis of EPS

Prior to extraction, the biohybrid electrodes were gently rinsed with sterile 0.9% NaCl solution to remove loosely associated planktonic cells, and the attached biofilms were subsequently harvested from the electrode fibers. The resulting cell suspension was centrifuged at 5000 × *g* for 10 min at 4 °C to obtain the cell pellets, which were then washed twice with 0.9%(w/v) NaCl solution to eliminate residual medium and impurities, ensuring that the subsequent biomass and activity quantifications accurately reflected the electrode-attached community. EPS was extracted from the biofilms using a heat-based method^16^. The resulting cell pellets were washed twice with a 0.9% (w/v) NaCl solution. After washing, the pellets were resuspended in a fresh 0.9% (w/v) NaCl solution and incubated in a water bath at 60 °C for 30 min to release the EPS. The resulting suspension was centrifuged at 5000 × *g* for 10 min at 4 °C, and the supernatant was passed through a 0.22 µm membrane filter to yield the final EPS extract. The extracted EPS was subsequently analyzed by three-dimensional excitation-emission matrix (3D-EEM) fluorescence spectroscopy. Fluorescence scans were recorded over an excitation wavelength (Ex) range of 200–500 nm and an emission wavelength (Em) range of 200–500 nm.

### Electrodeposition of FeTCPP on the H₂AQC@CF surface

An electrode-based interface, designated H₂AQC@FeTCPP(ED), was fabricated by the potentiostatic deposition of FeTCPP onto an in-situ reduced H₂AQC@CF electrode. The deposition was conducted at −0.8 V (vs. Ag/AgCl) for 20 min in an electrolyte containing 1.0 mM FeTCPP and 0.2 M Na₂SO₄. For comparison, a substrate-free control (H₂AQC@FeTCPP(OC)) was prepared by physically mixing FeTCPP powder with pre-synthesized reduced anthraquinone (H₂AQC) powder. OC denotes the physically mixed control, whereas ED denotes electrodeposition.

### Software and data analysis

Electrochemical measurements were collected using CHI Electrochemical Workstation Software v17.03, and electrochemical impedance spectra were fitted to a Randles equivalent circuit using ZSimpWin v3.60. Scanning electrochemical microscopy data were acquired using VersaSCAN v1.6.6465 and VersaStudio v2.52. Chromatographic data were collected and integrated using T2000P Chromatography Workstation. Quantitative PCR data were collected and analysed using QuantStudio Design and Analysis Desktop Software v1.5.1. Spectroscopic and microscopy data were acquired and processed using the instrument-specific software described in the Supplementary Methods. Numerical data analysis and plotting were performed using OriginPro 2024b (v10.15). RNA-sequencing data were processed using the software and versions specified in the Supplementary Methods. Density functional theory calculations were performed using CP2K v2024.1, with charge analysis conducted using Multiwfn v3.8 and structural visualization performed using VESTA v3.5.8.

### Other methods

Detailed methods for biomass and ATP quantification, microscopy, electrochemical impedance spectroscopy, cyclic voltammetry, scanning electrochemical microscopy, EPS characterization, UV-visible spectroscopy, pyridine hemochrome analysis, LDS-PAGE staining, electron paramagnetic resonance spectroscopy, electrochemical infrared spectroscopy, Raman spectroscopy, X-ray photoelectron spectroscopy, transcriptomic analysis and density functional theory calculations are provided in the Supplementary Methods.

### Statistics and reproducibility

Unless otherwise stated, quantitative data are presented as mean ± standard deviation. For the biomass, ATP and methane-production measurements shown in Figs. 1c, d and 2a, respectively, each group comprised three independently prepared biohybrid cathodes (*n* = 3 biological replicates). Each cathode was treated as one experimental unit. Technical measurements obtained from the same cathode were not treated as independent biological replicates. No statistical method was used to predetermine sample size. Formal randomization and blinding were not performed because group allocation was determined by electrode composition and applied potential. All groups were prepared and analyzed using the same predefined procedures.

The SEM experiment shown in Fig. 1b was independently repeated three times with similar results. No hypothesis-testing procedure was applied unless explicitly stated. Quantitative differences are therefore reported descriptively.

### Reporting summary

Further information on research design is available in the Nature Portfolio Reporting Summary linked to this article.

## Supplementary information

Supplementary Information

Reporting Summary

Transparent Peer Review file

## Source data

Source_Data

## Data Availability

The raw transcriptomic sequencing data of *Methanosarcina barkeri* generated in this study have been deposited in the NCBI Sequence Read Archive under BioProject accession code PRJNA1466089. The numerical data underlying the main and Supplementary Figs. generated in this study are provided in the Source Data file. Source data are provided with this paper. No data are subject to access restrictions. All data supporting the findings of the manuscript are available from the corresponding author upon request. Source data are provided with this paper.
