# Supplementary material for: Quinone-heme π-π interactions bridge the biotic-abiotic interface for enhanced methanogenesis
Source: Nat Commun. 2026 Sep 1;17:10383. doi: 10.1038/s41467-026-77276-8 (PMC13627723; doi:10.1038/s41467-026-77276-8)
Supplement: Supplementary file 1 [file 41467_2026_77276_MOESM1_ESM.pdf]

Supplementary Information

Quinone-heme π-π interactions bridge the biotic-abiotic interface for enhanced methanogenesis

Supplementary Methods

Unless otherwise stated, all chemical reagents were purchased from Aladdin Biochemical Technology Co., Ltd. (Shanghai, China) and were of analytical grade. AQC (anthraquinone-2-carboxylic acid, ⩾99%), EDC, NHS, FeTCPP, dopamine hydrochloride, and sodium dithionite were used without further purification

Quantitative PCR Test (total biomass): The genomic DNA of three hybrid samples was extracted, and the target *mcrA* gene was amplified by PCR pre-experiment using specific primers (F: 5'-TTCGGTGGATCDCARAGRGC-3', R: 5'-GBARGTCGWAWCCGTAGAATCC-3'). Amplicons were extracted from 2% agarose gels, purified using the AxyPrep DNA Gel Extraction Kit (Axygen Biosciences, Union City, CA, U.S.) according to the manufacturer's instructions, and quantified using QuantiFluor-ST (Promega, U.S.). The constructed plasmid was then linearized, converted into a copy number (copies/μL) by formula, and a 10-fold gradient dilution (typically from 10^{-2} to 10^{-6}) was prepared to generate a standard curve. Quantitative PCR was performed in a total reaction volume of 30 μL containing 15 μL of qPCR Mix, 2 μL of Mg^{2+} solution (25 mM), 0.5 μL of each primer (10 μM), 0.5 μL of fluorescent dye, 1 μL of template DNA, and 10.5 μL of nuclease-free water. The reaction program consisted of 95°C for 3 min, followed by 30 cycles at 94 °C for 30 s and 50 °C for 30 s. Quantitative PCR was performed using a QuantStudio 5 Real-Time PCR System (Applied Biosystems, Thermo Fisher Scientific, USA). Fluorescence data, threshold-cycle values, melting curves and standard curves were acquired and analysed using QuantStudio Design and Analysis Desktop Software v1.5.1. Absolute *mcrA* copy numbers were calculated from the plasmid standard curve and normalized to the projected electrode area (copies cm^{-2}). For presentation in Fig. 1c, the area-normalized

mcrA copy number of each group was further normalized to that of the M. b-CF group, which was set to 1.

ATP content: ATP content was determined using a micro-method ATP assay kit (Solarbio, Beijing, China) based on the phosphor-molybdenum colorimetric method. Briefly, the *Methanosarcina barkeri* biofilm samples were collected and resuspended in an acidic extraction solution. The suspension was disrupted via ultrasonication (ice bath, 20% intensity) and centrifuged at 8000 g for 10 min at 4°C. The supernatant was collected and neutralized with an equal volume of alkaline extraction solution, followed by a second centrifugation under the same conditions to obtain the test sample. For the assay, the sample supernatant was mixed with the working solution containing creatine kinase and incubated at 37°C for 30 min to facilitate the conversion of creatine and ATP into phosphocreatine. Subsequently, a chromogenic reagent was added, and the mixture was incubated at 37°C for another 20 min. The absorbance was measured at 700 nm using a microplate reader. The ATP content was calculated based on the absorbance difference between the sample and control tubes, using a standard ATP solution (2 μmol/mL) for calibration.

Methane quantification: methane concentrations in the reactor headspace were measured using a GC-7900 gas chromatograph equipped with a flame-ionization detector (Shanghai Techcomp Instrument Co., Ltd., China). Chromatographic peaks were identified, baseline-corrected and integrated using T2000P Chromatography Workstation. Methane concentrations were quantified using an external calibration curve prepared from certified methane standards. The methane production rate and Faradaic efficiency were calculated as described below.

Scanning electron microscopy (SEM): For morphological analysis, the harvested cathodes were first pretreated. The samples were fixed overnight in a 2.5% glutaraldehyde solution at 4°C. Subsequently, they were washed three times (10 min each wash) with a 0.1 M phosphate buffer solution (containing 0.13 M NaCl and 10 mM Na_2HPO_4, pH 7.2). A graded dehydration series was then performed by immersing

the samples for 10 min each in 50%, 70%, 80%, 90%, and 100% tert-butanol/water mixtures, followed by three additional immersions in 100% tert-butanol. Finally, the samples were freeze-dried and sputter-coated with gold before being observed with a scanning electron microscope. Following freeze-drying and gold sputter coating, the samples were examined using an SU8600 field-emission scanning electron microscope (Hitachi High-Tech, Japan). Instrument operation and image acquisition were performed using SU8600 PC-SEM Operating Software and HD Capture, respectively.

Cyclic Voltammetry and Electrochemical Impedance Spectroscopy: Cyclic voltammetry and electrochemical-impedance-spectroscopy measurements were performed using a CHI980 electrochemical workstation (Shanghai Chenhua Instrument Co., Ltd., China) controlled by CHI Electrochemical Workstation Software v17.03. EIS measurements were conducted in a standard three-electrode system using a redox-probe electrolyte containing 5 mM $K_3[Fe(CN)_6]/K_4[Fe(CN)_6]$ and 1 M KCl. Spectra were recorded over a frequency range of 0.01 Hz to 1.0×10^5 Hz using a sinusoidal perturbation amplitude of 10 mV. No iR compensation was applied. The impedance spectra were fitted to a Randles equivalent circuit using ZSimpWin v3.60.

Scanning electrochemical microscope: Scanning electrochemical microscopy was performed using a VersaSCAN scanning electrochemical microscope (Advanced Measurement Technology/AMETEK, USA). Instrument control, approach-curve measurements and XY scanning were performed using VersaSCAN v1.6.6465 and VersaStudio v2.52. A platinum microelectrode with a diameter of 10 μm was used as the probe, with a platinum sheet as the counter electrode and an Ag/AgCl electrode as the reference electrode. The electrolyte contained 25 mM potassium ferricyanide in 0.1 M KCl. The probe-to-biofilm distance was determined using a negative-feedback approach curve. A 500 μm × 500 μm region was scanned at 10 μm s^{-1}, with the substrate and probe potentials maintained at −0.8 and +0.4 V versus Ag/AgCl, respectively. Two- and three-dimensional current-response maps were generated from the acquired data, and secondary plotting was performed using OriginPro 2024b (v10.15).

Cyclic voltammograms of the EPS: The electrochemical measurements of the extracted EPS were performed on an electrochemical workstation using a three-

electrode system. A glassy carbon (GC) electrode, a Pt sheet, and an Ag/AgCl electrode were utilized as the working, counter, and reference electrodes, respectively. Prior to measurement, the GC electrode was modified to ensure adhesion: 0.05 mL of Nafion (5 wt%) was mixed with 2.95 mL of deionized water and sonicated for 30 min. The GC electrode was treated with this mixture and dried at 25 °C. Subsequently, 0.04 mL of the extracted EPS solution was dropped onto the modified GC working electrode. The analysis was conducted in a 0.1 M KCl electrolyte solution. Cyclic voltammetry was performed with a scanning window from -1.0 V to +0.2 V at a scan rate of 50 mV/s for 6 cycles, with a total measuring time of 480 s.

UV-Vis of EPS: UV–visible spectra were collected using a UV-2600i UV–Vis spectrophotometer (Shimadzu, Japan) controlled by LabSolutions UV-Vis v1.12. Spectra of the isolated EPS fractions were recorded from 300 to 600 nm and baseline-corrected against the corresponding buffer blank. Pyridine hemochrome spectra were recorded from 400 to 600 nm before and immediately after reduction with excess sodium dithionite. Blank subtraction and absorption-peak identification were performed using LabSolutions UV-Vis v1.12, and secondary plotting was performed using OriginPro 2024b (v10.15).

Pyridine Hemochrome Assay: The presence and identity of heme in isolated EPS were further confirmed by the pyridine hemochrome assay. Briefly, EPS samples were mixed with pyridine (final concentration 20%, v/v) and NaOH (final concentration 0.1 M) to release heme from any residual protein scaffold and stabilize it as the pyridine hemochrome complex. The absorption spectrum of this oxidized pyridine hemochrome was first recorded between 400-600 nm. Sodium dithionite ($Na_2S_2O_4$) was then added in excess to fully reduce the sample, and the reduced spectrum was immediately recorded. The characteristic α-band at 556 nm and β-band at 524 nm in the reduced spectrum confirm the presence of heme b.

LDS-PAGE: EPS samples from the three biohybrid groups were mixed with NuPAGE LDS Sample Buffer (4×) and incubated at room temperature for 10 min. Proteins were separated on a NuPAGE 4–12% Bis-Tris gel using MES running buffer at 200 V for 35

min at 4°C. Following electrophoresis, heme-specific staining was performed using 3,3',5,5'-tetramethylbenzidine (TMB). Briefly, the gel was soaked in a TMB solution (6.3 mM TMB in 30% methanol, 0.25 M sodium acetate, pH 5.0) for 30 min in the dark with gentle agitation, followed by the addition of H_2O_2 to a final concentration of 30 mM to initiate the chromogenic reaction. Heme-containing bands appeared as blue-green signals due to the peroxidase activity of heme. The reaction was stopped by transferring the gel to distilled water. A pre-stained protein ladder was used as the molecular weight reference.

Electron Paramagnetic Resonance: EPR spectra were recorded using an A200-9.5/12 X-band EPR spectrometer (Bruker BioSpin, Germany). Data acquisition, baseline correction, g-value calculation and spectral integration were performed using Bruker Xenon v1.1b, with spectral simulations or fitting conducted using the SpinFit module where applicable. Samples were measured at 99 K using a microwave frequency of 9.51 GHz, microwave power of 2.25 mW, modulation frequency of 100 kHz, modulation amplitude of 5.00 G, time constant of 81.92 ms and conversion time of 80.00 ms. The magnetic field was scanned from 200 to 4200 G.

Electrochemical infrared spectroscopy: Electrochemical infrared spectroscopy was performed using the biohybrid cathode as the working electrode, a platinum sheet as the counter electrode, and an Ag/AgCl electrode as the reference electrode. The biohybrid working electrode was placed in a thin-layer spectroelectrochemical cell equipped with a CaF_2 window. The cell contained 0.1 M KCl prepared in D_2O to minimize interference from water absorption. The working-electrode potential was varied from +0.2 to −0.8 V versus Ag/AgCl in 0.2 V intervals. Fourier-transform infrared spectra were recorded over the range of 500–4500 cm^{-1} and processed using OMNIC 8.0.

Density functional theory analysis: Density functional theory calculations were performed using the Quickstep module of CP2K v2024.1. Geometry optimizations employed the revised Perdew–Burke–Ernzerhof exchange–correlation functional with Grimme DFT-D3(BJ) dispersion correction. Goedecker–Teter–Hutter pseudopotentials

and molecularly optimized Gaussian basis sets were used, with a plane-wave cutoff of 400 Ry and a relative cutoff of 55 Ry. Input structures were constructed using Multiwfn v3.8. Charge transfer was quantified using the Charge Model 5 method implemented in Multiwfn v3.8. Structural models and charge-density-difference isosurfaces were visualized using VESTA v3.5.8. Goedecker-Teter-Hutter (GTH) pseudopotentials were employed for all atoms to treat core electrons, with valence electrons described using double-ξ valence polarized (DZVP) molecularly optimized basis sets: DZVP-MOLOPT-SR-GTH-q4 for carbon, DZVP-MOLOPT-SR-GTH-q1 for hydrogen, and DZVP-MOLOPT-SR-GTH-q6 for oxygen during geometry optimization, while triple-ξ valence polarized (TZVP) basis sets were used for single point energy calculations. The plane-wave cutoff for the auxiliary basis was set to 400 Ry, with a relative cutoff of 55 Ry for the multigrid integration. The Martyna-Tuckerman Poisson solver was used for electrostatics under XY-periodic conditions. All input files were generated using Multiwfn software, images were rendered by VESTA software. Periodic boundary conditions were applied in the XY directions to simulate a 2D infinite slab, with a vacuum space along the Z-axis to prevent interlayer interaction.

Transcriptomic Analysis: Total RNA was extracted from the biofilm samples and sequenced as described above. One pooled RNA-seq library, generated from three independently prepared cathodes, was analysed for each condition.. Clean reads were aligned to the complete genome of *Methanosarcina barkeri* strain MS (DSM 800; GenBank accession CP009528), and transcript abundance was summarized as reads per kilobase of transcript per million mapped reads (RPKM). Because biological replicates were not available, within-group biological variability and dispersion could not be estimated. Therefore, no formal differential-expression testing, P values or false-discovery rates were calculated. The transcriptomic data were used only for descriptive and hypothesis-generating comparisons among the three sequenced samples.

X-ray photoelectron spectroscopy and Raman spectroscopy: X-ray photoelectron spectroscopy (XPS) measurements were performed using an AXIS Supra+ X-ray photoelectron spectrometer (Kratos Analytical, UK; Shimadzu Group) controlled by

ESCApe v1.5. XPS spectra were processed and analysed using CasaXPS v2.3.26. Raman spectra were collected using an inVia Qontor confocal Raman microscope (Renishaw, UK) controlled by WiRE v5.5. Raman spectral processing and analysis were also performed using WiRE v5.5.

The methane production rate and Faradaic efficiency for methane production were calculated as follows:

$$r_{\mathrm{CH_4}} = \frac{\Delta n_{\mathrm{CH_4}}}{A\Delta t}$$

$$\mathrm{FE}_{\mathrm{CH_4}} = \frac{8Fn_{\mathrm{CH_4}}}{|Q|} \times 100\%$$

where r_{CH_4} is the methane production rate; Δn_{CH_4} is the amount of methane produced; A is the projected cathode area; Δt is the reaction time; F is the Faraday constant (96,485 C mol^{-1}); and Q is the total charge passed during the corresponding time interval.

Supplementary Figures

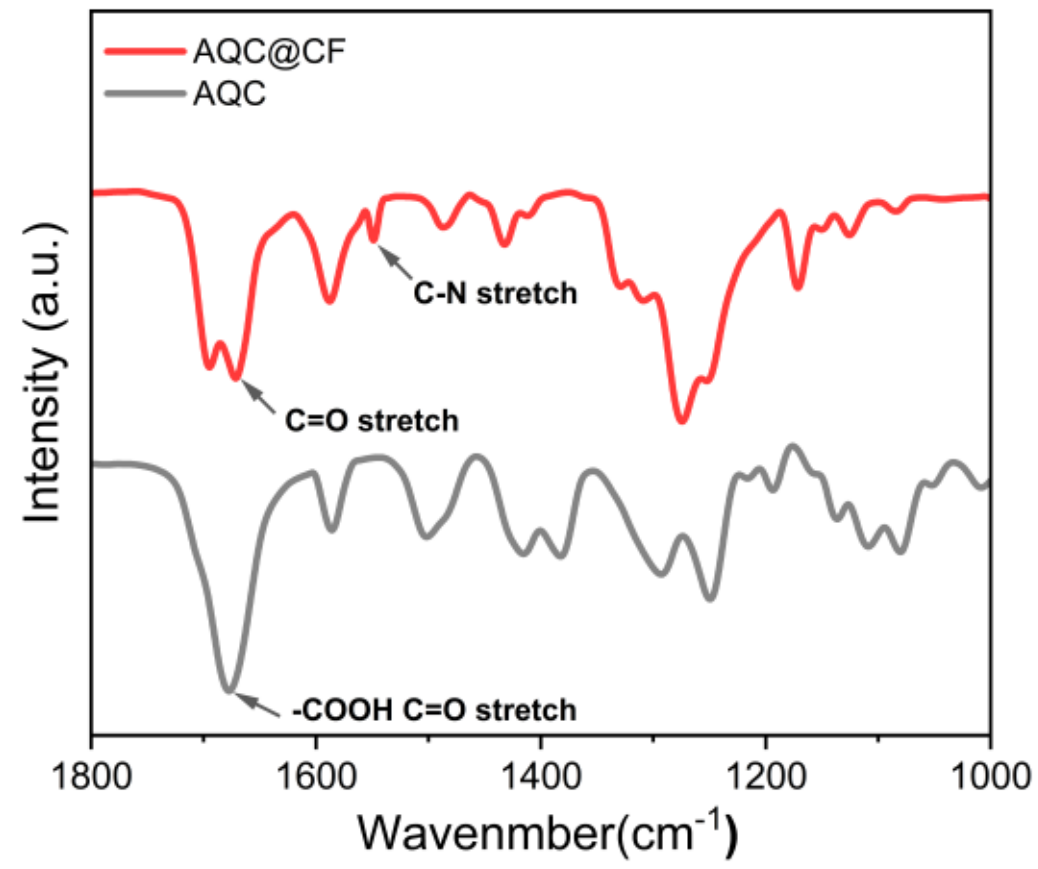

Supplementary Fig. 1. Fourier-transform infrared (FTIR) spectra of anthraquinone-2-carboxylic acid (AQC) and the AQC-functionalized carbon felt electrode (AQC@CF). Grey and red curves represent AQC and AQC@CF, respectively. Source data are provided as a Source Data file.

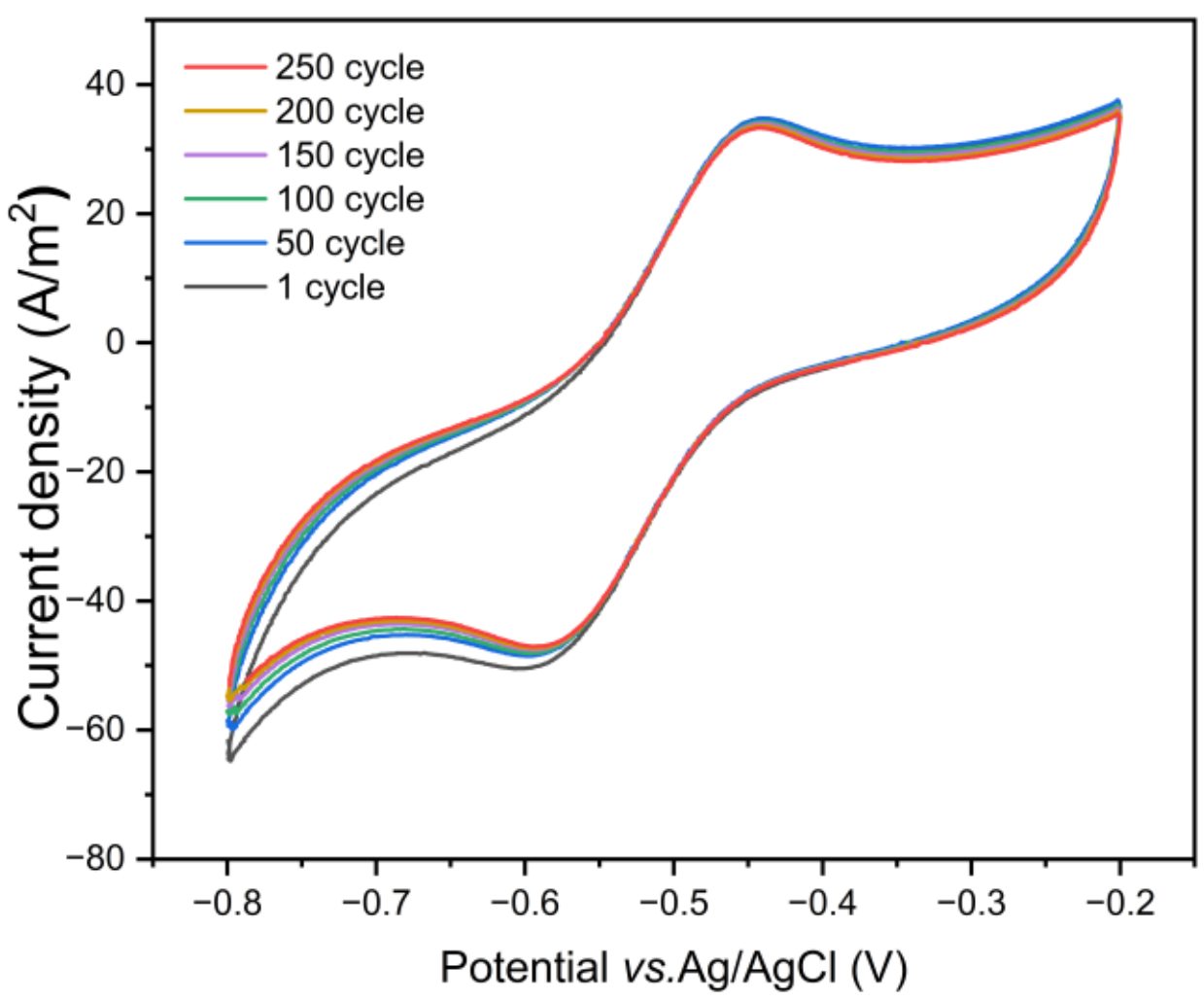

Supplementary Fig. 2. Cyclic voltammetry (CV) stability test of the AQC@CF electrode. Line colours denote the corresponding cycle numbers indicated in the figure key. Potentials are reported versus an Ag/AgCl reference electrode. Source data are provided as a Source Data file.

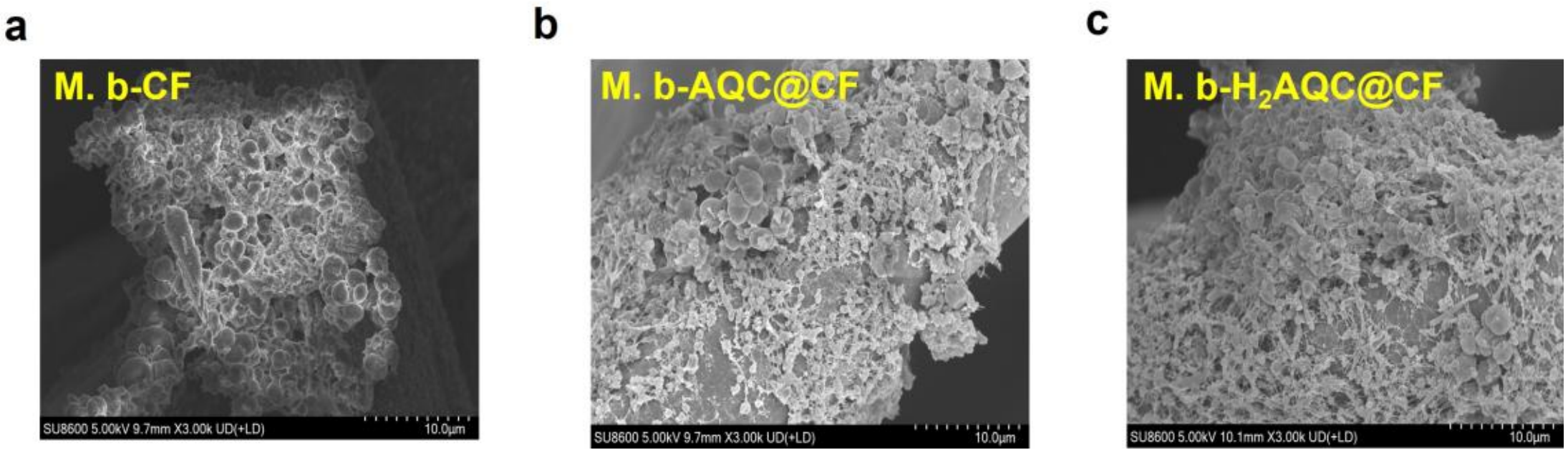

Supplementary Fig. 3. Scanning electron microscopy (SEM) images of M. barkeri biofilms colonized on pristine CF (a), AQC@CF (b) and reduced AQC@CF (H_2AQC@CF) (c) electrodes after 30 days of cultivation.

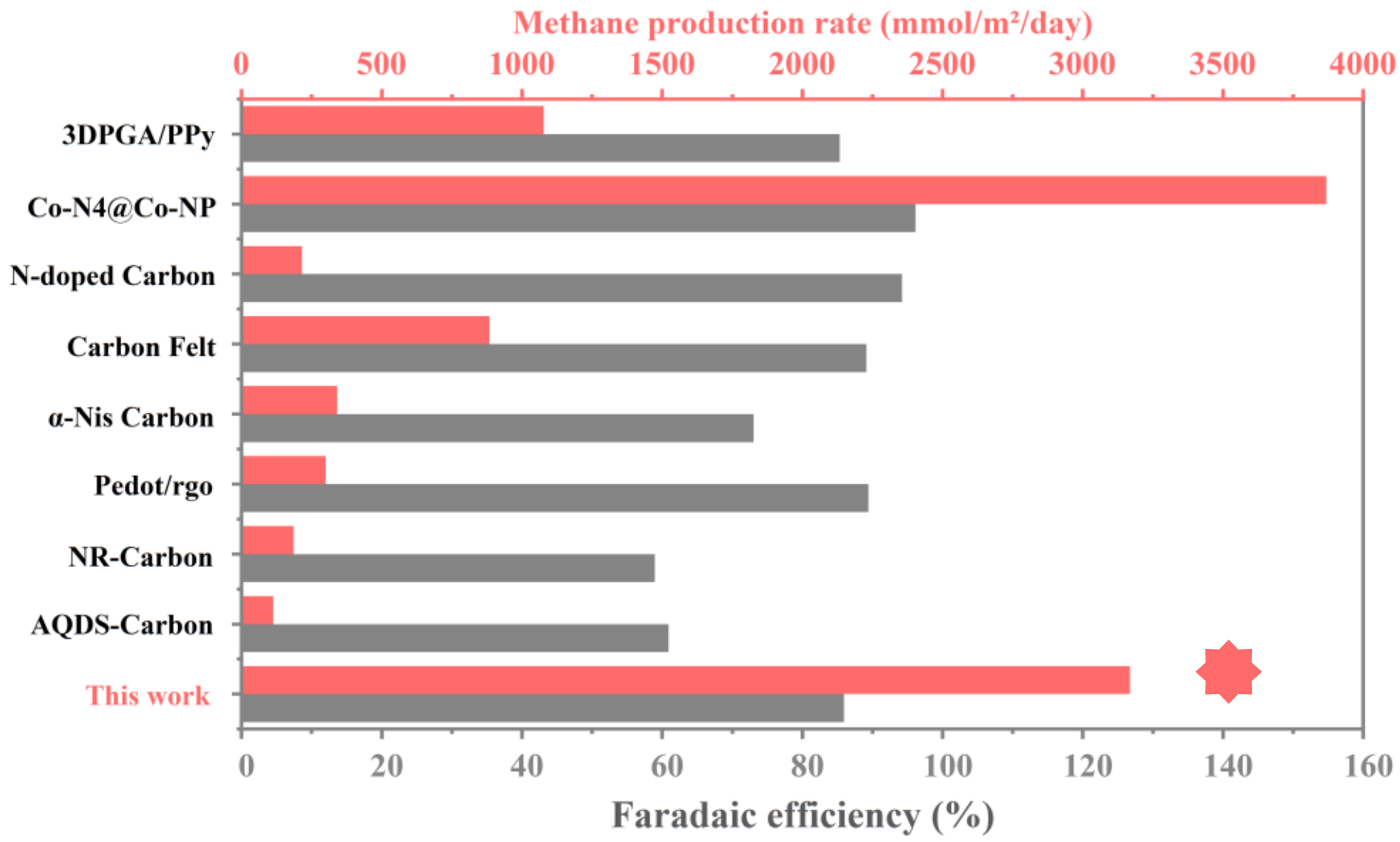

Supplementary Fig. 4. Comparison of methane production rates and Faradaic efficiencies obtained in this study with previously reported values. Grey bars indicate Faradaic efficiency, red bars indicate methane production rate, and the star identifies this study. 3DPGA/PPy, three-dimensional-printed graphene aerogel/polypyrrole; Co–N_4@Co–NP, Co–N_4 sites supported on cobalt nanoparticles; PEDOT/rGO, poly(3,4-ethylenedioxythiophene)/reduced graphene oxide; NR, neutral red; AQDS, anthraquinone-2,6-disulfonate. Details and references are provided in Table S2.

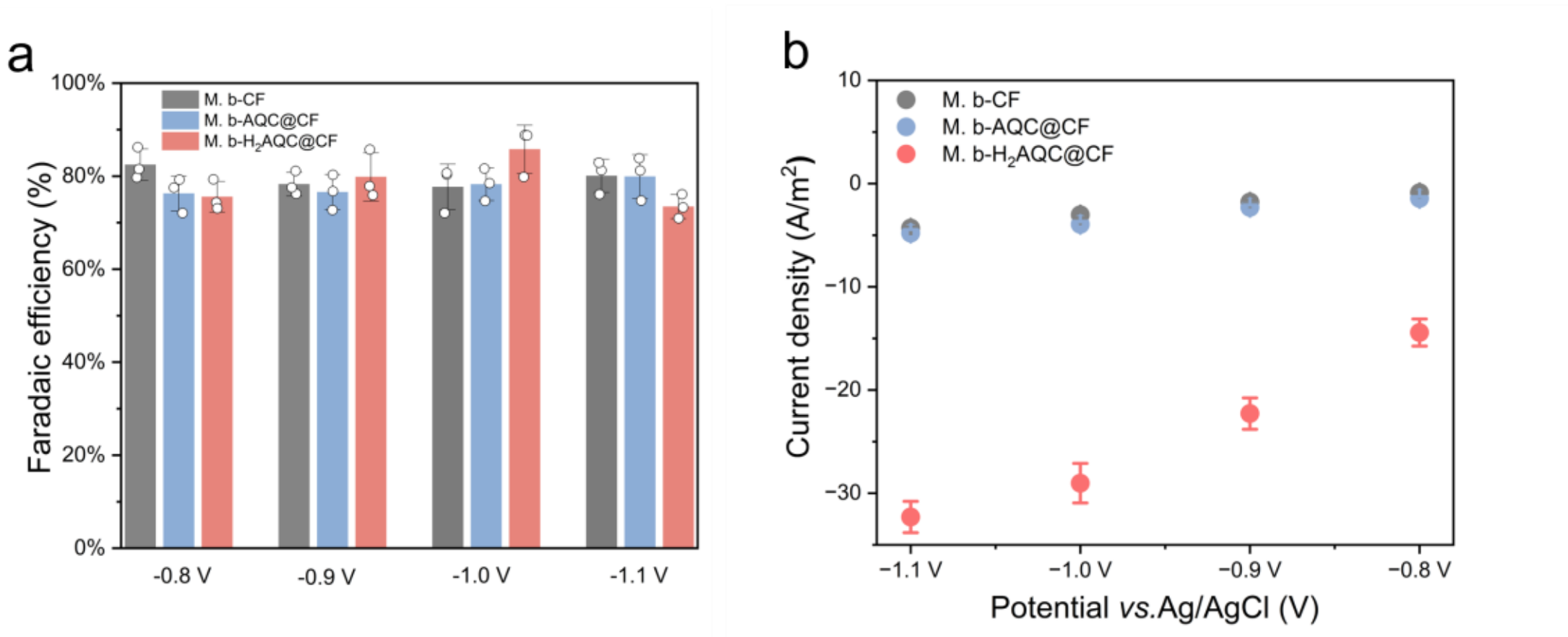

Supplementary Fig. 5. Faradaic efficiency (a) and current density (b) of the three biohybrid groups. Grey, blue and red represent M. b-CF, M. b-AQC@CF and M. b-H_2AQC@CF, respectively. Data are presented as mean ± SD; circles represent individual biological replicates (n = 3 independently prepared biohybrid cathodes per group). Source data are provided as a Source Data file.

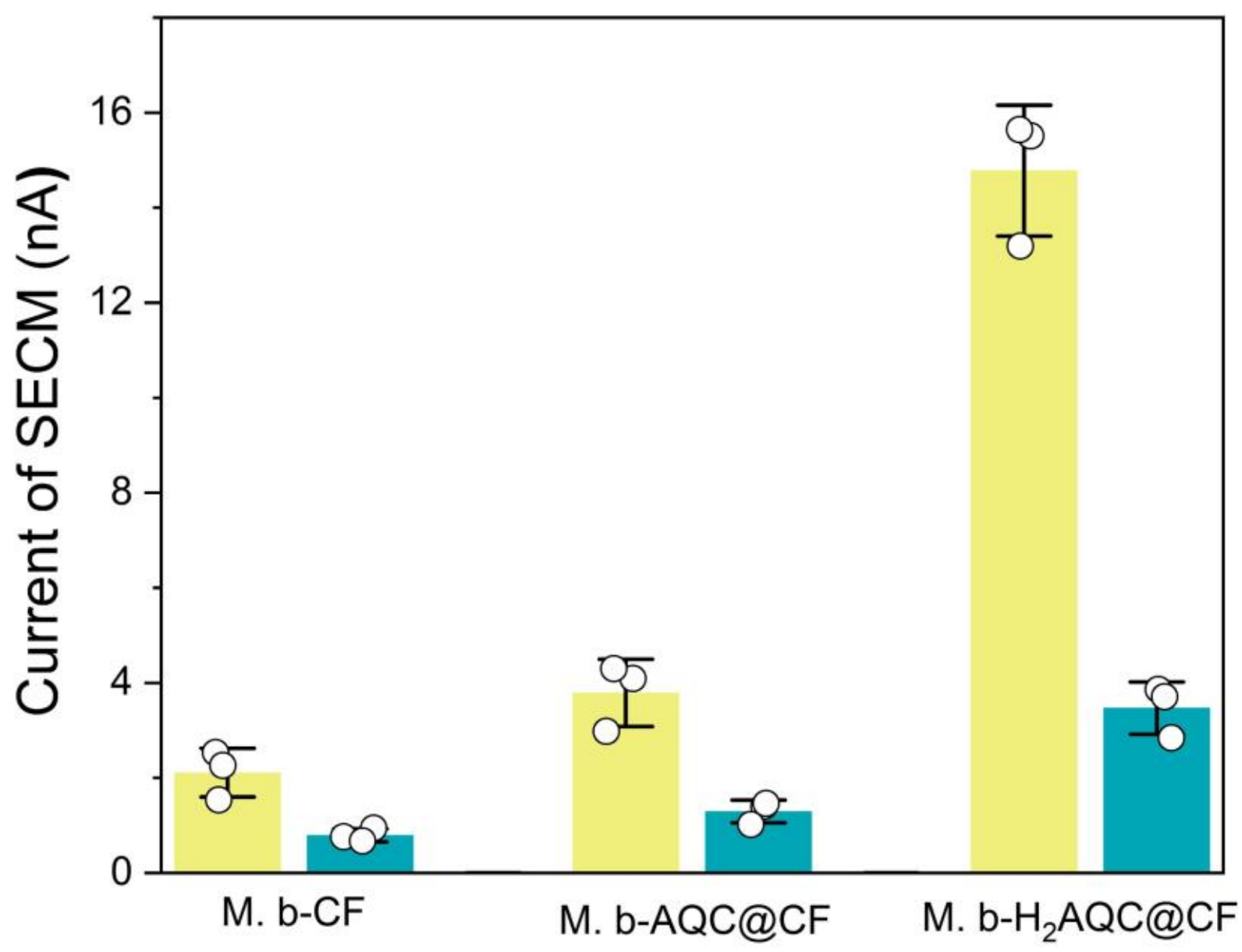

Supplementary Fig. 6. Maximum and average scanning electrochemical microscopy (SECM) currents of the three biohybrids at -0.8 V. Yellow and teal bars indicate maximum and average currents, respectively. Data are presented as mean ± SD; circles represent individual biological replicates (n = 3 independently prepared biohybrid cathodes per group). Source data are provided as a Source Data file.

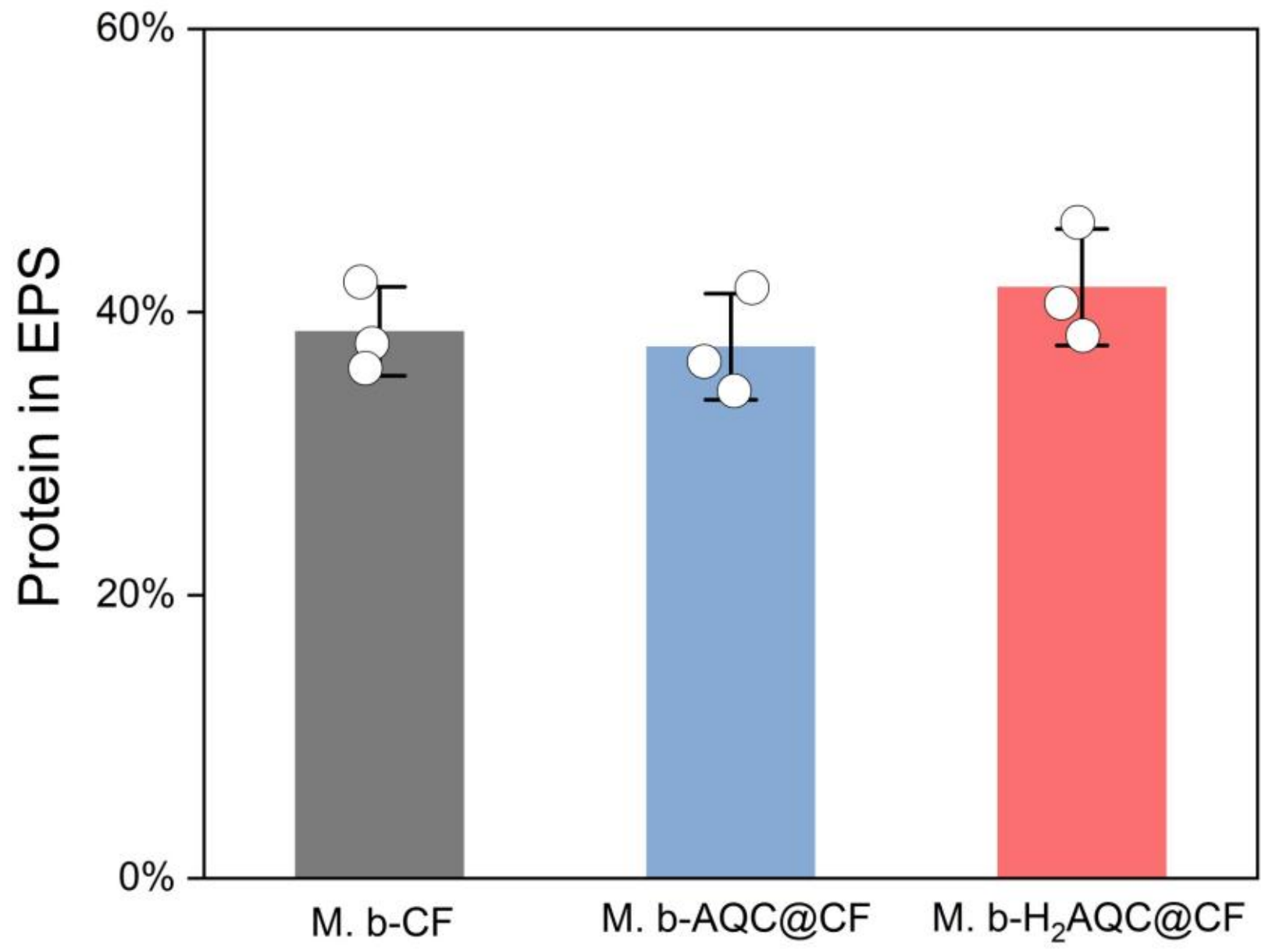

Supplementary Fig. 7. Protein content in extracellular polymeric substances (EPS) extracted from the three *M. barkeri* biohybrids. Grey, blue and red represent M. b-CF, M. b-AQC@CF and M. b-H_2AQC@CF, respectively. Data are presented as mean ± SD; circles represent individual biological replicates ($n = 3$ independently prepared biohybrid cathodes per group). Source data are provided as a Source Data file.

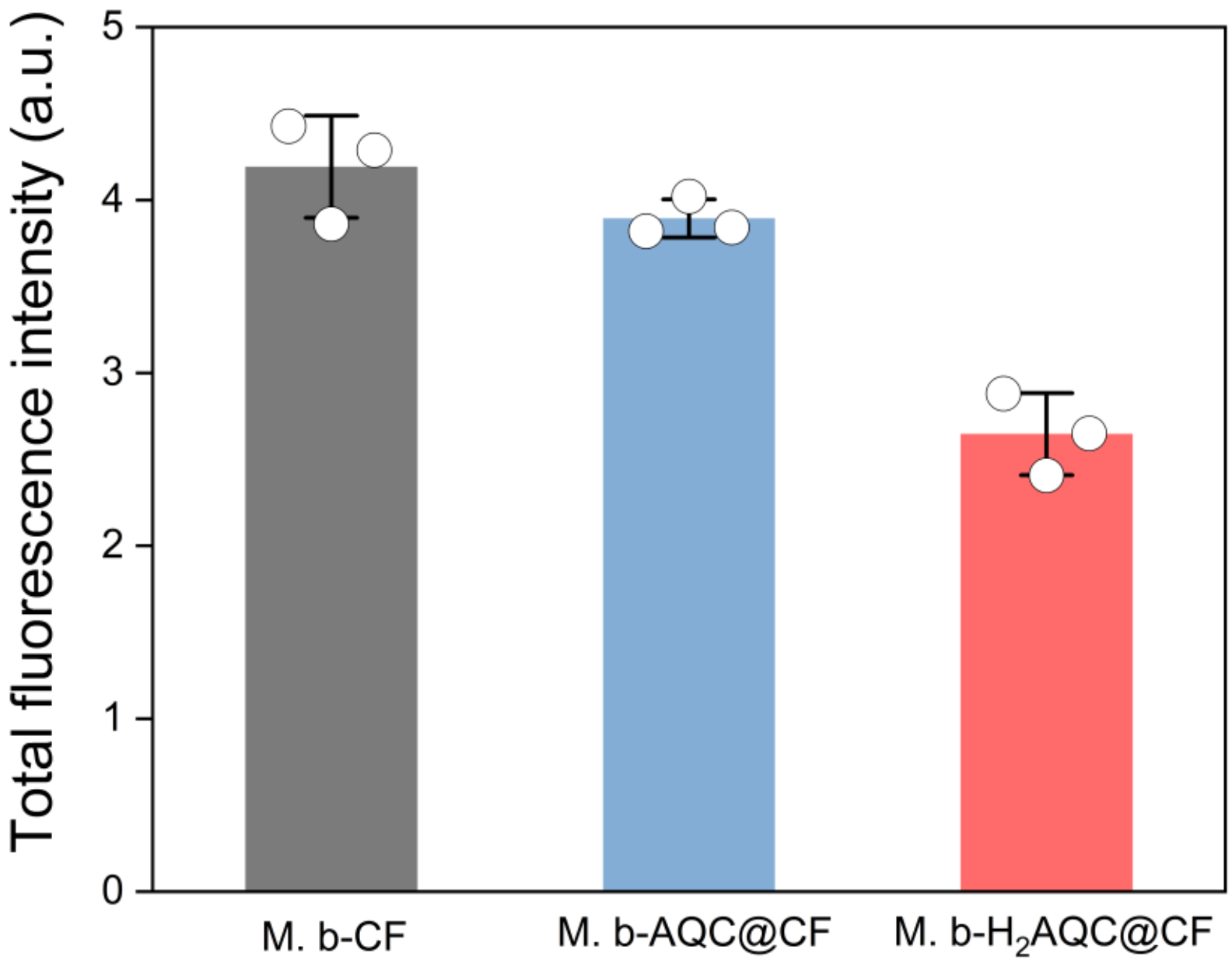

Supplementary Fig. 8. Total fluorescence intensity of aromatic protein-like substances in extracellular polymeric substances (EPS) extracted from the three biohybrids. Grey, blue and red represent M. b-CF, M. b-AQC@CF and M. b-H_2AQC@CF, respectively. Data are presented as mean ± SD; circles represent individual biological replicates (n = 3 independently prepared biohybrid cathodes per group). Source data are provided as a Source Data file.

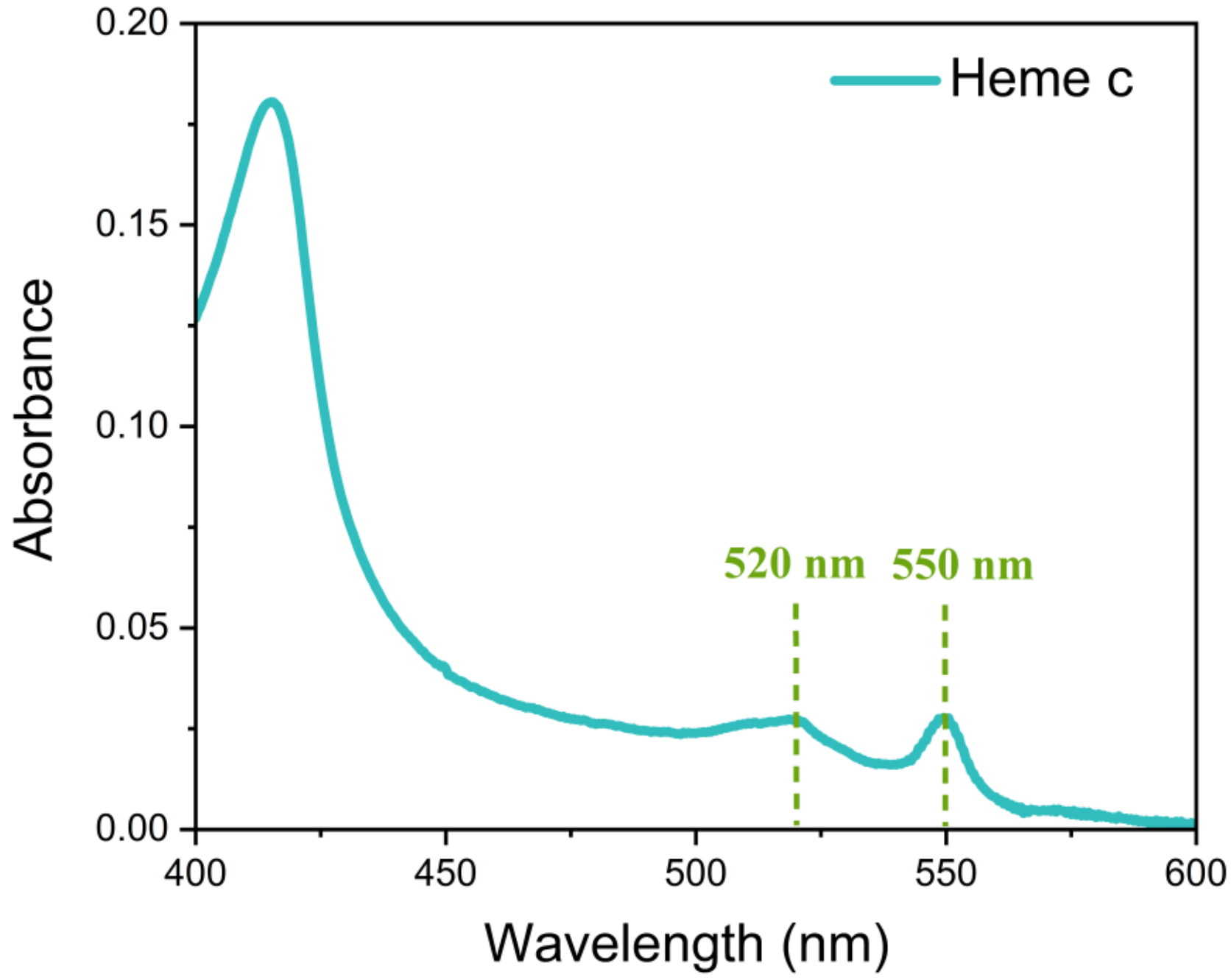

Supplementary Fig. 9. Ultraviolet-visible absorption spectrum of bovine heart cytochrome c, showing the β and α absorption bands at 520 and 550 nm, respectively. Source data are provided as a Source Data file.

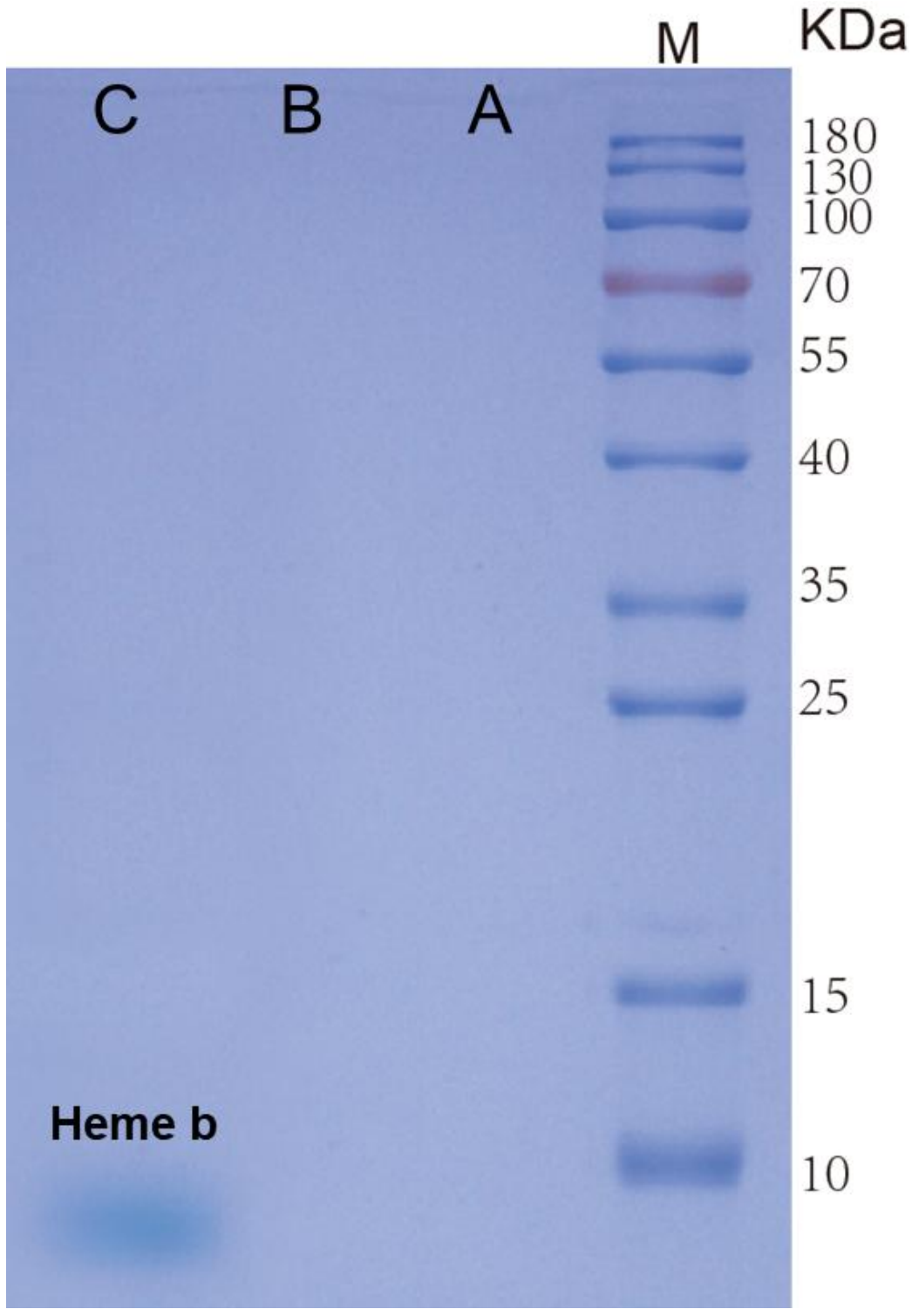

Supplementary Fig. 10. Lithium dodecyl sulfate-polyacrylamide gel electrophoresis (LDS-PAGE) heme-specific staining of extracellular polymeric substances (EPS) from the three biohybrid groups. Lane A, M. b-CF; lane B, M. b-AQC@CF; lane C, M. b-H_2AQC@CF; M, molecular-mass marker.

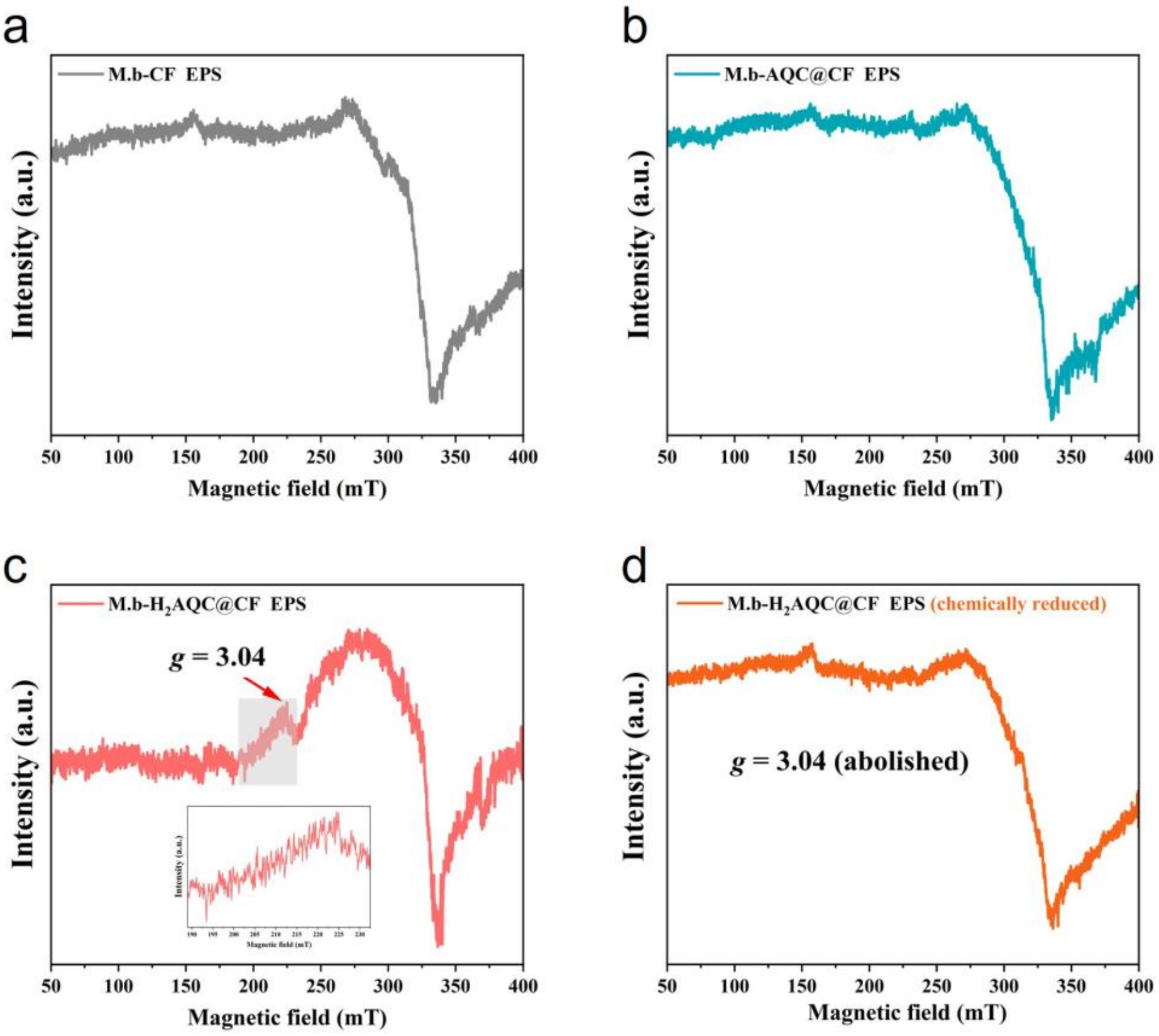

Supplementary Fig. 11. Electron paramagnetic resonance (EPR) spectra of EPS from M. b-CF (a), M. b-AQC@CF (b), M. b-H_2AQC@CF (c), and M. b-H_2AQC@CF after chemical reduction with sodium dithionite (d). The signal at g = 3.04 is consistent with low-spin ferric (Fe^{3+}) heme and was abolished after chemical reduction. Arrows indicate the position of this signal. g, spectroscopic splitting factor; Source data are provided as a Source Data file.

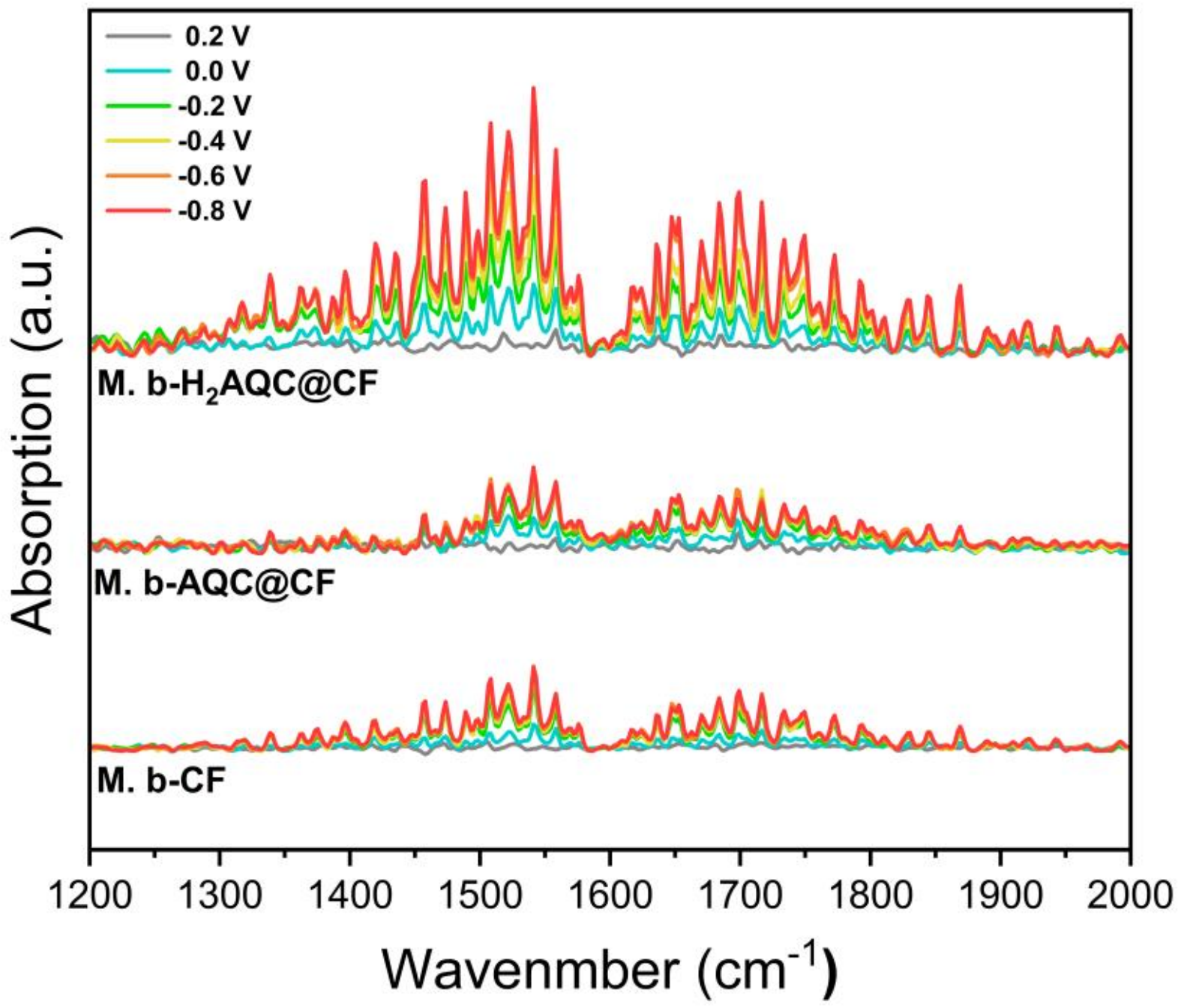

Supplementary Fig. 12. Electrochemical infrared spectra of the three biohybrids recorded at applied potentials from +0.2 to −0.8 V versus Ag/AgCl. Line colours indicate the potentials specified in the figure key. Source data are provided as a Source Data file.

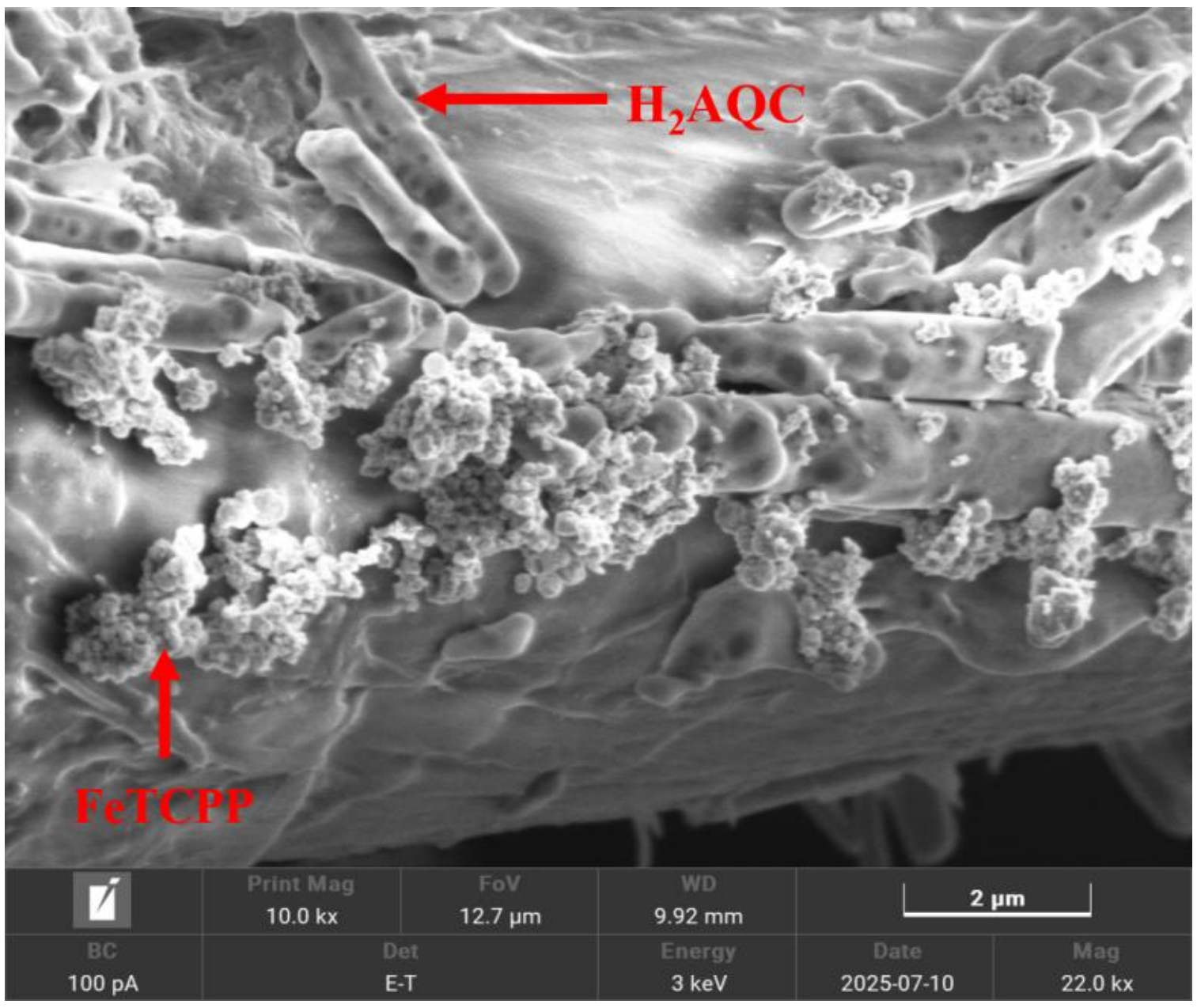

Supplementary Fig. 13. Scanning electron microscopy (SEM) image of FeTCPP electrodeposited on the H_2AQC@CF surface. Red arrows indicate the H_2AQC@CF substrate and electrodeposited FeTCPP regions.

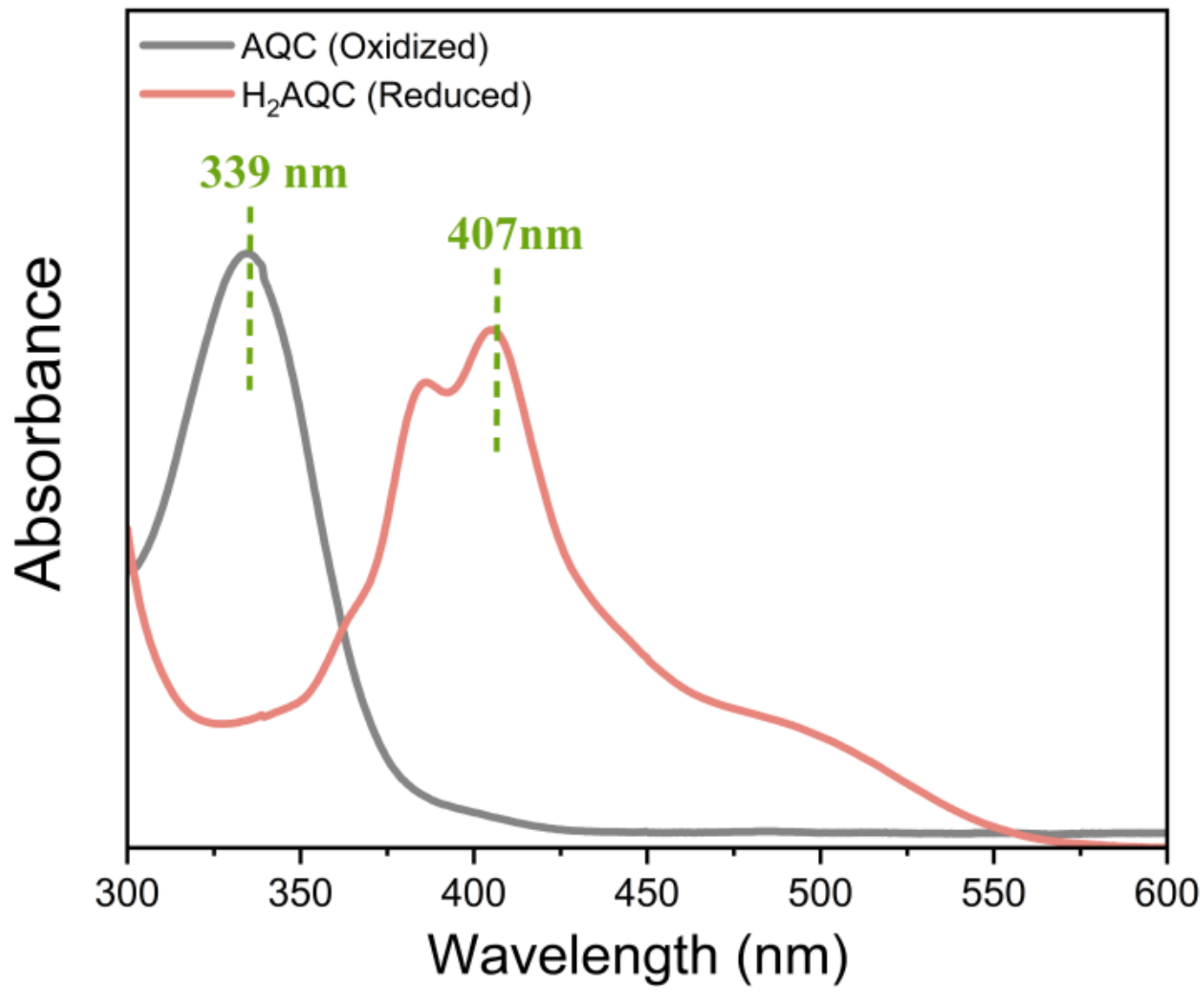

Supplementary Fig. 14. Ultraviolet-visible (UV–Vis) spectra of oxidized AQC and reduced H_2AQC, with absorption maxima at 339 and 407 nm, respectively. Source data are provided as a Source Data file.

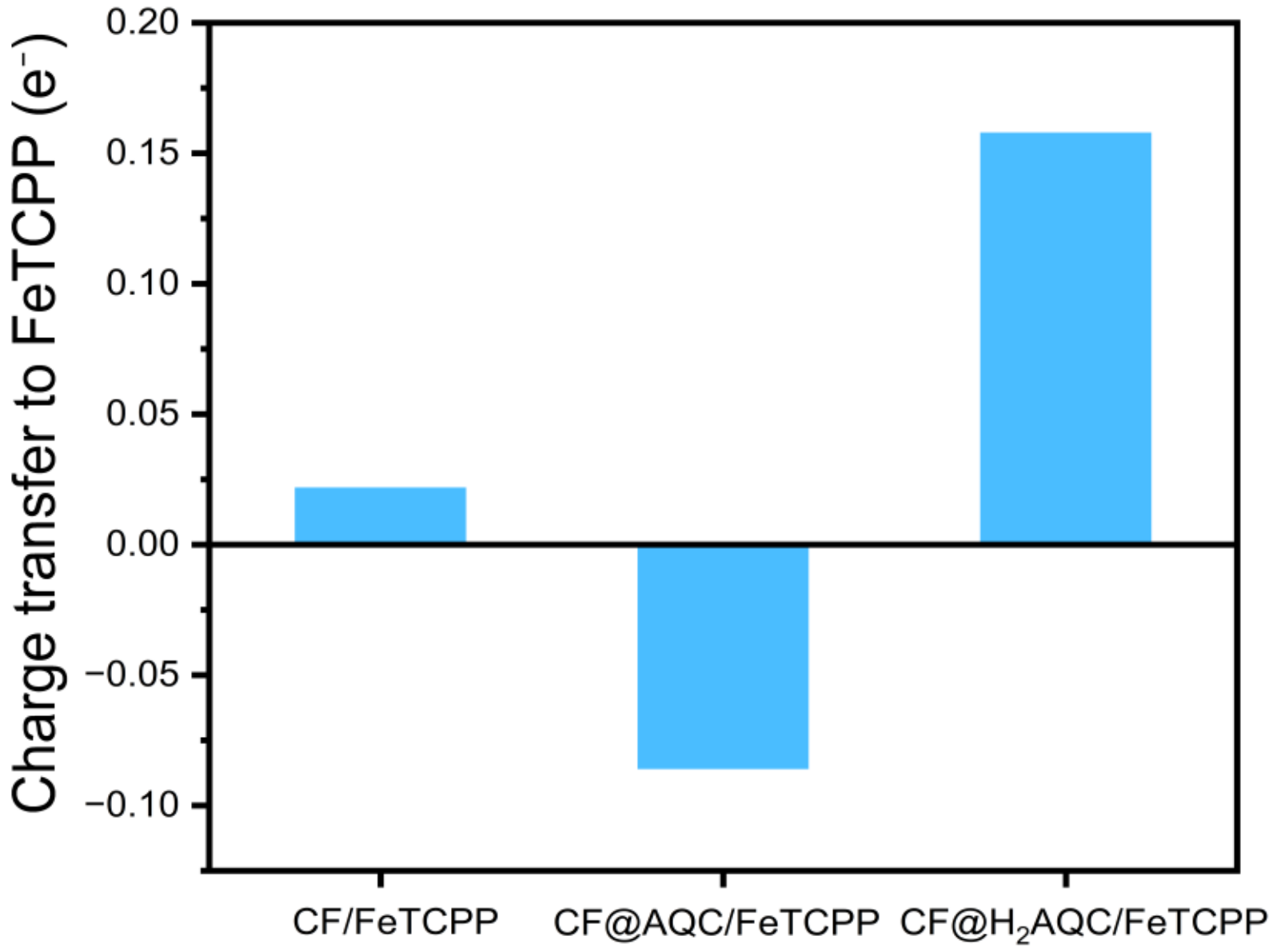

Supplementary Fig. 15. Net charge transferred to FeTCPP in the three molecular systems, calculated using the Charge Model 5 (CM5) method. Source data are provided as a Source Data file.

Supplementary Tables

Table S1. Synthetic medium for *M.barkeri* biohybrids (500 mL).

Components	Amount
KH_2PO_4	0.115 g
K_2HPO_4	0.23 g
NH_4Cl	0.25 g
$MgSO_4 \cdot 7H_2O$	0.125 g
CaCl2·$2H_2O$	0.125 g
$FeSO_4 \cdot 7H_2O$	1 mL
trace element solution	5 mL
vitamin solution	5 mL
Yeast extract	1 g
Casitone	1 g
Methanol	5 mL
$NaHCO_3$	1 g
Resazurin	1 mL
L-cysteine	3 g
$Na_2S \cdot 9H_2O$	1.5 g

Table S2. Comparison of methane production rate in MES systems with cathodes modified by different materials.

**Materials**	**Culture**	**Potential (vs. Ag/AgCl)**	**Production rate (mmol m^{-2} day^{-1})**	**Faradaic efficiency**	**Reference**
3DPGA/PPy	Mixed	-1.0 V	1077	**85.3%**	(1)
Co-N4@Co-NP	*M. barkeri*	-0.9 V	3860	**95.1%**	(2)
N-doped Carbon	Mixed	-0.9 V	215	**94.2%**	(3)
Carbon Felt	Mixed	-1.0 V	883	**89.1%**	(4)
α-Nis Carbon	*M. barkeri*	-1.0 V	341	**73.0%**	(5)
Pedot/rgo	Mixed	-0.9 V	300	**89.4%**	(6)
NR-Carbon	Mixed	-1.0 V	185	**58.9%**	(7)
AQDS-Carbon	Mixed	-1.0 V	112	**60.9%**	(7)
**AQC@CF**	*M. barkeri*	-1.0 V	**2787**	**85.8%**	**This work**

Supplementary references

1. He, Y. et al. 3D-printed GA/PPy aerogel biocathode enables efficient methane production in microbial electrosynthesis. Chem. Eng. J. 459, 141523 (2023).
2. Xia, R. et al. Revealing $Co–N_4$@Co–NP bridge-enabled fast charge transfer and active intracellular methanogenesis in bio-electrochemical CO_2 conversion with Methanosarcina barkeri. Adv. Mater. 35, 2304920 (2023).
3. Xia, R. et al. Atomic pyridinic nitrogen as highly active metal-free coordination sites at the biotic–abiotic interface for bio-electrochemical CO_2 reduction. Small 20, 2306331 (2024).
4. Fu, Q. et al. Direct CO_2 delivery with a hollow stainless-steel/graphene-foam electrode for enhanced methane production in microbial electrosynthesis. Energy Convers. Manage. 268, 116018 (2022).
5. Nichols, E. M. et al. Hybrid bioinorganic approach to solar-to-chemical conversion. Proc. Natl Acad. Sci. USA 112, 11461 – 11466 (2015).
6. Li, Q. et al. GO/PEDOT-modified biocathodes promoting CO_2 reduction to CH_4 in microbial electrosynthesis. Sustain. Energy Fuels 4, 2987–2997 (2020).
7. Yang, H. Y. et al. Redox mediator-modified biocathode enables highly efficient microbial electrosynthesis of methane from carbon dioxide. Appl. Energy 274, 115292 (2020).

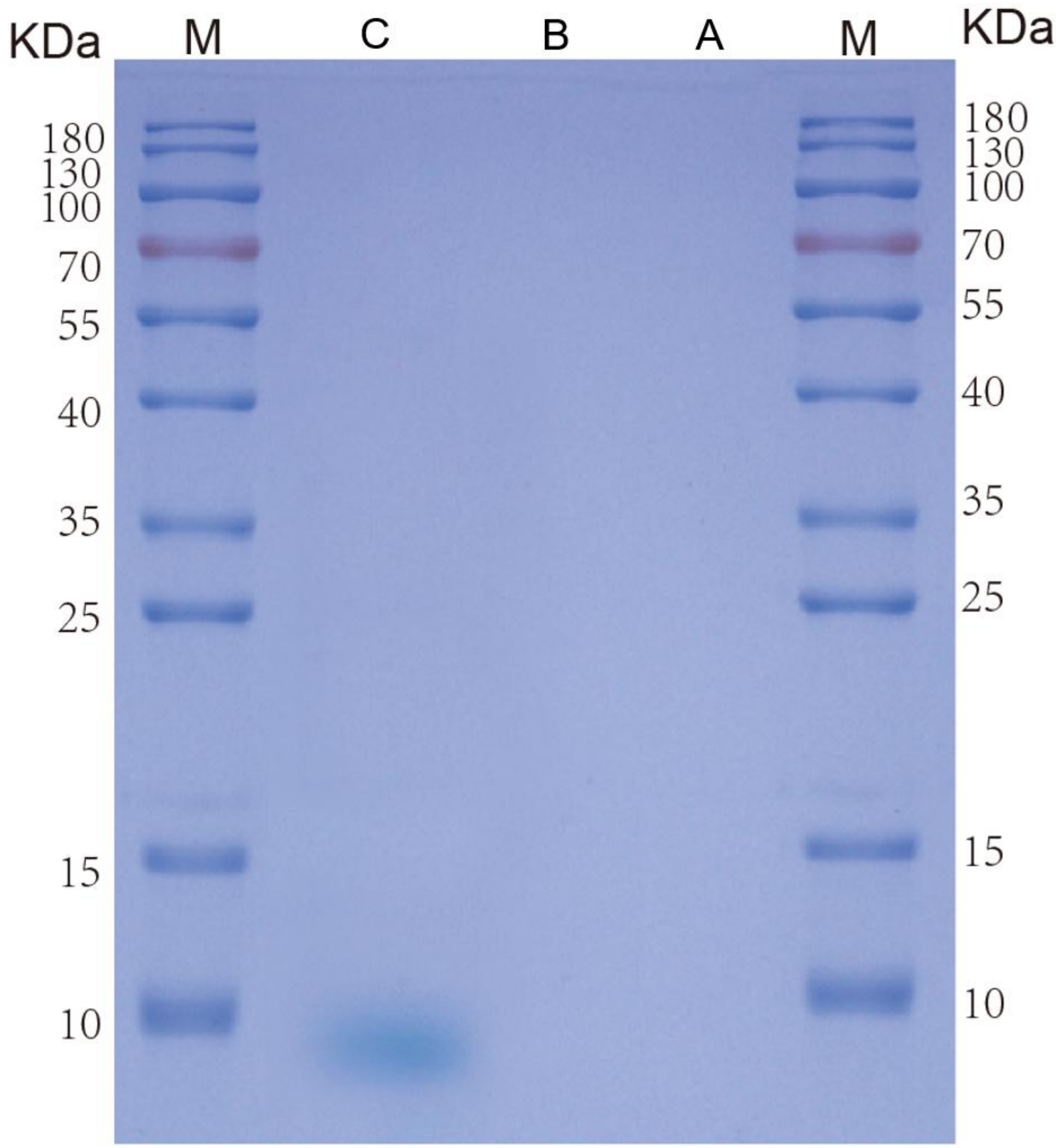

Uncropped gel image corresponding to Supplementary Fig. 10. From left to right, the lanes are M, C, B, A, and M. Lane A, M. b-CF; lane B, M. b-AQC@CF; lane C, M. b-H_2AQC@CF. M, molecular-mass marker. Molecular masses are indicated in kDa. The complete gel borders are shown.
