# Supplementary material for: Quinone-heme π-π interactions bridge the biotic-abiotic interface for enhanced methanogenesis
Source: Nat Commun. 2026 Sep 1;17:10383. doi: 10.1038/s41467-026-77276-8 (PMC13627723; doi:10.1038/s41467-026-77276-8)
Supplement: Supplementary file 2 [file 41467_2026_77276_MOESM2_ESM.pdf]

nature portfolio

Corresponding author(s): Yaobin Zhang

Last updated by author(s): August 12, 2026

# Reporting Summary

Nature Portfolio wishes to improve the reproducibility of the work that we publish. This form provides structure for consistency and transparency in reporting. For further information on Nature Portfolio policies, see our Editorial Policies and the Editorial Policy Checklist.

## Statistics

For all statistical analyses, confirm that the following items are present in the figure legend, table legend, main text, or Methods section.

| n/a | Confirmed | |
|---|---|---|
| ☐ | ☒ | The exact sample size (*n*) for each experimental group/condition, given as a discrete number and unit of measurement |
| ☐ | ☒ | A statement on whether measurements were taken from distinct samples or whether the same sample was measured repeatedly |
| ☒ | ☐ | The statistical test(s) used AND whether they are one- or two-sided<br>*Only common tests should be described solely by name; describe more complex techniques in the Methods section.* |
| ☒ | ☐ | A description of all covariates tested |
| ☒ | ☐ | A description of any assumptions or corrections, such as tests of normality and adjustment for multiple comparisons |
| ☐ | ☒ | A full description of the statistical parameters including central tendency (e.g. means) or other basic estimates (e.g. regression coefficient) AND variation (e.g. standard deviation) or associated estimates of uncertainty (e.g. confidence intervals) |
| ☒ | ☐ | For null hypothesis testing, the test statistic (e.g. *F*, *t*, *r*) with confidence intervals, effect sizes, degrees of freedom and *P* value noted<br>*Give P values as exact values whenever suitable.* |
| ☒ | ☐ | For Bayesian analysis, information on the choice of priors and Markov chain Monte Carlo settings |
| ☒ | ☐ | For hierarchical and complex designs, identification of the appropriate level for tests and full reporting of outcomes |
| ☒ | ☐ | Estimates of effect sizes (e.g. Cohen's *d*, Pearson's *r*), indicating how they were calculated |

*Our web collection on statistics for biologists contains articles on many of the points above.*

## Software and code

Policy information about availability of computer code

| | |
|---|---|
| Data collection | Electrochemical data were collected using a CHI1030C electrochemical workstation (CH Instruments). EPR spectra were acquired using Bruker Xenon software (Bruker). FTIR spectra were analyzed with OMNIC 8.0 (Thermo Fisher Scientific). |
| Data analysis | Data analysis was performed using Origin 2024 (OriginLab, Northampton, MA) for electrochemical data processing and figure preparation. DFT calculations were performed using CP2K (version 2024.1). Input files were generated using Multiwfn (version 3.8) and structures were visualized with VESTA (version 3.5.8). Transcriptomic reads were aligned using Rockhopper (version 2.0.3) and differential expression analysis was conducted using edgeR (version 4.2.0) in R (version 4.3.2). Functional enrichment analysis was performed using Goatools (version 1.4.4) and KOBAS (version 3.0). |

For manuscripts utilizing custom algorithms or software that are central to the research but not yet described in published literature, software must be made available to editors and reviewers. We strongly encourage code deposition in a community repository (e.g. GitHub). See the Nature Portfolio guidelines for submitting code & software for further information.

## Data

Policy information about availability of data

All manuscripts must include a data availability statement. This statement should provide the following information, where applicable:

- Accession codes, unique identifiers, or web links for publicly available datasets
- A description of any restrictions on data availability
- For clinical datasets or third party data, please ensure that the statement adheres to our policy

The raw transcriptomic sequencing data of Methanosarcina barkeri generated in this study have been deposited in the NCBI Sequence Read Archive under BioProject accession code PRJNA1466089. The numerical data underlying the main and supplementary figures generated in this study are provided in the Source Data file. Source data are provided with this paper. No data are subject to access restrictions. All data supporting the findings of the manuscript are available from the corresponding author upon request.

## Research involving human participants, their data, or biological material

Policy information about studies with human participants or human data. See also policy information about sex, gender (identity/presentation), and sexual orientation and race, ethnicity and racism.

| | |
|---|---|
| Reporting on sex and gender | This study did not involve this aspect. |
| Reporting on race, ethnicity, or other socially relevant groupings | This study did not involve this aspect. |
| Population characteristics | This study did not involve this aspect. |
| Recruitment | This study did not involve this aspect. |
| Ethics oversight | This study did not involve this aspect. |

Note that full information on the approval of the study protocol must also be provided in the manuscript.

# Field-specific reporting

Please select the one below that is the best fit for your research. If you are not sure, read the appropriate sections before making your selection.

☐ Life sciences ☐ Behavioural & social sciences ☒ Ecological, evolutionary & environmental sciences

For a reference copy of the document with all sections, see nature.com/documents/nr-reporting-summary-flat.pdf

# Ecological, evolutionary & environmental sciences study design

All studies must disclose on these points even when the disclosure is negative.

| | |
|---|---|
| Study description | The study compared three biohybrid cathode treatments: M. b-CF, M. b-AQC@CF and M. b-$H_2$AQC@CF. Each group contained three independently prepared biohybrid cathodes (nine experimental units in total). For methane-production measurements, cathode potential (−0.8, −0.9, −1.0 and −1.1 V versus Ag/AgCl) was a repeated within-unit factor. Other quantitative endpoints were compared among the three treatments. |
| Research sample | A pure culture of Methanosarcina barkeri DSM 800, obtained from the Leibniz Institute DSMZ, was used as a model methanogenic archaeon. Sex and age are not applicable. The strain was cultivated under strictly anaerobic conditions in a modified medium based on DSMZ Medium 120. The results represent this strain under the tested bioelectrochemical conditions. |
| Sampling strategy | Three independently prepared biohybrid cathodes were analyzed per treatment, with each cathode treated as one biological replicate. No formal sample-size calculation was performed because reliable prior effect-size estimates were unavailable. Three biological replicates were used to evaluate experimental reproducibility; technical measurements were not treated as independent biological replicates. |
| Data collection | Data were collected by the investigators using the instruments and standardized procedures described in the Methods. Methane production, electrochemical responses, biomass, ATP content, EPS characteristics and transcript levels were recorded for the three treatment groups. Identical data-collection procedures were applied across groups. |
| Timing and spatial scale | Data were collected from April to September 2025 at the experimental time points specified in the Methods. All experiments were conducted at the laboratory scale using independently prepared H-type bioelectrochemical reactors. There were no separate sampling cohorts or field sampling locations |
| Data exclusions | No experimental units or collected data were excluded from the analyses. |

Reproducibility: Quantitative experiments were performed using three independently prepared biohybrid cathodes per group. Representative imaging and spectroscopic experiments were independently repeated with similar results. Standardized cultivation, sampling and analytical procedures were used across all replicates.

Randomization: Biohybrid cathodes were assigned to predefined groups according to electrode type and applied potential. Formal randomization was not performed because allocation was determined by the experimental design. Inoculum, medium composition, reactor configuration, cultivation duration and temperature were kept consistent across groups.

Blinding: Investigators were not blinded during data collection or analysis because the groups were identifiable from their electrode materials and applied potentials. The same predefined data-collection and analysis procedures were applied to all groups.

Did the study involve field work? ☐ Yes ☒ No

# Reporting for specific materials, systems and methods

We require information from authors about some types of materials, experimental systems and methods used in many studies. Here, indicate whether each material, system or method listed is relevant to your study. If you are not sure if a list item applies to your research, read the appropriate section before selecting a response.

## Materials & experimental systems

| n/a | Involved in the study | |
|---|---|---|
| ☒ | ☐ | Antibodies |
| ☒ | ☐ | Eukaryotic cell lines |
| ☒ | ☐ | Palaeontology and archaeology |
| ☒ | ☐ | Animals and other organisms |
| ☒ | ☐ | Clinical data |
| ☒ | ☐ | Dual use research of concern |
| ☒ | ☐ | Plants |

## Methods

| n/a | Involved in the study | |
|---|---|---|
| ☒ | ☐ | ChIP-seq |
| ☒ | ☐ | Flow cytometry |
| ☒ | ☐ | MRI-based neuroimaging |

## Plants

Seed stocks: This study did not involve this aspect.

Novel plant genotypes: This study did not involve this aspect.

Authentication: This study did not involve this aspect.
