# Supplementary material for: Quinone-heme π-π interactions bridge the biotic-abiotic interface for enhanced methanogenesis
Source: Nat Commun. 2026 Sep 1;17:10383. doi: 10.1038/s41467-026-77276-8 (PMC13627723; doi:10.1038/s41467-026-77276-8)
Supplement: Supplementary file 3 [file 41467_2026_77276_MOESM3_ESM.pdf]

nature portfolio

Peer Review File

Quinone-heme π-π interactions bridge the biotic-abiotic interface for enhanced methanogenesis

Corresponding Author: Professor Yaobin Zhang

This manuscript has been previously reviewed at another journal. This document only contains information relating to versions considered at Nature Communications.

This file contains all reviewer reports in order by version, followed by all author rebuttals in order by version.

Version 0:

Reviewer comments:

Reviewer #1

(Remarks to the Author)
In the revised manuscript, the authors rescind their original claim of π-π interaction from " direct protein interaction" to a Heme-mediated relay" model, which operates across a markedly thinned EPS layer (methanochondroitin layer, but without any direct evidence.

They propose a new hypothesis that "quinone moieties specifically capture and immobilize free heme prosthetic groups within the EPS via π-π interactions. This interaction stabilizes the heme structure, maintaining its redox activity and preventing it from diffusing away, which effectively enhances the electroactivity of the EPS."

Again, no evidence is presented to support this hypothesis. This new hypothesis is based on the assumption that Methanosarcina barkeri secretes free heme prosthetic groups that become trapped in EPS via interaction with quinone. However, none of the presented data supports this model. First, free heme prosthetic groups cannot exist without the surrounding protein due to their highly hydrophobic nature, which will cause them to aggregate. Therefore, all cytochrome hemes are embedded in proteins. To my knowledge, M. barkeri are shown to secrete free heme prosthetic groups. Hemes in cytochrome b or cytochrome c rarely exist as free hemes and require detailed protein architecture surrounding them to occlude their hydrophobic surface and control their redox potential, none of which is possible for free hemes.

The authors do not show that M. barkeri can secrete free heme prosthetic groups.
Raman spectra show several bands, none of which are specific to hemes. Transcriptomic data cannot show the protein abundance or the extracellular localization. Without direct evidence that M. barkeri secretes free heme prosthetic groups that localize in the EPS, and quinones secreted bind to these hemes and stabilize the structure, the entire model is not justified and has no biological relevance.

The authors can evaluate this directly by performing cellular localization studies and using heme staining and mass-spectrometry to evaluate the interaction via UV-Vis or EPR spectroscopy with detailed model fitting. Instead, the presented data rely on g-values, which are not easily interpretable. Furthermore, if quinone is indeed transferring electrons to hemes, the authors should not have seen any of the Fe(II) signal in their EPR, as the reduced hemes are silent in EPR and cannot be detected and require Mössbauer spectroscopy.

Instead of including experiments that evaluate the direct presence of free hemes in EPS and the underlying secretion system, the authors respond that “while M. barkeri is generally considered to lack surface-exposed cytochromes, the existence of unidentified surface proteins, similar to the c-type cytochromes found in Methanosarcina acetivorans, cannot be entirely excluded.”

This argument, presented by the author that “absence of evidence is not evidence of absence,” cannot be used to justify the central claims of the manuscript. Without evidence for a secretion system for free heme prosthetic groups that are localized in EPS, the claims are not justified.

Reviewer #2

(Remarks to the Author)
I served as Reviewer 2 in the previous review process. The conclusions of this study are well supported by the experimental data, and the revisions made in response to the earlier comments are reasonable. However, as I noted in my previous review, the proposed electron transfer mechanism between quinones and hemes was demonstrated using model compounds rather than cytochromes, which leaves open the question of whether such a mechanism actually occurs in the electron transfers in biotic–abiotic interfaces, let alone whether it functions within extracellular polymeric substances (EPS). While I do not question the validity of the conclusions of this study, I remain concerned that the work may lack the broader appeal required for publication in this journal.

Reviewer #3

(Remarks to the Author)
Overall, the responses to my comments are largely accurate, and the revised manuscript is more internally consistent in both data interpretation and mechanistic framing. In particular, the mechanistic discussion of a heme-mediated relay at the bio–abiotic interface is more plausible than the original direct contact mechanism. I have two final suggestions.
- Please add a concise paragraph that states the engineering design principle derived from this work and how it can be generalized beyond the specific conditions tested here.
- Please expand your outlook to describe practical implications and potential applications in anaerobic digesters and/or other engineered environments, including key considerations for implementation and scalability.

Version 1:

Reviewer comments:

Reviewer #1

(Remarks to the Author)
In the rebuttal, the authors claim to show direct evidence of heme-b transporting electrons in EPS. I have the following concerns about their claims.

1) Lack of distinction between heme-b vs. heme-c. The presented data suggested the presence of heme-c rather than heme-b. The beta band at 524 nm is more pronounced, which is a hallmark of heme-c due to the covalent thioether bonds restricting the heme. Similarly, the alpha band (556 nm) is well resolved as expected for heme-c, whereas heme-b shows a broader alpha band. Neither CV nor heme-staining shows that the observed data are specific to heme-b and not due to heme-b from intracellular cytochromes. The molecular weight of free heme is 616 Da, which is ~0.5 kDa. The authors observe signals just below 10 kDa, which is consistent with the signal of most periplasmic cytochrome c. The lack of signal unique to 0.5 kDa further indicates that there are no free heme-b in the EPS. The CV data further indicates heme-c due to the absence of distinct redox peaks expected for free hemes. Basic analysis of the first derivative needs to be performed for CV and EPR spectra, which need to be modeled for high-spin hemes, as free cyt-b should lack ligands and must be high spin. The lack of high-spin features further indicates the lack of cyt-b in the EPS.

2) Lack of evidence for heme relay. Heme-c cannot participate in electron relay because it is covered by protein and requires a near c-c distance (~ 3.5 A) for π-stacking that could enable efficient electron transfer. Even for heme-b, the porphyrin ring needs to be π-stacked (~ 3.5 A). The authors do not show that hemes are indeed spaced at such a stacking distance that can facilitate electron relay. X-ray diffraction of EPS should reveal any π-stacking present.

3) Lack of physiological evidence of cells transferring electrons via EPS. The data presented suggest some electrochemical activity between the extracted EPS and the electrode, but that does not mean that cells are transferring electrons via EPS. Direct evidence of cellular electron transfer via EPS is needed through electrical measurements across the living cells using interdigitated electrodes and not from EPS to the electrode. See e.g. (Xu et al. JACS, 2018 140 (32), 10085-10089. DOI: 10.1021/jacs.8b05104)

4) Physiological studies contradict the proposed model. A recent paper published in the same journal shows that Methanosarcina cannot exchange electrons in the absence of conductive particles externally added to cells (Nature Communications 17, 2708, 2026). This requirement for conductive particles contradicts the proposed model that cells can use EPS to transfer electrons.

Version 2:

Reviewer comments:

Reviewer #4

(Remarks to the Author)
Overall, this manuscript presents solid research with distinct and innovative research insights, and I think the authors have

provided adequate replies to the comments raised by Reviewer 1. From my perspective, a few evaluation criteria proposed by Reviewer 1 may be relatively high considering the research scope of this work and the inherent technical limitations associated with the target archaeon. My detailed comments are listed below:
1. Identification of heme-b and heme-c. Reviewer 1 proposed that the measured signals tend to match heme-c or intracellular heme-b rather than the labile heme-b described in the original text. The authors supplemented genomic analysis (M. barkeri does not possess an intact functional Ccm maturation pathway, which restricts endogenous heme-c synthesis) and newly acquired UV-Vis spectra for comparative analysis between EPS samples and heme-c reference standards, which provides reliable support for the assignment of labile heme-b. The rebuttal information provided by the authors can well address this concern.
2. π-π stacking distance relevant to electron transfer pathways. Reviewer 1 pointed out that the study lacked data supporting the ~3.5 Å interlayer distance required for heme-heme π-π stacking. This confusion may arise from the comparison with Geobacter nanowires mentioned in the initial manuscript. The authors have deleted this comparative content and clarified that the π-π interaction occurs between quinone and heme groups instead of adjacent heme molecules, which is further backed by the amorphous XRD results. The revised statement is logical and can resolve this question.
3. Direct physiological characterization based on interdigitated electrode testing. Reviewer 1 suggested conducting transcellular conductivity tests to verify extracellular electron transfer through EPS by intact viable cells. However, such measurements face considerable technical challenges for M. barkeri: this obligate anaerobic archaeon has a delicate S-layer surface structure and forms loose cell aggregates instead of tightly attached single layers, so it is hard to complete electrode assembly without causing cell damage. Under such experimental limitations, the multiple in-situ characterization results provided by the authors (SECM, CV curves, coupled variation trend of methane yield and electric current) can serve as acceptable alternative evidence.
4. Inconsistency with existing literature regarding conductive additives. Reviewer 1 referenced a recent publication (Nat. Commun. 17, 2708, 2026) reporting that Methanosarcina strains rely on exogenous conductive particles to carry out extracellular electron exchange. The authors explained that the microbial consortium in the cited paper was long-term domesticated with GAC over ten years, while the current work adopted undomesticated pure cultures. Relevant published results have indicated that M. barkeri can receive electrons from cathode surfaces without conductive mediators, which aligns with the mechanism proposed in this manuscript. Therefore, the two studies do not present obvious conflicting conclusions.

In summary, the authors supplemented new experimental data and refined research conclusions to sufficiently respond to comment 1 and comment 2. The requirements put forward in comment 3 and comment 4 are difficult to achieve under current experimental conditions and slightly exceed the research objectives of this work.

My specific comments that should be required for revision are as follows:
1. The role of HdrDE in the proposed mechanism requires clarification. The authors describe HdrDE as the source of heme (via lysis), the functional response to the established π-π channel (upregulation), and the proton pump driving ATP enhancement. However, the proposed mechanism is essentially a physicochemical event within the EPS and does not necessarily depend on HdrDE. The upregulation of HdrDE could simply reflect a feedback response to the increased downstream methanogenic flux rather than direct induction by the π-π interaction, and heme could equally originate from other cytochromes upon lysis. In addition, the proton-pumping function of HdrDE in Methanosarcina remains debated in the literature, and more cautious wording is recommended.
2. The contribution of H_2 evolution remains unaddressed, as H_2 production can occur below -0.8 V. Moreover, the reported faradaic efficiency of 85.8% applies only to the optimal condition; the fate of the remaining ~14% of electrons should be clarified.
3. In fig 3, both control groups exhibit weak and broad absorption features around 409 nm rather than a flat baseline, yet the main text describes them as "no such feature was observed in either control group". This should be clarified.
4. The identification of heme b relies solely on comparison with a heme c standard (cytochrome c), with α/β band differences of only 4-6 nm. A heme b standard should be included as a positive control to validate the assignment.
5. Several methodological details require clarification: (1) The "fresh medium" is not specified, including whether it still contains cysteine, methanol, or other potential electron donors. (2) The EIS methodology is absent from the SI, and the equivalent circuit should be justified. (3) The normalization basis for the ATP and qPCR data is not specified (per mg protein? per cm^2 electrode area?).

Version 3:

Reviewer comments:

Reviewer #4

(Remarks to the Author)
The authors have carefully addressed all the points raised in my prior review and implemented comprehensive revisions to the manuscript. Based on the updated version, I support the acceptance of this work for publication.

Open Access This Peer Review File is licensed under a Creative Commons Attribution 4.0 International License, which permits use, sharing, adaptation, distribution and reproduction in any medium or format, as long as you give appropriate credit to the original author(s) and the source, provide a link to the Creative Commons license, and indicate if changes were made.
In cases where reviewers are anonymous, credit should be given to 'Anonymous Referee' and the source.
The images or other third party material in this Peer Review File are included in the article's Creative Commons license, unless indicated otherwise in a credit line to the material. If material is not included in the article's Creative Commons license and your intended use is not permitted by statutory regulation or exceeds the permitted use, you will need to obtain permission directly from the copyright holder.
To view a copy of this license, visit https://creativecommons.org/licenses/by/4.0/

Response letter to Reviewers and Editor

Dear Editor and Reviewers:

We greatly appreciated all the helpful comments from the Editor and Reviewers on the manuscript NCOMMS-25-103702-T (Entitled: Quinone-Heme π-π interactions bridge the biotic-abiotic interface for enhanced methanogenesis). According to these comments, we have revised the manuscript carefully as follows:

Black words represented the comments from the Editor and Reviewers.
Blue words represented the responses from the Authors.
Red words represented the emphatic responses or revisions in the manuscript from the Authors.

Reviewer #1's Comments

In the revised manuscript, the authors rescind their original claim of π-π interaction from " direct protein interaction" to a Heme-mediated relay" model, which operates across a markedly thinned EPS layer (methanochondroitin layer, but without any direct evidence.

They propose a new hypothesis that "quinone moieties specifically capture and immobilize free heme prosthetic groups within the EPS via π-π interactions. This interaction stabilizes the heme structure, maintaining its redox activity and preventing it from diffusing away, which effectively enhances the electroactivity of the EPS."

Again, no evidence is presented to support this hypothesis. This new hypothesis is based on the assumption that Methanosarcina barkeri secretes free heme prosthetic groups that become trapped in EPS via interaction with quinone. However, none of the presented data supports this model. First, free heme prosthetic groups cannot exist without the surrounding protein due to their highly hydrophobic nature, which will cause them to aggregate. Therefore, all cytochrome hemes are embedded in proteins. To my knowledge, M. barkeri are shown to secrete free heme prosthetic groups. Hemes in cytochrome b or cytochrome c rarely exist as free hemes and require detailed protein

architecture surrounding them to occlude their hydrophobic surface and control their redox potential, none of which is possible for free hemes.

The authors do not show that M. barkeri can secrete free heme prosthetic groups.

Raman spectra show several bands, none of which are specific to hemes. Transcriptomic data cannot show the protein abundance or the extracellular localization. Without direct evidence that M. barkeri secretes free heme prosthetic groups that localize in the EPS, and quinones secreted bind to these hemes and stabilize the structure, the entire model is not justified and has no biological relevance.

The authors can evaluate this directly by performing cellular localization studies and using heme staining and mass-spectrometry to evaluate the interaction via UV-Vis or EPR spectroscopy with detailed model fitting. Instead, the presented data rely on g-values, which are not easily interpretable. Furthermore, if quinone is indeed transferring electrons to hemes, the authors should not have seen any of the Fe(II) signal in their EPR, as the reduced hemes are silent in EPR and cannot be detected and require Mössbauer spectroscopy.

Instead of including experiments that evaluate the direct presence of free hemes in EPS and the underlying secretion system, the authors respond that "while M. barkeri is generally considered to lack surface-exposed cytochromes, the existence of unidentified surface proteins, similar to the c-type cytochromes found in Methanosarcina acetivorans, cannot be entirely excluded."

This argument, presented by the author that "absence of evidence is not evidence of absence," cannot be used to justify the central claims of the manuscript. Without evidence for a secretion system for free heme prosthetic groups that are localized in EPS, the claims are not justified.

Answer：

Thank you for your continued and thorough engagement with our manuscript, and for the professional and constructive comments provided. Each point raised has been carefully considered, and the responses have been substantially revised accordingly. It is noted that the term "secretes" was referenced in describing the proposed mechanism, which likely reflects an ambiguity in the original text. To clarify the model at the outset:

the proposed framework does not invoke any active secretion mechanism. Specifically: (1) heme is proposed to originate from cell death and lysis, a normal physiological process within the microbial community, whereby proteins containing non-covalently bound heme b are disrupted, allowing their prosthetic groups to passively enter the EPS; (2) quinone moieties are immobilized on the electrode surface and are never secreted. Instead, they capture and stabilize the heme that migrates to the electrode-EPS interface via π-π interactions, forming a redox relay. The manuscript has been revised to state this mechanism explicitly and unambiguously. Direct experimental evidence is provided below to address each specific concern in detail.

1. Identification of labile, electrochemically active heme b in the EPS

Answer：

Thank you for raising this important concern. To directly confirm the presence of heme b in the isolated EPS fraction, three independent lines of experimental evidence were obtained: UV-Vis, LDS-PAGE, and CV.

UV-Vis Spectroscopy: A sharp Soret band at 409 nm was observed exclusively in the EPS of M. b-H_2AQC@CF group. Consistent with monomeric Fe^{3+} heme in aqueous solution **(Simianu et al., 1998).** Upon chemical reduction with sodium dithionite (pyridine hemochrome assay), characteristic α/β bands appeared at 524 nm and 556 nm, representing the classical Q-band signatures of Fe^{2+} heme b **(Berry & Trumpower, 1987).** Together, these features constitute a complete Fe^{3+}/Fe^{2+} redox-coupled spectral fingerprint with high specificity for heme b.

LDS-PAGE Heme-Specific Staining: A positive signal in the low molecular weight region (below 10 kDa) was revealed in the M.b-H_2AQC@CF group, while both control groups were negative. This migration behavior is consistent with either free heme b or small peptide-associated heme b released following cell lysis. In either case, the signal confirms the presence of labile heme b within the EPS, distinct from any intact hemoprotein which would migrate at substantially higher molecular weight **(Heiden et al., 1993).**

Cyclic Voltammetry (CV): A reversible redox peak in the potential range of -0.15 V

to -0.25 V (vs. Ag/AgCl) was observed in the EPS of the M. b-H_2AQC@CF, independently confirming the presence of electrochemically accessible Fe^{3+}/Fe^{2+} heme centers. This redox potential closely matched that of the electroactive heme cofactor in heme b **(San Francisco et al., 2011; Reedy & Gibney, 2004).**

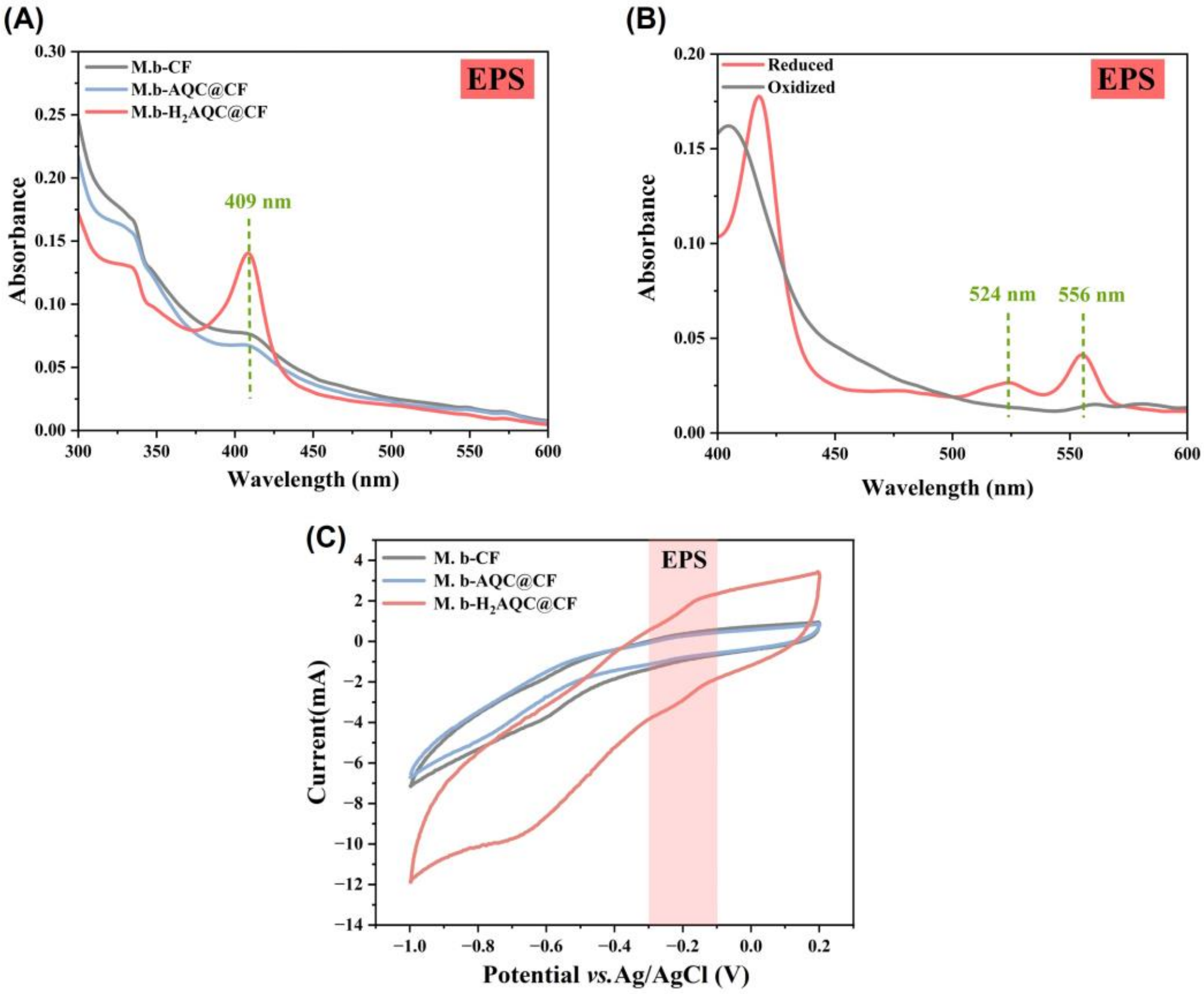

Characterization of electrochemically active heme b in EPS. UV-Vis absorption spectra of the EPS of the three biohybrids (A), UV-Vis spectra of M.b-H_2AQC@CF EPS in the oxidized and chemically reduced states (B), Cyclic voltammograms of the EPS (C).

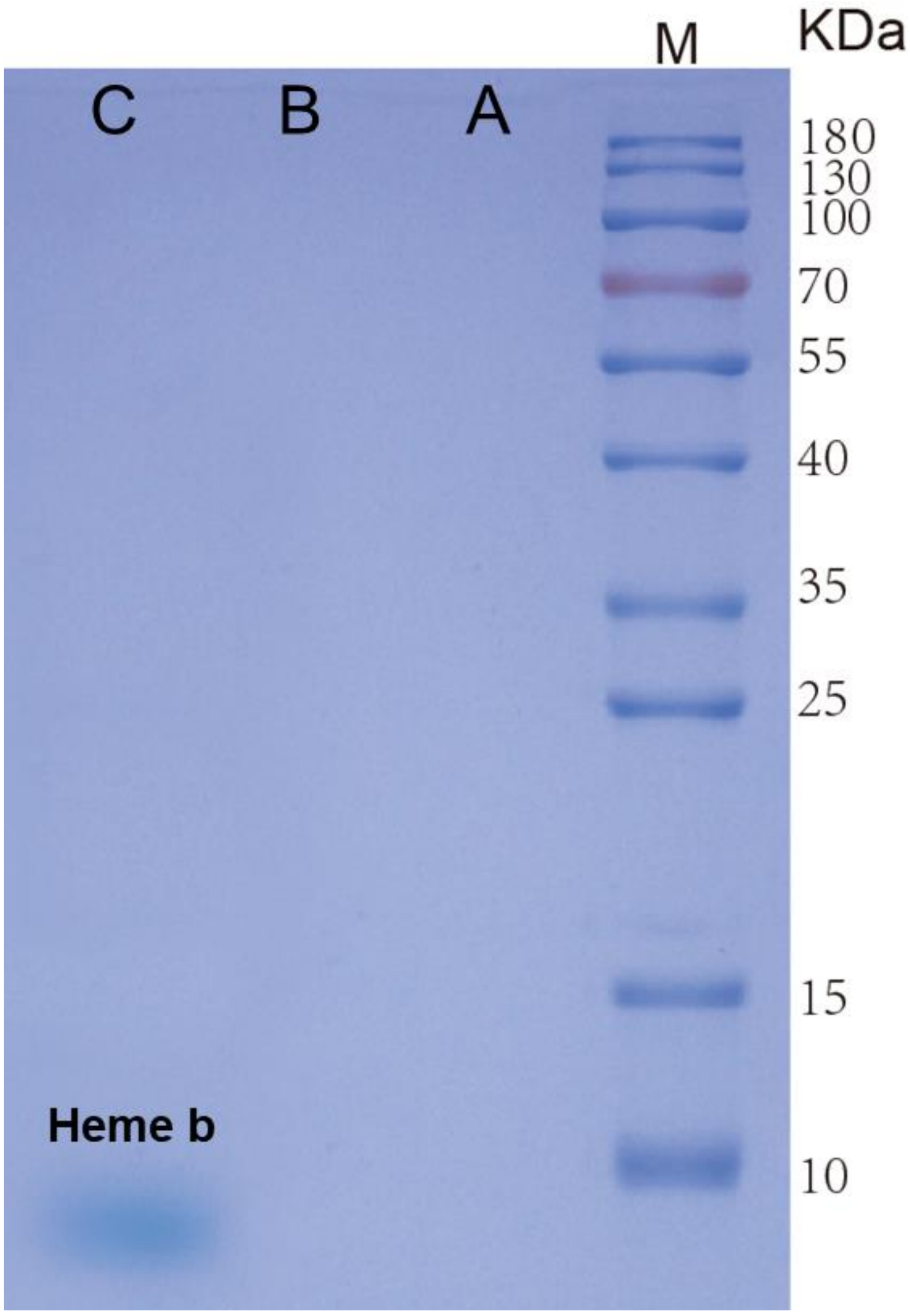

LDS-PAGE heme-specific staining of EPS from biohybrid groups. (lane A: M.b-CF; lane B: M.b-AQC@CF; lane C: M.b-H_2AQC@CF).

The following text has been added to the manuscript: To directly confirm this, multiple independent lines of evidence were obtained. As shown in Fig 3E, UV-Vis spectroscopy revealed a sharp Soret band at 409 nm exclusively in the EPS of the M. b-H_2AQC@CF group, consistent with monomeric ferric (Fe^{3+}) heme[38], while no such feature was observed in either control group. Upon treatment with pyridine and chemical reduction (pyridine hemochrome assay), characteristic α/β bands appeared at 524 nm and 556 nm (Fig 3F), representing the classical pyridine hemochrome signatures of Fe^{2+} heme[39]. Together, these features constitute a complete Fe^{3+}/Fe^{2+} redox-coupled spectral fingerprint with high specificity for heme. Further corroborating evidence was provided by heme-specific LDS-PAGE staining, which revealed a positive signal in the low molecular weight region exclusively in the M. b-H_2AQC@CF group, consistent with labile heme released following cell lysis, while both control groups were negative (Fig S8) [40]. CV analysis further revealed that the EPS from M. b-H_2AQC@CF exhibited a pair of clear and reversible redox peaks in the potential range of -0.15 V to -0.25 V (Fig 3D), closely matching the characteristic redox potential of electroactive heme cofactors

reported in the literature[41,42]. Together, these results directly confirm the presence of redox-active heme within the EPS of the M. b-H_2AQC@CF biohybrid.

Reference:

Simianu, M.; Murakami, E.; Brewer, J. M.; Ragsdale, S. W. Purification and Properties of the Heme- and Iron-Sulfur-Containing Heterodisulfide Reductase from Methanosarcina thermophila. *Biochemistry* 1998, 37 (28), 10027-10039. DOI: 10.1021/bi9726483

Berry, E. A.; Trumpower, B. L. Simultaneous Determination of Hemes a, b, and c from Pyridine Hemochrome Spectra. *Anal. Biochem.* 1987, 161 (1), 1-15. DOI: 10.1016/0003-2697(87)90643-9

Heiden, S.; Hedderich, R.; Setzke, E.; Thauer, R. K. Purification of a cytochrome b containing H_2: heterodisulfide oxidoreductase complex from membranes of Methanosarcina barkeri. *Eur. J. Biochem. 1993*, 213 , 529-535.

San Francisco, B.; Bretsnyder, E. C.; Rodgers, K. R.; Kranz, R. G. Heme Ligand Identification and Redox Properties of the Cytochrome c Synthetase, CcmF. *Biochemistry* 2011, 50, 10974-10985. DOI: 10.1021/bi201508t

Reedy, C. J.; Gibney, B. R. Heme Protein Assemblies. *Chem. Rev.* 2004, 104 (2), 617-649. DOI: 10.1021/cr0206115

2. Stability of labile heme b in EPS (no self-aggregation).

Answer:

Given that self-aggregated heme characteristically lacks electrochemical activity, the sharp Soret band at 409 nm and the reversible CV redox peak directly demonstrate that heme b in the EPS maintains a non-aggregated, active state. This can likely be attributed to the aromatic π interface of electrode, which inhibits the auto-dimerization of heme in aqueous solutions via π-π stacking interactions, thereby retaining its catalytic and electrochemical activities. Such stabilization mechanisms have been well-documented in previous literature **(Xue et al., 2012; Chen et al., 2011)**. In the present study, the π-π interactions between the H_2AQC interface and heme are significantly stronger and more specific. DFT calculations reveal a net charge transfer of 0.158 electrons at the H_2AQC interface, compared to merely 0.022 electrons for the bare carbon felt. This substantial difference underscores the pronounced advantage of H_2AQC over bare carbon interfaces in stabilizing heme. Furthermore, unlike a pure aqueous environment, the complex polymeric EPS matrix (comprising polysaccharides, proteins, and nucleic acids) creates a distinct physicochemical microenvironment that likely also influence the aggregation of heme b.

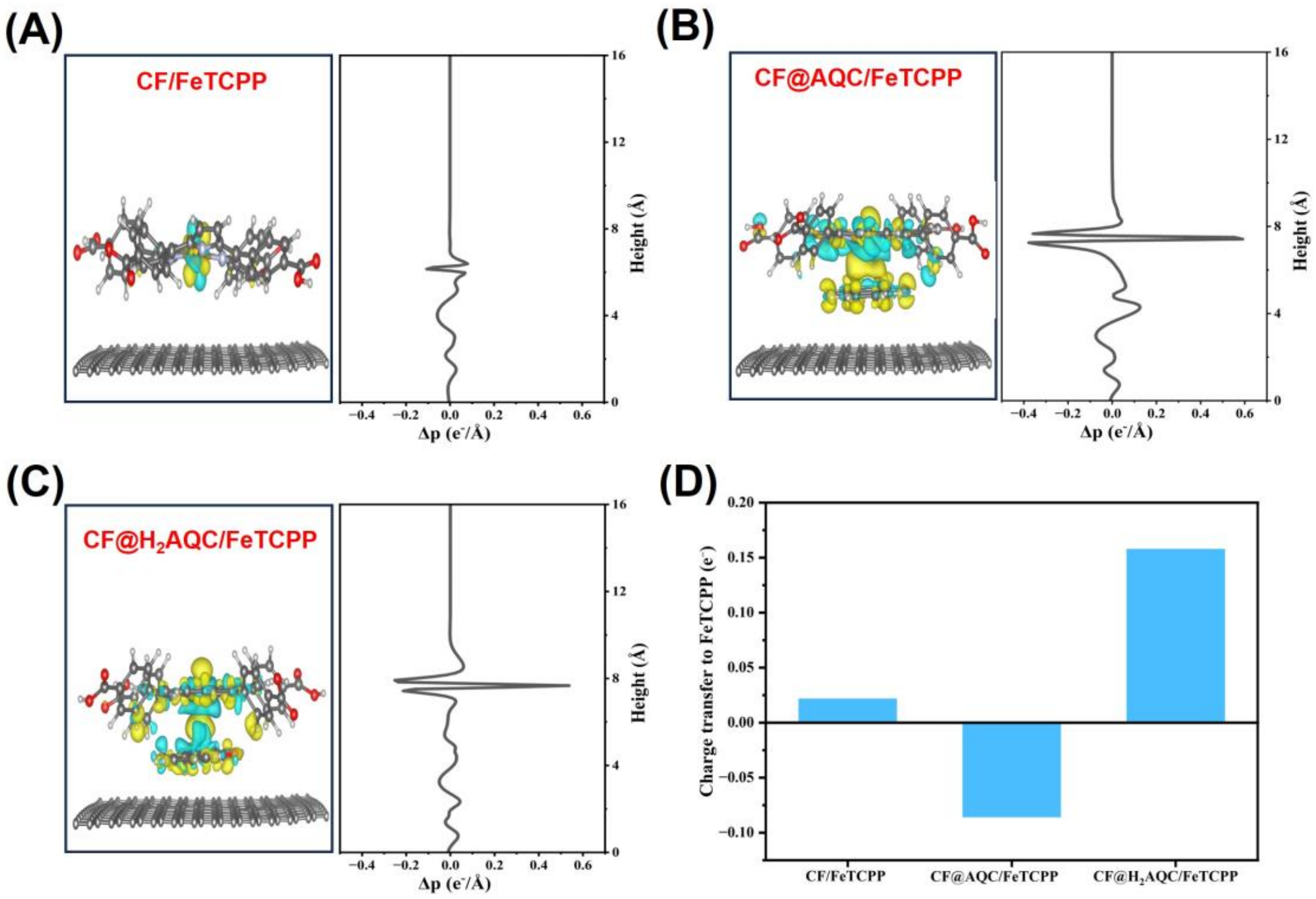

Density Functional Theory (DFT) analysis of the interfacial interactions between three molecular interfaces and FeTCPP. Calculated stable configurations and corresponding Charge Density Difference (CDD) plots for the CF/FeTCPP (A), CF@AQC/FeTCPP (B), and CF@H_2AQC/FeTCPP (C), respectively. Comparison of the net charge transferred to the FeTCPP molecule in the three systems, calculated using the CM5 model (D).

The following text has been added to the manuscript: Labile heme released into aqueous environments is typically prone to self-aggregation, forming electrochemically inactive aggregates. This expectation is consistent with the two control groups where no electrochemically active heme was detected. The sharp Soret band at 409 nm and the reversible CV redox peak observed exclusively in the M. b-H_2AQC@CF EPS confirm that heme maintains a non-aggregated, active state, likely attributable to the H_2AQC interface competitively inhibiting heme auto-dimerization via preferential π-π stacking. Such stabilization mechanisms have been documented for porphyrin immobilization on aromatic carbon interfaces[60,61]. DFT calculations further confirm that the π-π interactions at the H_2AQC interface are significantly stronger than those of bare carbon surfaces, underscoring the specific advantage of quinone functionalization.

Reference：

Xue, T.; Jiang, S.; Qu, Y.; Su, Q.; Cheng, R.; Dubin, S.; Chiu, C.-Y.; Kaner, R.; Huang, Y.; Duan, X. Graphene-Supported Hemin as a Highly Active Biomimetic Oxidation Catalyst. *Angew. Chem., Int. Ed.* 2012, 51 (16), 3822-3825. DOI: 10.1002/anie.201108400

Chen, J.; Zhao, L.; Bai, H.; Shi, G. Electrochemical Detection of Dioxygen and Hydrogen Peroxide by Hemin Immobilized on Chemically Converted Graphene. *J. Electroanal. Chem.* 2011, 657 (1-2), 34-38. DOI: 10.1016/j.jelechem.2011.03.005

3. Evidence for π-π interaction between quinone and heme b

Answer：

To validate the proposed quinone-heme interaction, UV-Vis spectroscopic experiments were conducted. Pure AQC (oxidized) and H_2AQC (reduced) exhibit absorption peaks at 339 nm and 407 nm, respectively. Mixing AQC with EPS (M.b-H_2AQC@CF) maintained the 339 nm peak, indicating that the EPS matrix does not inherently perturb AQC absorption. In contrast, introducing H_2AQC to EPS (M.b-H_2AQC@CF) triggered a redox reaction where H_2AQC was oxidized to AQC by Fe^{3+} heme b, resulting in an AQC peak at 334 nm. This 5 nm blue shift indicates that H_2AQC initially forms a π-π complex with heme b through electronic overlap, which allows the resulting AQC to remain "locked" in the interaction site and preserve the π-π stacked configuration. Critically, the observed 5 nm blue shift is consistent with H-type excitonic coupling arising from this face-to-face π-π interaction (DFT analysis), wherein antiparallel alignment of transition dipoles elevates the allowed excited-state energy **(Kasha.,1963; Hestand & Spano., 2018)**.

This conclusion is further corroborated by two mutually consistent lines of evidence: (i) broadening of the C=C stretching band in biomimetic Raman spectra, indicating restricted molecular vibration upon π-π complex formation; (ii) the emergence of a C 1s XPS satellite peak at 290.30 eV, a recognized fingerprint of π-π interactions, which is absent in controls

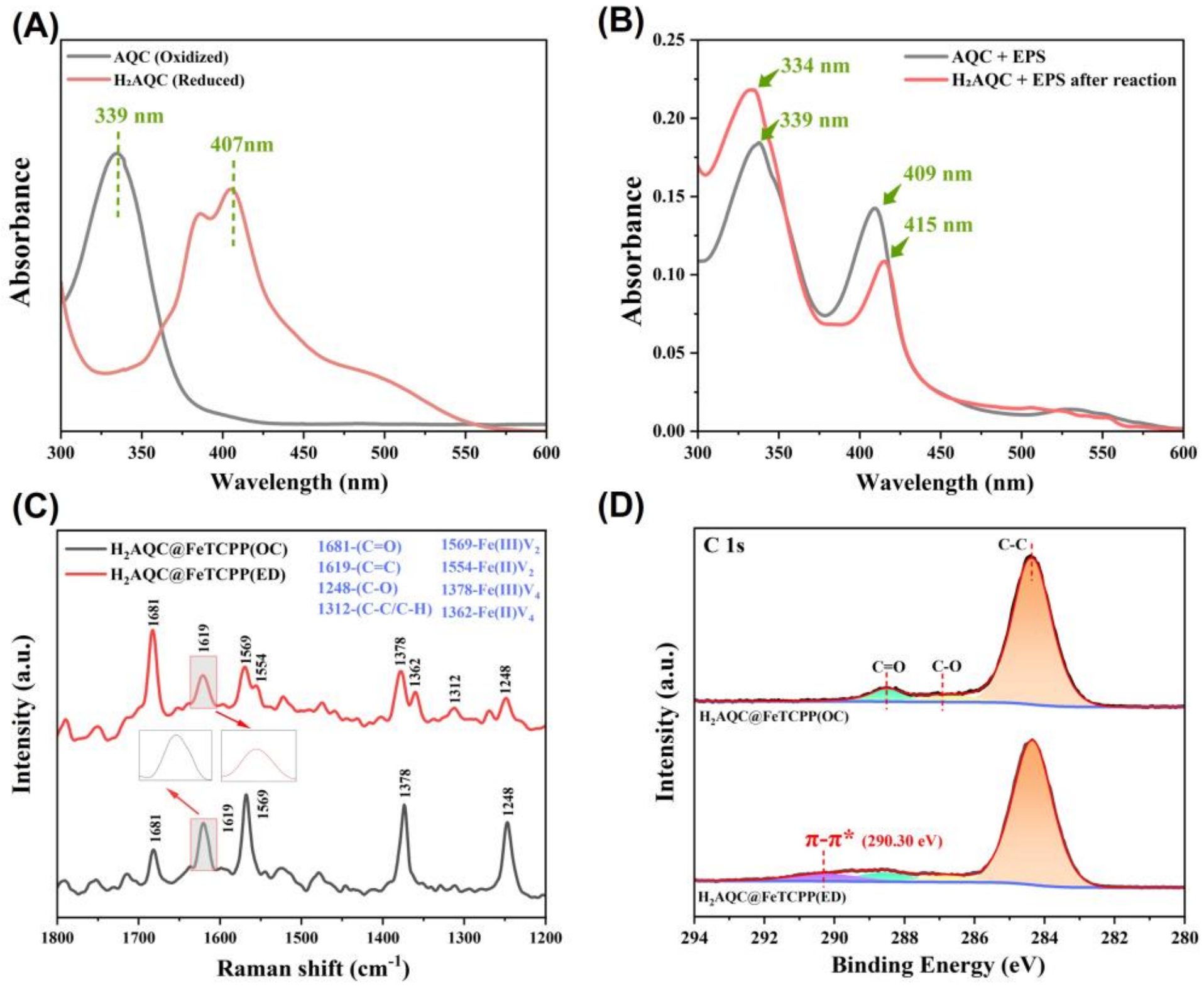

Spectroscopic evidence for quinone-heme π-π interactions. UV-Vis spectra of pure AQC (339 nm) and H_2AQC (407 nm) (A). UV-Vis spectra of AQC + EPS (M. b-H_2AQC@CF) and H_2AQC + EPS (M. b-H_2AQC@CF) after reaction (B). Raman (C) and XPS C1s spectra (D) of H_2AQC@FeTCPP under open-circuit (OC) and electrodeposition (ED)

The following text has been added to the manuscript: To validate the proposed quinone-heme interaction, UV-Vis experiments were conducted. Pure AQC (oxidized) and H_2AQC (reduced) exhibit absorption peaks at 339 nm and 407 nm, respectively (Fig S12). Mixing AQC with EPS (M. b-H_2AQC@CF) maintained the 339 nm peak, indicating that the EPS matrix does not inherently perturb AQC absorption. In contrast, introducing H_2AQC to EPS (M. b-H_2AQC@CF) triggered a redox reaction where H_2AQC was oxidized to AQC by Fe^{3+} heme b, resulting in an AQC peak at 334 nm (Fig 4C). This 5 nm blue shift indicates that H_2AQC initially forms a π-π complex with heme b through electronic overlap, which allows the resulting AQC to remain locked in the interaction site and preserve the π-π stacked configuration[49,50].

Reference：

Kasha, M. Energy transfer mechanisms and the molecular exciton model for molecular aggregates. *Radiat. Res.* 1963, 20, 55-70. DOI: 10.2307/3571331

Hestand, N. J.; Spano, F. C. Expanded Theory of H- and J-Molecular Aggregates: The Effects of Vibronic Coupling and Intermolecular Charge Transfer. *Chem. Rev.* 2018, 118 (15), 7069-7163. DOI: 10.1021/acs.chemrev.8b00244

4. Biological plausibility of heme b in EPS

Answer：

The proposed model is grounded in a well-documented physiological process, specifically cell death and lysis, which serves as a consistent source of membrane-associated components within the EPS matrix **(Turnbull et al., 2016; Flemming & Wingender, 2010).** *Methanosarcina barkeri* is known to contain extractable, non-covalently bound heme b **(Bröcker et al., 2006).** Unlike covalently bound c-type cytochrome hemes that resist release upon protein denaturation or cell disruption, non-covalently bound heme b can readily dissociate from its protein scaffold, as established in the context of hemolytic heme release **(Gouveia et al., 2017)**, and thereby enter the EPS matrix as labile heme b. Importantly, extracellular heme b has been directly detected in the culture milieu of *M. barkeri*, providing direct precedent for the presence of labile heme b in the extracellular environment **(Lin et al., 1989; 1990).**

As correctly pointed out by you, labile heme released into an aqueous environment is typically expected to self-aggregate or degrade, thereby losing its electrochemical activity. This expectation is entirely consistent with the two control groups (M.b-CF and M.b-AQC@CF) where no electrochemically active heme b was detected. However, H_2AQC interface specifically stabilizes labile heme b in the M.b-H_2AQC@CF system through π-π interactions. This stabilization effectively prevents aggregation and preserves the redox activity of the heme, as detailed in Point 2.

The following text has been added to the manuscript: Cell death and lysis within microbial communities serve as a consistent source of membrane-associated components within the EPS matrix[55,56]. Unlike covalently bound c-type cytochrome hemes, non-covalently bound heme b can readily dissociate from its protein scaffold upon cell disruption[57], entering the EPS as labile heme. This is supported by direct detection of extracellular heme in *M. barkeri* cultures[58,59]. Of particular relevance, the HdrE subunit, which was specifically upregulated in the M. b-H_2AQC@CF biohybrid, harbors two heme b cofactors[18], representing a likely primary source of labile heme released into the EPS. Rather than representing a loss of cellular function, this physiological turnover constitutes a sustained and renewable supply of interfacial redox mediators.

Reference：

Turnbull, L.; Toyofuku, M.; Hynen, A. L.; Kurosawa, M.; Pessi, G.; Petty, N. K.; Osvath, S. R.; Cárcamo-Oyarce, G.; Gloag, E. S.; Shimoni, R.; Omasits, U.; Ito, S.; Yap, X.; Monahan, L. G.; Cavaliere, R.; Ahrens, C. H.; Charles, I. G.; Nomura, N.; Eberl, L.; Whitchurch, C. B. Explosive Cell Lysis as a Mechanism for the Biogenesis of Bacterial Membrane Vesicles and Biofilms. *Nat. Commun*. 2016, 7, 11220. DOI: 10.1038/ncomms11220

Flemming, H.-C.; Wingender, J. The Biofilm Matrix. *Nat. Rev. Microbiol*. 2010, 8 (9), 623–633. DOI: 10.1038/nrmicro2415

Buchenau, B.; Kahnt, J.; Heinemann, I. U.; Jahn, D.; Thauer, R. K. Heme Biosynthesis in Methanosarcina barkeri via a Pathway Involving Two Methylation Reactions. *J. Bacteriol*. 2006, 188 (24), 8666–8668. DOI: 10.1128/JB.01349-06

Gouveia, Z.; Carlos, A. R.; Yuan, X.; Aires-da-Silva, F.; Stocker, R.; Maghzal, G. J.; Leal, S. S.; Gomes, C. M.; Todorovic, S.; Iranzo, O.; Ramos, S.; Santos, A. C.; Hamza, I.; Gonçalves, J.; Soares, M. P. Characterization of Plasma Labile Heme in Hemolytic Conditions. *FEBS J*. 2017, 284 (19), 3278–3301. DOI: 10.1111/febs.14192

Lin, D.; Nishio, N.; Mazumder, T. K.; Nagai, S. Influence of Co^{2+}, Ni^{2+} and Fe^{2+} on the Production of Tetrapyrroles by Methanosarcina barkeri. *Appl. Microbiol. Biotechnol*. 1989, 30, 196–200. DOI: 10.1007/BF00264008

Lin, D.; Kakizono, T.; Nishio, N.; Nagai, S. Enhanced Cytochrome Formation and Stimulated Methanogenesis Rate by the Increased Ferrous Concentrations in Methanosarcina barkeri Culture. *FEMS Microbiol. Lett*. 1990, 68 (1–2), 89–92. DOI: 10.1111/j.1574-6968.1990.tb04128.x

5. EPR and Raman of Heme b in EPS

Answer：

Raman spectroscopy: Thank you for your insightful comment regarding the Raman. To ensure scientific rigor, we have removed the Raman of EPS. The Fe^{3+} state of the heme cofactor is instead supported by the UV-Vis and the heme-staining result in LDS-PAGE, which together provide independent and reliable evidence for the presence of electroactive heme b in the EPS.

EPR spectroscopy (The detection of Fe^{3+} rather than Fe^{2+}): Respectfully, it must be clarified that our manuscript neither claimed nor detected an Fe(II) EPR signal. As correctly pointed out, Fe(II) heme remains EPR-silent **(Ponomarenko et al., 2018; Walker, 1999)**. Found specifically at g = 3.04, the observed signal corresponds exclusively to low-spin ferric (Fe^{3+}) heme. During active metabolism, electron transfer to the methanogen is presumably facilitated by the heme b in the EPS operating in its

ferrous (Fe^{2+}) state. However, upon disconnection from the external power source, the lack of a continuous electron supply leaves the heme oxidized (Fe^{3+}). Aligning perfectly with our UV-Vis measurements, this explains the detection of Fe^{3+} rather than Fe^{2+}. To directly confirm the redox reversibility of this Fe^{3+} center, chemical reduction with sodium dithionite was performed, which completely abolished the g = 3.04 signal. This confirms that the signal originates exclusively from a reducible Fe^{3+} heme center.

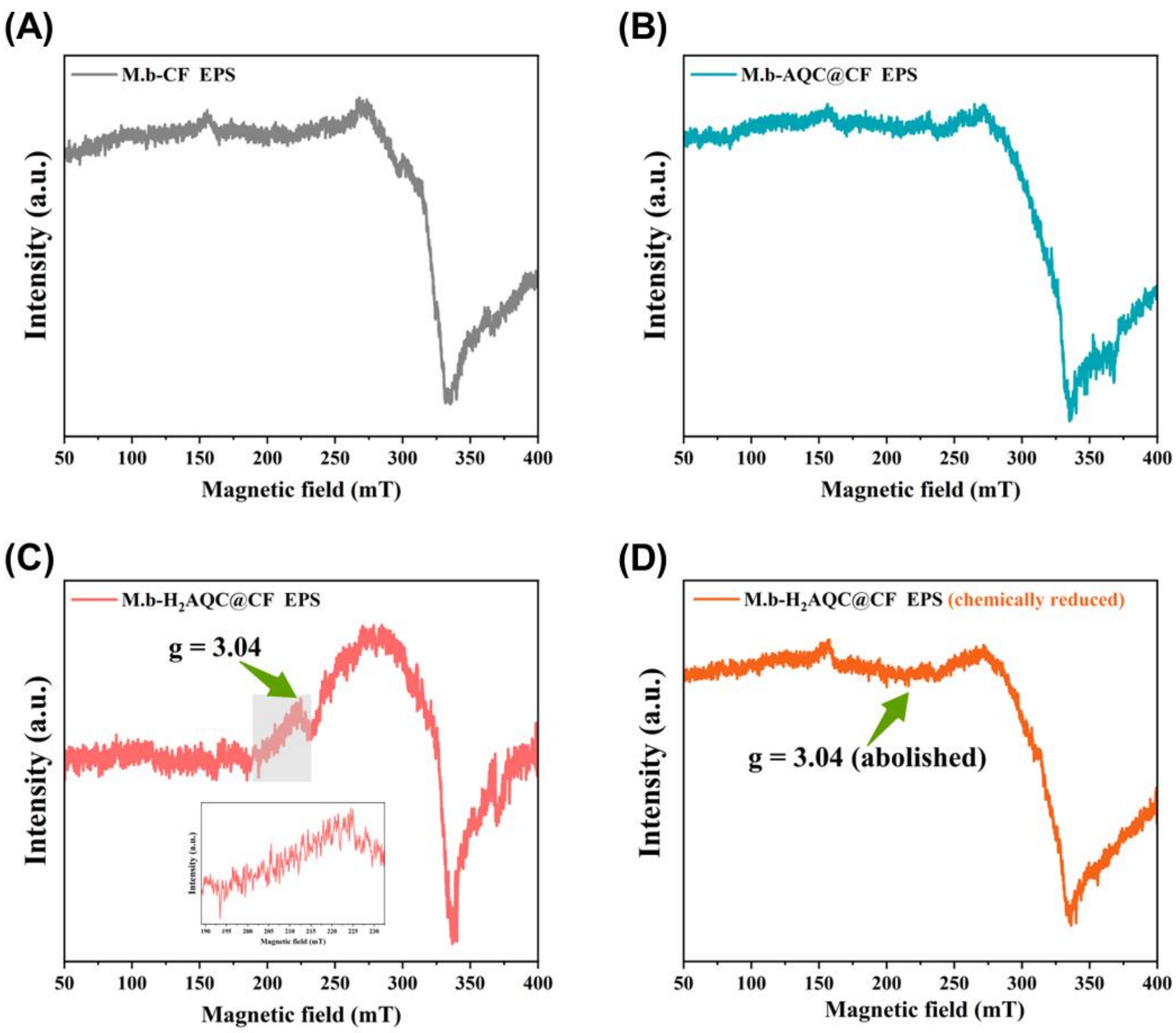

EPR spectrum of M.b-CF EPS (A), M.b-AQC@CF EPS (B), M.b-H_2AQC@CF EPS (C), M.b-H_2AQC@CF EPS after chemical reduction (D).

The following text has been added to the manuscript: The EPR spectrum displayed a discernible peak at g = 3.04 (Fig S9), characteristic of a low-spin ferric (Fe^{3+}) heme center[43,44]. Chemical reduction with sodium dithionite completely abolished this signal, confirming its origin from a reducible Fe^{3+} heme center.

Reference：

Ponomarenko, N.; Niklas, J.; Pokkuluri, P. R.; Poluektov, O.; Tiede, D. M. Electron Paramagnetic Resonance Characterization of the Triheme Cytochrome from Geobacter sulfurreducens. *Biochemistry* 2018, 57, 1722–1732. DOI: 10.1021/acs.biochem.7b00917

Walker, F. A. Magnetic Spectroscopic (EPR, ESEEM, Mössbauer, MCD and NMR)

Studies of Low-Spin Ferriheme Centers and Their Corresponding Heme Proteins. Coord. *Chem. Rev.* 1999, 185–186, 471–534. DOI: 10.1016/S0010-8545(99)00029-6

6. Application of transcriptomic Data

Answer：

We fully concur with your assessment that transcriptomic data cannot be used to infer protein abundance or extracellular localization. The revised manuscript clearly delineates the scope of these data.

The following text has been added to the manuscript: Of particular relevance, the HdrE subunit, which was specifically upregulated in the M. b-H_2AQC@CF biohybrid, harbors two heme b cofactors, representing a likely primary source of labile heme released into the EPS.

Reviewer #2's Comments

I served as Reviewer 2 in the previous review process. The conclusions of this study are well supported by the experimental data, and the revisions made in response to the earlier comments are reasonable. However, as I noted in my previous review, the proposed electron transfer mechanism between quinones and hemes was demonstrated using model compounds rather than cytochromes, which leaves open the question of whether such a mechanism actually occurs in the electron transfers in biotic – abiotic interfaces, let alone whether it functions within extracellular polymeric substances (EPS). While I do not question the validity of the conclusions of this study, I remain concerned that the work may lack the broader appeal required for publication in this journal.

Answer：

We sincerely thank you for the continued engagement, and for the constructive and focused concern raised. In this revision, we have directly addressed this concern by providing evidence from the real biological EPS rather than model systems alone. To validate the proposed quinone-heme interaction, UV-Vis spectroscopic experiments were conducted. Pure AQC (oxidized) and H_2AQC (reduced) exhibit absorption peaks at 339 nm and 407 nm, respectively. Mixing AQC with EPS (M.b-H_2AQC@CF) maintained the 339 nm peak, indicating that the EPS matrix does not inherently perturb AQC absorption. In contrast, introducing H_2AQC to EPS (M.b-H_2AQC@CF) triggered a redox reaction where H_2AQC was oxidized to AQC by Fe^{3+} heme b, resulting in an AQC peak at 334 nm. This 5 nm blue shift indicates that H_2AQC initially forms a π-π complex with heme b through electronic overlap, which allows the resulting AQC to remain "locked" in the interaction site and preserve the π-π stacked configuration. Critically, The observed 5 nm blue shift is consistent with H-type excitonic coupling arising from this face-to-face π-π interaction (DFT analysis), wherein antiparallel alignment of transition dipoles elevates the allowed excited-state energy **(Kasha.,1963; Hestand & Spano., 2018)**.

This conclusion is further corroborated by two mutually consistent lines of evidence: (i) broadening of the C=C stretching band in biomimetic Raman spectra, indicating

restricted molecular vibration upon π-π complex formation; (ii) the emergence of a C 1s XPS satellite peak at 290.30 eV, a recognized fingerprint of π-π interactions, which is absent in controls.

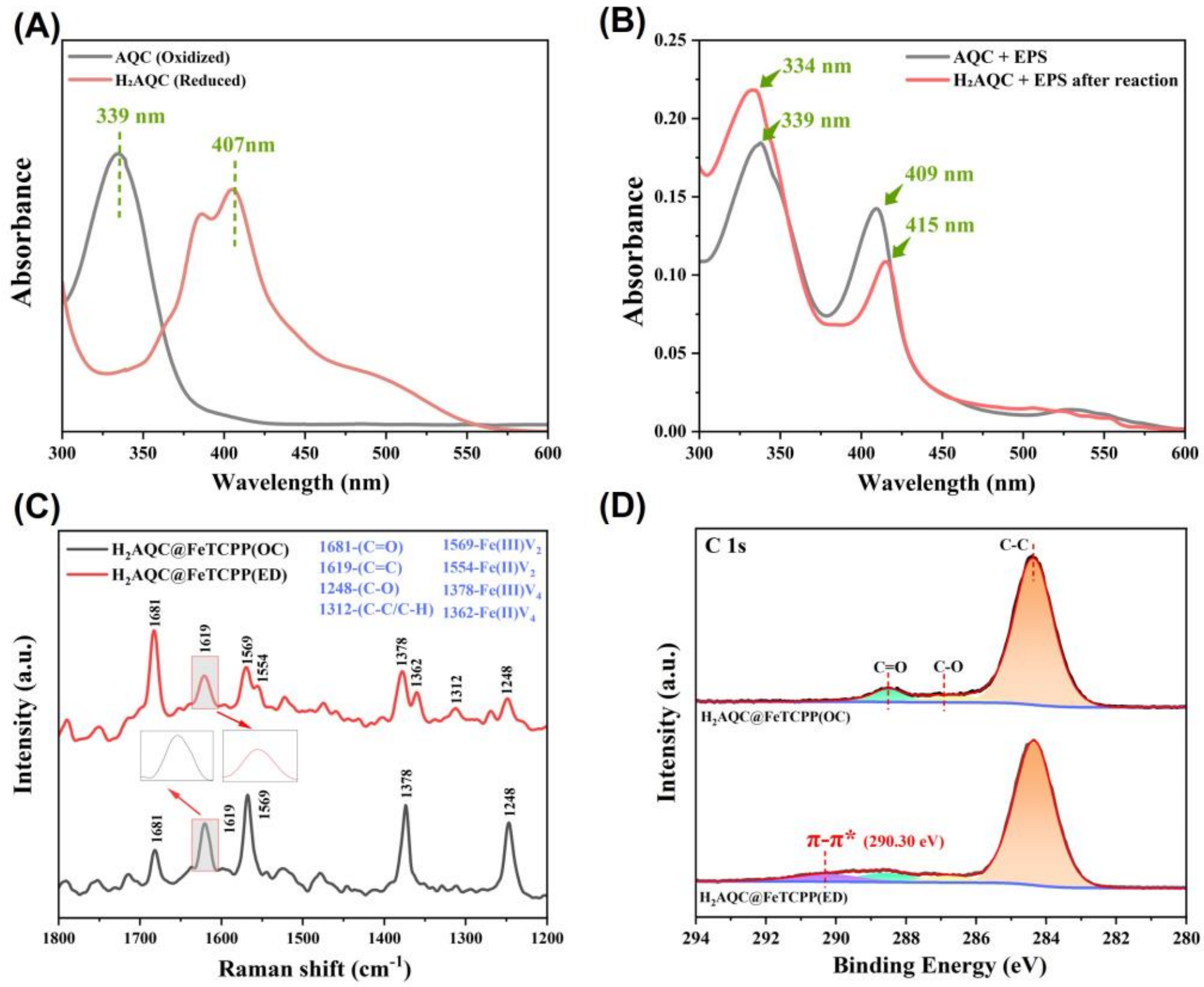

Spectroscopic evidence for quinone-heme π-π interactions. UV-Vis spectra of pure AQC (339 nm) and H_2AQC (407 nm) (A). UV-Vis spectra of AQC + EPS (M. b-H_2AQC@CF) and H_2AQC + EPS (M. b-H_2AQC@CF) after reaction (B). Raman (C) and XPS C1s spectra (D) of H_2AQC@FeTCPP under open-circuit (OC) and electrodeposition (ED)

The following text has been added to the manuscript: To validate the proposed quinone-heme interaction, UV-Vis experiments were conducted. Pure AQC (oxidized) and H_2AQC (reduced) exhibit absorption peaks at 339 nm and 407 nm, respectively (Fig S12). Mixing AQC with EPS (M. b-H_2AQC@CF) maintained the 339 nm peak, indicating that the EPS matrix does not inherently perturb AQC absorption. In contrast, introducing H_2AQC to EPS (M. b-H_2AQC@CF) triggered a redox reaction where H_2AQC was oxidized to AQC by Fe^{3+} heme b, resulting in an AQC peak at 334 nm (Fig 4C). This 5 nm blue shift indicates that H_2AQC initially forms a π-π complex with heme b through electronic overlap, which allows the resulting AQC to remain locked in the interaction site and preserve the π-π stacked configuration[49,50].

Reference:

Kasha, M. Energy transfer mechanisms and the molecular exciton model for molecular aggregates. *Radiat. Res.* 1963, 20, 55-70. DOI: 10.2307/3571331

Hestand, N. J.; Spano, F. C. Expanded Theory of H- and J-Molecular Aggregates: The Effects of Vibronic Coupling and Intermolecular Charge Transfer. *Chem. Rev.* 2018, 118 (15), 7069-7163. DOI: 10.1021/acs.chemrev.8b00244

Reviewer #3's Comments

Overall, the responses to my comments are largely accurate, and the revised manuscript is more internally consistent in both data interpretation and mechanistic framing. In particular, the mechanistic discussion of a heme-mediated relay at the bio–abiotic interface is more plausible than the original direct contact mechanism. I have two final suggestions.

- Please add a concise paragraph that states the engineering design principle derived from this work and how it can be generalized beyond the specific conditions tested here.
- Please expand your outlook to describe practical implications and potential applications in anaerobic digesters and/or other engineered environments, including key considerations for implementation and scalability.

Answer：

We sincerely thank you for your suggestions. Both points have been addressed by adding the following paragraph to the Discussion section:

The present findings establish a generalizable design principle: π-conjugated electrode interfaces can act as molecular traps for labile biological redox cofactors, converting aggregation-prone species into stable electrochemical relay units. This principle extends beyond the quinone-heme system to other aromatic cofactor-electrode pairings, offering a rational framework for next-generation bioelectrode design. In practical terms, this strategy is directly translatable to anaerobic digestion reactors and microbial electrosynthesis systems, where improving biotic-abiotic interfacial electron transfer remains a central challenge. Key considerations for scale-up include long-term stability of electrode functionalization, fabrication cost, and optimization of electrode geometry to maximize EPS-electrode contact.

Response letter to Reviewer

Dear Reviewer:

We greatly appreciated all the helpful comments from Reviewer on the manuscript NCOMMS-25-103702A (Entitled: Quinone-Heme π-π interactions bridge the biotic-abiotic interface for enhanced methanogenesis). According to these comments, we have revised the manuscript carefully as follows:

Black words represented the comments from Reviewer.

Blue words represented the responses from the Authors.

Red words represented the emphatic responses or revisions in the manuscript from the Authors.

Reviewer #1's Comments

1. Lack of distinction between heme-b vs. heme-c. The presented data suggested the presence of heme-c rather than heme-b. The beta band at 524 nm is more pronounced, which is a hallmark of heme-c due to the covalent thioether bonds restricting the heme. Similarly, the alpha band (556 nm) is well resolved as expected for heme-c, whereas heme-b shows a broader alpha band. Neither CV nor heme-staining shows that the observed data are specific to heme-b and not due to heme-b from intracellular cytochromes. The molecular weight of free heme is 616 Da, which is ~0.5 kDa. The authors observe signals just below 10 kDa, which is consistent with the signal of most periplasmic cytochrome c. The lack of signal unique to 0.5 kDa further indicates that there are no free heme-b in the EPS. The CV data further indicates heme-c due to the absence of distinct redox peaks expected for free hemes. Basic analysis of the first derivative needs to be performed for CV and EPR spectra, which need to be modeled for high-spin hemes, as free cyt-b should lack ligands and must be high spin. The lack of high-spin features further indicates the lack of cyt-b in the EPS.

Answer：

We sincerely thank you for your suggestions. It is acknowledged that the original

manuscript may not have articulated the concept of "labile heme b" with sufficient clarity, which could have led to the interpretation of this term as "fully free heme" and prompted the corresponding concerns. Each specific point is addressed in detail below, supported by additional experimental data and literature evidence.

1) Absence of heme-c in *M. barkeri*

The biosynthesis of heme-c requires a dedicated cytochrome c maturation (e.g. Ccm) system that catalyzes the formation of covalent thioether bonds between heme and protein (*Kranz et al., Microbiol. Mol. Biol. Rev., 2009*). Genomic analysis reveals that *M. barkeri* retains only a truncated residual sequence of the ccmE gene (192 bp), encoding no functional Ccm protein; furthermore, cell lysates of this strain have been directly confirmed to contain no cytochrome c via specific heme peroxidase staining assays (*Gupta et al., eLife, 2022*). These findings suggest that the heme detected in the EPS was heme-b.

2) UV-Vis supporting heme-b

UV-Vis measurement was performed using bovine heart cytochrome c (a pure heme-c standard), which yielded an α band at 550 nm and a β band at 520 nm, in full agreement with the literature standards (*Berry & Trumpower, Anal. Biochem., 1987*). The α band of the EPS sample is located at 556 nm, differing by 1 nm from the heme-b reference value (557 nm) and by 6 nm from the measured heme-c standard (550 nm) (*Barr & Guo, Bio Protoc., 2015*).

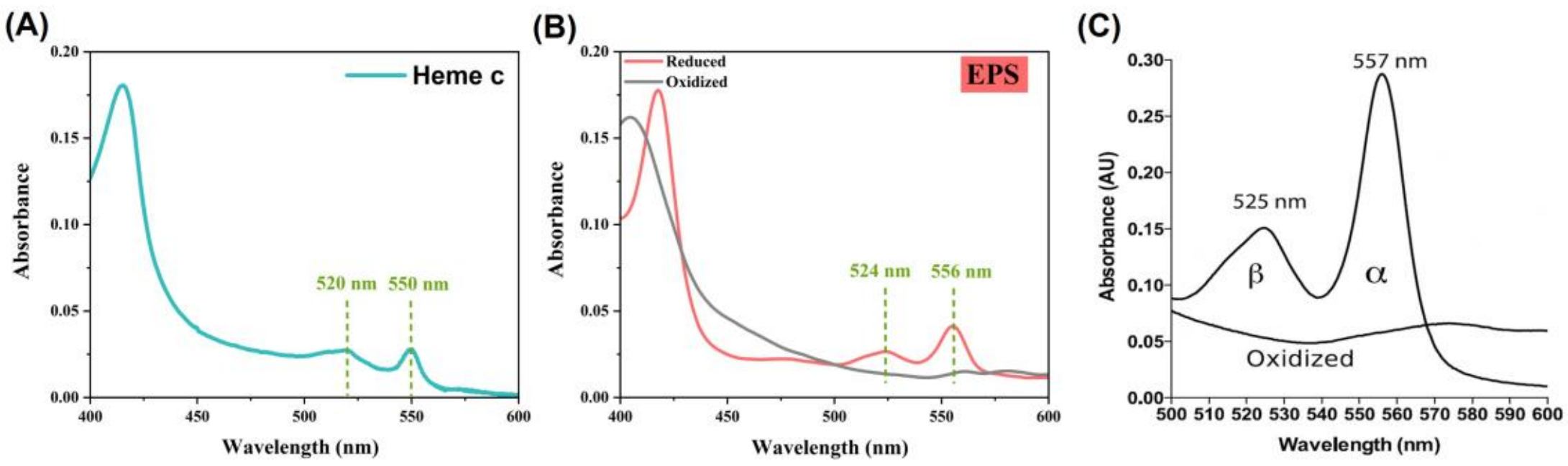

(A) Bovine heart cytochrome c with β and α bands at 520 nm and 550 nm, respectively; (B) EPS sample showing β and α bands at 524 nm and 556 nm, respectively; (C) Heme-b reference spectrum with β and α bands at 525 nm and 557 nm, respectively.

3) Interpretation of the LDS-PAGE

Clarification of a potentially ambiguous expression: the term used throughout this work is labile heme b, not fully free heme b (0.5 kDa). Following natural cell lysis, heme b-containing proteins (such as the HdrE subunit) undergo degradation in the EPS. While the <10 kDa band detected on the LDS-PAGE gel indicates that heme b is exposed, it is technically challenging to determine if the heme is completely free or still partially bound to degraded peptide residues. Because free heme also migrates below 10 kDa and runs at the dye front, it cannot be readily resolved from small heme-bearing fragments via standard heme staining (*San Francisco et al., Biochemistry, 2011*). Despite this limitation, the results indicate that heme b is indeed exposed. Given that intact heme b-containing proteins identified in *M. barkeri* (e.g., HdrE at ~23 kDa and VhoC at ~28 kDa) possess molecular weights above 10 kDa(*Heiden et al., Eur. J. Biochem., 1994; Deppenmeier et al., Eur. J. Biochem., 1995*). Therefore, this exposure provides support for the π-π interactions between quinone and heme proposed in our study.

4) First-derivative analysis of the CV

In accordance with your suggestion, first-derivative analysis was performed on the EPS CV (*Fricke et al., Energy Environ. Sci., 2008*). Owing to the substantial background capacitive current inherent to the EPS matrix, the derivative curve does not achieve ideal closure (*Marsili et al., Appl. Environ. Microbiol., 2008*). Nonetheless, the derivative analysis clearly reveals a redox feature centered at approximately -0.21 V (vs. Ag/AgCl), which is consistent with the reported redox potentials of heme b (*San Francisco et al., Biochemistry, 2011; Reedy & Gibney, Chem. Rev., 2004*).

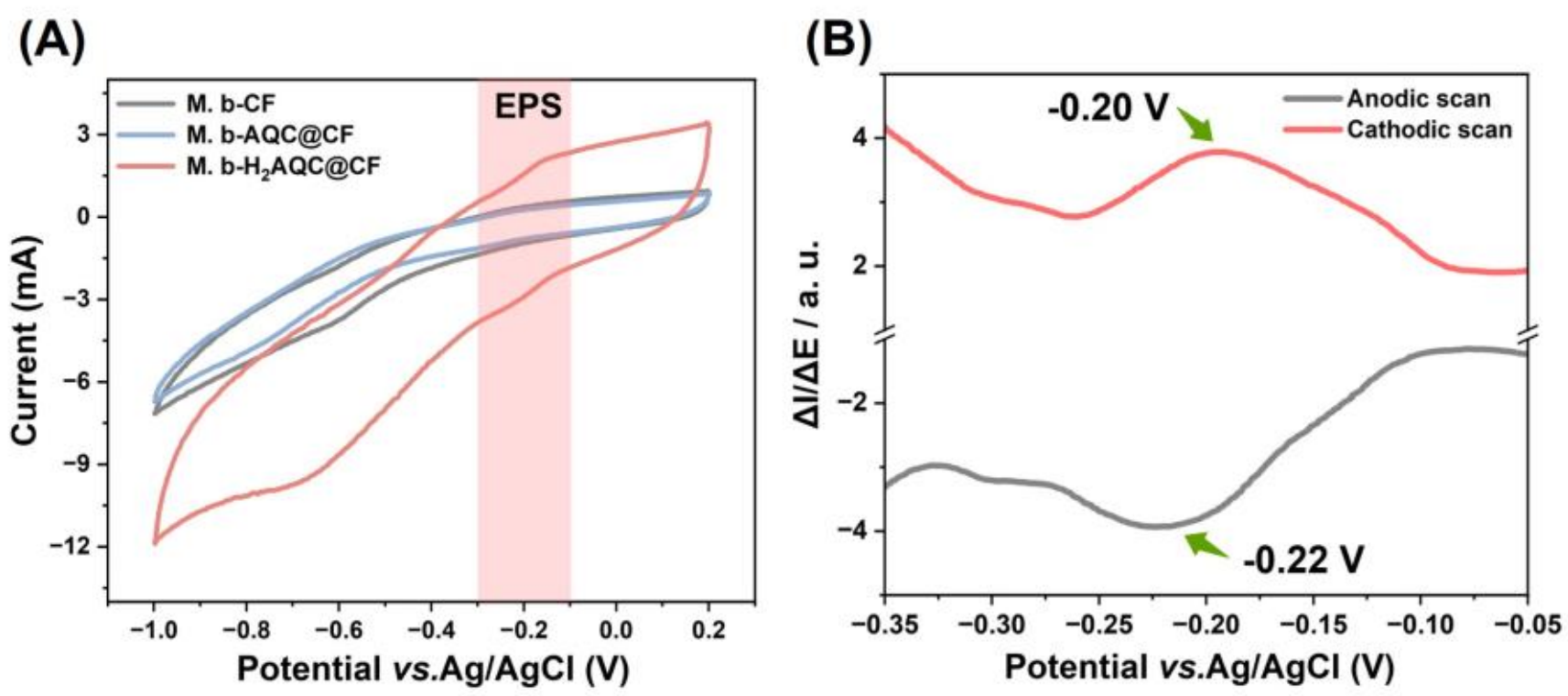

(A) CV curves of the three EPS groups; (B) First-derivative analysis of the CV curve for the M.b-

H_2AQC@CF group (red line).

5) Interpretation of the low-spin EPR signal

We note that CW-EPR spectra are inherently first-derivative lineshapes due to field modulation, so further derivative processing is not applicable. Free Fe(III) heme b normally shows a high-spin EPR signal near g ~ 6. The absence of this signal (at approximately 112 mT) in our EPR spectra due to thermodynamics. Free Fe(III) porphyrins are naturally unstable in watery biological environments. They spontaneously grab onto axial ligands to reach a stable six-coordinate low-spin state, which causes the g ~ 6 signal to disappear (*Walker, Coord. Chem. Rev., 1999*). Following natural cell lysis, the unstable heme b is likely stabilized in this low-spin state in two ways: by binding to external ligands in the EPS (such as imidazole, amines, or hydroxyl groups from extracellular proteins and polysaccharides); and partial retention of the original axial ligands from the parent b-type cytochrome, as supported by our LDS-PAGE results.

The following text has been added to the manuscript:

To distinguish heme b from heme c, bovine heart cytochrome c (a pure heme-c standard) was measured in parallel, yielding α/β bands at 550/520 nm (Fig S8), consistent with established literature values. There results providing spectral evidence that the observed heme is heme-b.

Notably, free Fe(III) heme b would typically exhibit a high-spin signal near g ~ 6, the absence of this feature is consistent with a six-coordinate low-spin state adopted by labile heme b in aqueous biological environments, where coordination with axial ligands (e.g., imidazole or amine groups from EPS-resident proteins and polysaccharides) or partial retention of original axial ligands from the parent cytochrome stabilizes this configuration.

Reference：

Kranz, R. G.; Richard-Fogal, C.; Taylor, J. S.; Frawley, E. R. Cytochrome c biogenesis: mechanisms for covalent modifications and trafficking of heme and for heme-iron redox control. Microbiol. Mol. Biol. Rev. 2009, 73 (3), 510-528.

Gupta, D.; Shalvarjian, K. E.; Nayak, D. D. An archaea-specific c-type cytochrome maturation machinery is crucial for methanogenesis in Methanosarcina acetivorans. eLife 2022, 11, e76970.

Berry, E. A.; Trumpower, B. L. Simultaneous determination of hemes a, b, and c from pyridine hemochrome spectra. Anal. Biochem. 1987, 161 (1), 1-15.

Barr, I.; Guo, F. Pyridine hemochromagen assay for determining the concentration of heme in purified protein solutions. Bio Protoc. 2015, 5 (18), e1594.

San Francisco, B.; Bretsnyder, E. C.; Rodgers, K. R.; Kranz, R. G. Heme Ligand Identification and Redox Properties of the Cytochrome c Synthetase, CcmF. Biochemistry 2011, 50, 10974-10985. DOI: 10.1021/bi201508t

Heiden, S., et al. (1994). Purification of a two-subunit cytochrome-b-containing heterodisulfide reductase from methanol-grown Methanosarcina barkeri. European Journal of Biochemistry, 221(1), 39-48.

Deppenmeier, U., et al. (1995). Analysis of the vhoGAC and vhtGAC Operons from Methanosarcina mazei Strain Gö1, Both Encoding a Membrane-bound Hydrogenase and a Cytochrome b. European Journal of Biochemistry, 227(1-2), 261-269.

Fricke, K.; Harnisch, F.; Schröder, U. On the use of cyclic voltammetry for the study of anodic electron transfer in microbial fuel cells. Energy Environ. Sci. 2008, 1 (1), 144-147.

Marsili, E.; Rollefson, J. B.; Baron, D. B.; Hozalski, R. M.; Bond, D. R. Microbial biofilm voltammetry: direct electrochemical characterization of catalytic electrode-attached biofilms. Appl. Environ. Microbiol. 2008, 74 (23), 7329-7337.

Reedy, C. J.; Gibney, B. R. Heme protein assemblies. Chem. Rev. 2004, 104 (2), 617-650.

Walker, F. A. Magnetic spectroscopic (EPR, ESEEM, Mössbauer, MCD and NMR) studies of low-spin ferriheme centers and their corresponding heme proteins. Coord. Chem. Rev. 1999, 185-186, 471-534.

2. Lack of evidence for heme relay. Heme-c cannot participate in electron relay because it is covered by protein and requires a near c-c distance (~ 3.5 A) for π-stacking that could enable efficient electron transfer. Even for heme-b, the porphyrin ring needs to be π-stacked (~ 3.5 A). The authors do not show that hemes are indeed spaced at such a stacking distance that can facilitate electron relay. X-ray diffraction of EPS should reveal any π-stacking present

Answer：

Thank you for your suggestions. The description in the previous manuscript was not precise enough about π-π interaction. Specifically, referencing Geobacter heme-stacked nanowires might cause the misunderstanding that a similar long-range continuous heme-stacked conductive structure is proposed in this work. This is not the case. It is clarified that the π-π interaction only refers to the interaction between quinone and heme, and has nothing to do with long-range heme-heme π-stacking. These references have now removed from the revised manuscript to avoid any such misunderstanding.

The XRD spectra showed typical amorphous broad peaks, consistent with the complex

and non-crystalline nature of the EPS samples. (*Singh et al., Carbohydr. Polym., 2011; Mathivanan et al., J. Polym. Environ., 2021*). This indicates that the heme in the EPS does not form long-range ordered π-stacked assemblies.

Although the EET ability of *M. barkeri* is widely confirmed, the mechanism supporting its long-range EET in the EPS is not yet fully clear. Current studies suggest that long-range EET in *M. barkeri* may rely on a more complex extracellular redox microenvironment (*Rotaru et al., Appl. Environ. Microbiol., 2014; Yee et al., Front. Energy Res., 2019*). Notably, recent studies by the Rotaru group reported that extracellular G-quadruplex RNA (G4-RNA) released on the *M. barkeri* cell surface may play an important role in the electron transfer between the cells and electrodes. Such conductive scaffolds require interaction with heme to facilitate electron transfer (*Kotoky et al., bioRxiv 2025; Jabaley et al., bioRxiv, 2026*). Therefore, the labile heme b in the EPS may contribute to EET by acting as potential redox sites for long-distance electron transfer in this work

It is agreed that clarifying the EET pathway in *M. barkeri* is an important direction. This will be further investigated as an independent project in our future work.

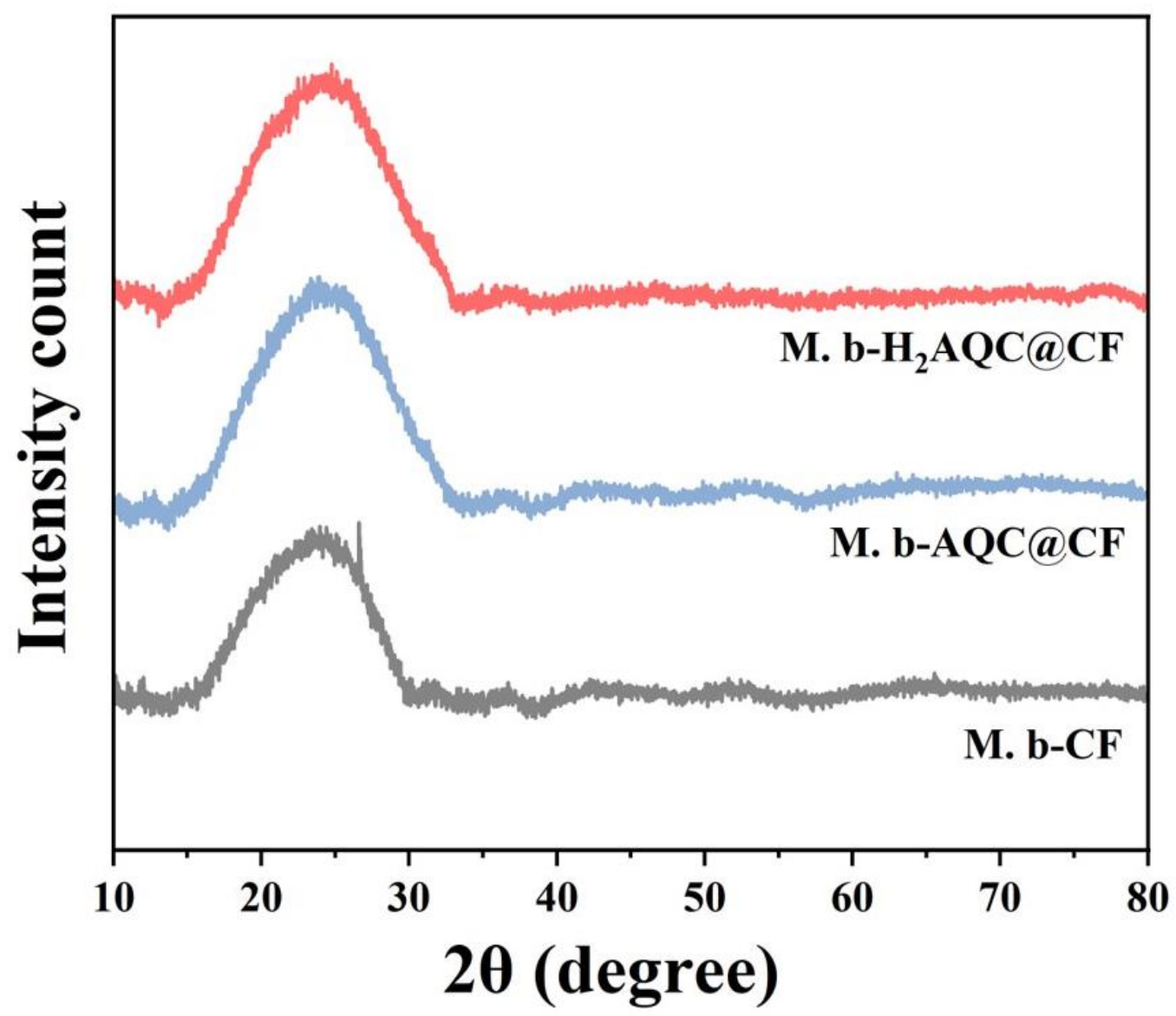

XRD characterization of the EPS of the three EPS

Reference：

Singh, R. P.; Shukla, M. K.; Mishra, A.; Kumari, P.; Reddy, C. R. K.; Jha, B. Isolation

and characterization of exopolysaccharides from seaweed associated bacteria Bacillus licheniformis. Carbohydr. Polym. 2011, 84, 1019-1026.

Mathivanan, K.; Chandirika, J. U.; Vinothkanna, A.; Govindarajan, R. K.; Meng, D.; Yin, H. Characterization and Biotechnological Functional Activities of Exopolysaccharides Produced by Lysinibacillus fusiformis KMNTT-10. J. Polym. Environ. 2021, 29, 1742-1751.

Rotaru, A.-E.; Shrestha, P. M.; Liu, F.; Markovaite, B.; Chen, S.; Nevin, K. P.; Lovley, D. R. Direct Interspecies Electron Transfer between Geobacter metallireducens and Methanosarcina barkeri. Appl. Environ. Microbiol. 2014, 80, 4599-4605.

Yee, M. O.; Snoeyenbos-West, O. L.; Thamdrup, B.; Ottosen, L. D. M.; Rotaru, A.-E. Extracellular Electron Uptake by Two Methanosarcina Species. Front. Energy Res. 2019, 7, 29.

Kotoky, R.; Ajunwa, O. M.; Kawaichi, S.; Meyer, R. L.; Rotaru, A.-E. Extracellular RNA drives Electromethanogenesis in a Methanogenic Archaeon. bioRxiv 2025. DOI: 10.1101/2025.07.06.663362.

Jabaley, A.; Frickman, T.; Jensen, P. B.; Justesen, M. L.; Chevrier, D. M.; Faivre, D.; Boesen, T.; Rotaru, A.-E. The metallome of Methanosarcina barkeri during electron uptake from a cathode. bioRxiv 2026. DOI: 10.64898/2026.02.14.705942.

3. Lack of physiological evidence of cells transferring electrons via EPS. The data presented suggest some electrochemical activity between the extracted EPS and the electrode, but that does not mean that cells are transferring electrons via EPS. Direct evidence of cellular electron transfer via EPS is needed through electrical measurements across the living cells using interdigitated electrodes and not from EPS to the electrode. See e.g. (Xu et al. JACS, 2018 140 (32), 10085-10089. DOI: 10.1021/jacs.8b05104)4)

Answer：

Thank you for the constructive feedback. It is fully acknowledged, as you pointed out, that the electrochemical activity between the extracted EPS and the electrode does not inherently equate to electron transfer by living cells via EPS. we fully agree with you viewpoint that completely ruling out all alternative mechanisms and definitively proving electron transport across living cells would require direct cross-cell conductance measurements, analogous to the approach demonstrated by Xu et al. Regrettably, executing this advanced characterization remains unfeasible at this stage due to our technical limitations. Nevertheless, your attention is respectfully directed to the in situ evidence presented in the manuscript, which provides support regarding this

issue.

First, in situ SECM show that electroactive hotspots matching the size of *M. barkeri* micro-colonies (~10-20 μm) appeared in the M. b-H_2AQC@CF group, whereas neither of the two control groups. When the applied potential was insufficient to reduce AQC (-0.3 V vs. Ag/AgCl), the hotspots disappeared. This demonstrates that the electroactivity does not originate from a homogeneous bulk "EPS-electrode" response, but rather is a localized electrochemical phenomenon occurring at the cellular locations.

Second, in situ CV revealed a significantly enhanced irreversible reduction response near -0.6 V (vs. Ag/AgCl) in the M. b-H_2AQC@CF group. This irreversibility indicates that the reduction products were continuously consumed by downstream metabolic processes. The absence of redox peaks in the M. b-CF control group confirms that direct cathode reduction of CO_2 to methane is negligible, and thus this sustained electron consumption can only be attributed to active CO_2 methanogenesis by *M. barkeri*. These results provide in situ electrochemical evidence that living cells transfer electrons from the electrode via the EPS layer to support cellular metabolism. Furthermore, the current density and methane production rate of this group at the same potential were significantly higher than those of the two control groups (nearly one order of magnitude). This difference indicates that electrode electrons, after passing through the EPS, are ultimately utilized for CO_2 reduction metabolism. This intact pathway is biologically functional, rather than merely an in vitro electrochemical response.

This study does not aim to thoroughly elucidate the entire EET pathway in *M. barkeri*. Given that *M. barkeri* lacks surface-exposed multiheme cytochromes, the mechanism of its EET remains an open question in the entire field. Instead, this work focuses on revealing the existence of π-π interactions between quinone and heme, and their correlation with the enhancement of interfacial electron transfer.

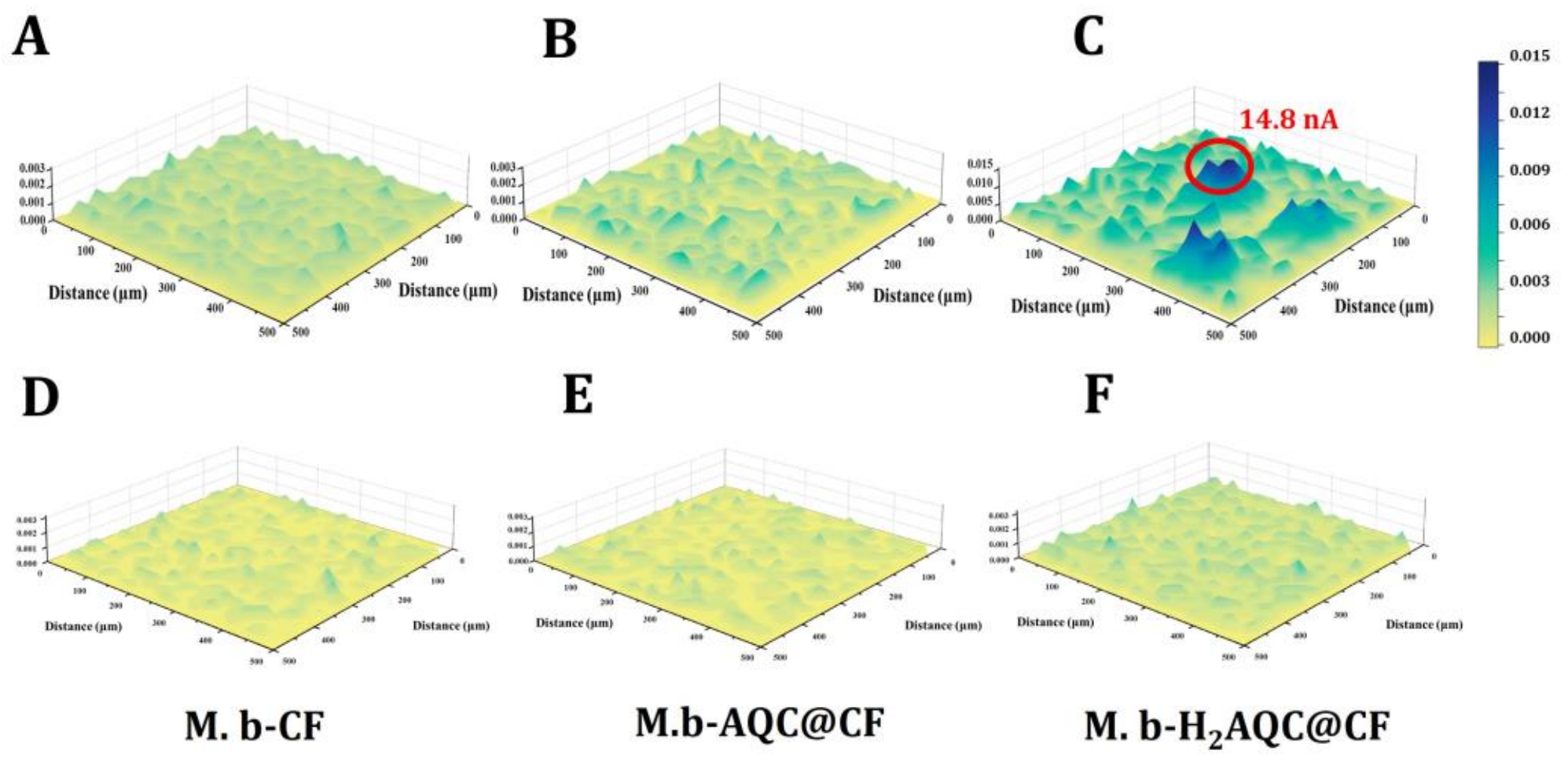

SECM maps of the corresponding groups at applied potentials of -0.8 V (A-C) and -0.3 V (D-F), revealing potential-dependent, localized electroactive micro-colonies.

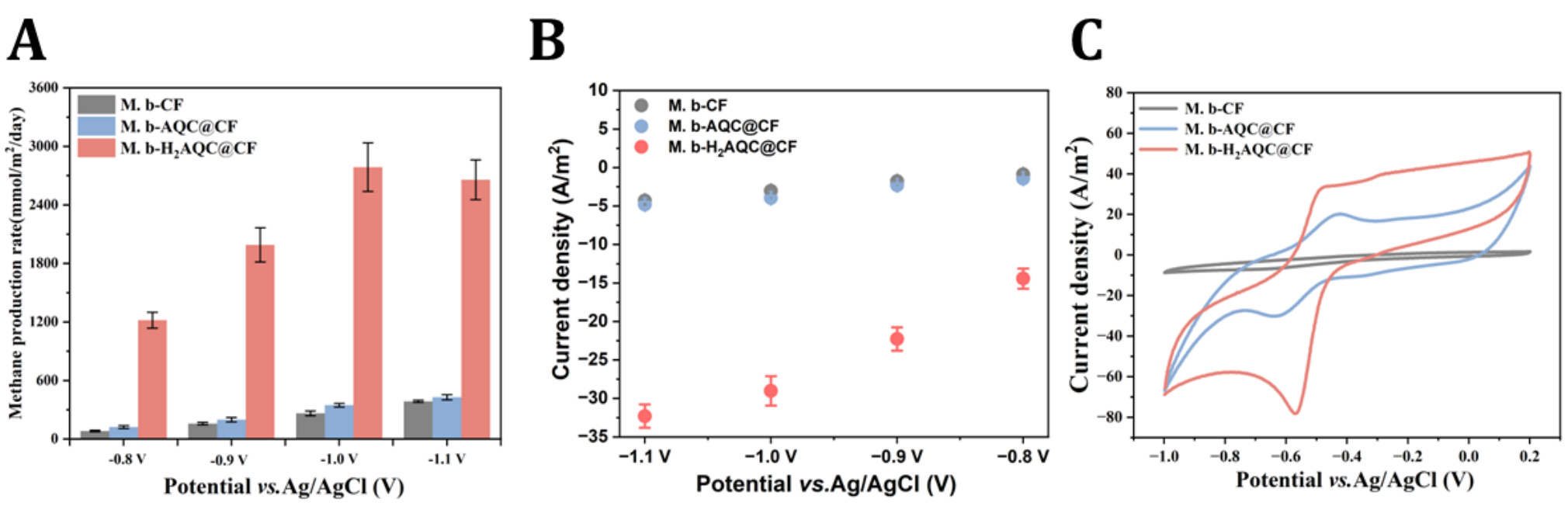

Methane production rate (A), current density (B) and in situ CV curves (C) for the three biohybrid systems

4. Physiological studies contradict the proposed model. A recent paper published in the same journal shows that Methanosarcina cannot exchange electrons in the absence of conductive particles externally added to cells (Nature Communications 17, 2708, 2026). This requirement for conductive particles contradicts the proposed model that cells can use EPS to transfer electrons.

Answer：

Thank you for raising this important point. Notably, the cited paper and recent studies demonstrating the intrinsic EET capacity of *M. barkeri* (independent of exogenous conductive particles) originate from the same research group (Rotaru group), illustrating that these EET phenotypes are highly context-dependent and non-mutually

exclusive. The cited paper investigated a mixed consortium serially transferred for a decade in the presence of GAC, which likely developed an obligate dependence on exogenous conductive particles as a result of long-term directed domestication. In contrast, our study was conducted under pure-culture conditions without such domestication. Preliminary findings from the same group suggest that M. barkeri pure cultures can utilize their native extracellular matrices to acquire electrons directly from electrodes (*Kotoky et al., bioRxiv, 2025; Jabaley et al., bioRxiv, 2026*), which conceptually aligns with our model. These observations collectively suggest that the two findings reflect distinct physiological contexts rather than contradictory conclusions.

Reference：

Kotoky, R.; Ajunwa, O. M.; Kawaichi, S.; Meyer, R. L.; Rotaru, A.-E. Extracellular RNA drives Electromethanogenesis in a Methanogenic Archaeon. bioRxiv 2025. DOI: 10.1101/2025.07.06.663362.

Jabaley, A.; Frickman, T.; Jensen, P. B.; Justesen, M. L.; Chevrier, D. M.; Faivre, D.; Boesen, T.; Rotaru, A.-E. The metallome of Methanosarcina barkeri during electron uptake from a cathode. bioRxiv 2026. DOI: 10.64898/2026.02.14.705942.

Response letter to Reviewer

Dear Reviewer:

We greatly appreciated all the helpful comments from Reviewer on the manuscript NCOMMS-25-103702B (Entitled: Quinone-Heme π-π interactions bridge the biotic-abiotic interface for enhanced methanogenesis). According to these comments, we have revised the manuscript carefully as follows:

Black words represented the comments from Reviewer.

Blue words represented the responses from the Authors.

Red words represented the emphatic responses or revisions in the manuscript from the Authors.

Reviewer #1's Comments

1. The role of HdrDE in the proposed mechanism requires clarification. The authors describe HdrDE as the source of heme (via lysis), the functional response to the established π-π channel (upregulation), and the proton pump driving ATP enhancement. However, the proposed mechanism is essentially a physicochemical event within the EPS and does not necessarily depend on HdrDE. The upregulation of HdrDE could simply reflect a feedback response to the increased downstream methanogenic flux rather than direct induction by the π-π interaction, and heme could equally originate from other cytochromes upon lysis. In addition, the proton-pumping function of HdrDE in Methanosarcina remains debated in the literature, and more cautious wording is recommended.

Answer：

Thank you for these valuable comments. We have carefully considered each point and revised the manuscript accordingly. Regarding the dual invocation of HdrDE as both a source of labile heme and a specific functional responder, we agree that this framing was internally inconsistent. The Discussion has been revised to present the HdrE subunit as one plausible candidate among several heme b-containing membrane

complexes, rather than positioning it as the primary source. Concerning the upregulation of HdrDE, we acknowledge that the transcriptomic data establish a correlation rather than a causal relationship with the π-π interaction. The observed upregulation may equally reflect an indirect feedback response to increased methanogenic flux, and the text has been revised accordingly to convey this ambiguity rather than implying direct induction by the π–π interaction. Finally, we appreciate the reminder regarding the debated proton-pumping function of HdrDE in *Methanosarcina*. The original causal language has been replaced with appropriately cautious wording to reflect the current state of uncertainty in the field.

The following text has been added to the manuscript:

Of particular relevance, the HdrE subunit harbors two heme b cofactors18, representing one plausible source of labile heme released into the EPS; other heme b-containing membrane complexes may contribute equally upon cell disruption.

Furthermore, HdrDE has been proposed to extrude intracellular protons to establish a transmembrane proton motive force during the mediation of extracellular electron transfer, thereby providing the driving force for ATP synthesis.

2. The contribution of H_2 evolution remains unaddressed, as H_2 production can occur below -0.8 V. Moreover, the reported faradaic efficiency of 85.8% applies only to the optimal condition; the fate of the remaining ~14% of electrons should be clarified.

Answer：

Thank you for your suggestions. The potential contribution of H_2-mediated indirect electron transfer (H_2-IET) has been carefully considered. At −1.0 V, H_2 production by M.b-H_2AQC@CF (118.9 mmol/m^2/day) was 2.5-fold higher than that by M.b-CF (47.7 mmol/m^2/day), consistent with its higher current density. However, methane production was enhanced by 10.6-fold (2787 vs. 263 mmol/m^2/day) over the same comparison. If H_2-IET were the dominant mechanism, methane enhancement should scale proportionally with H_2 enhancement; yet the observed methane enhancement far exceeds that of H_2. This discrepancy demonstrates that the majority of the enhanced methane production cannot be attributed to H_2-IET. Therefore, under the -1.0 V

potential, the remaining ~14% of electrons is attributed as a minor fraction to residual H_2, with the remainder likely allocated to biomass synthesis.

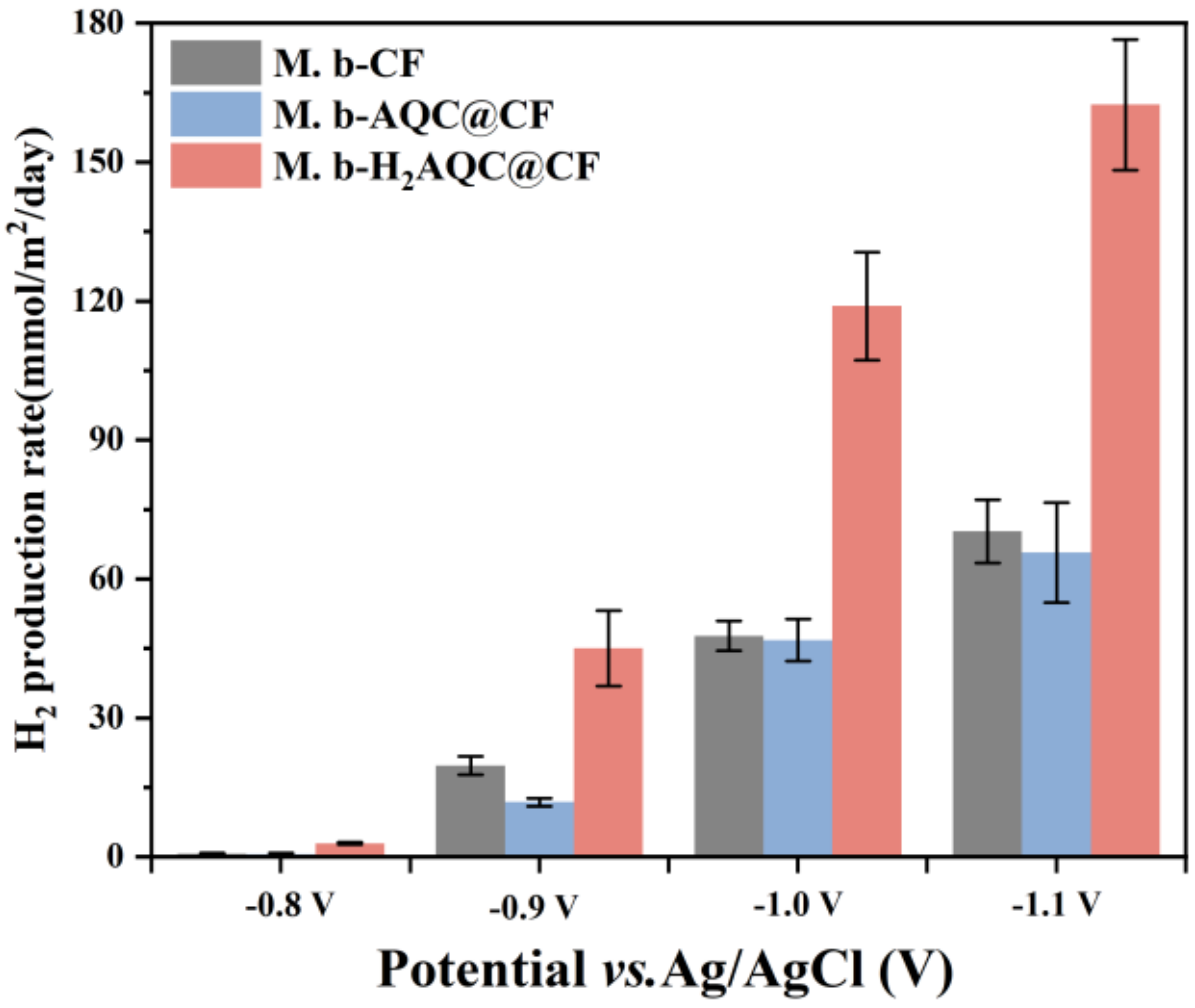

H_2 production rates of M. b-CF, M. b-AQC@CF, and M. b-H_2AQC@CF biohybrids.

3. In fig 3, both control groups exhibit weak and broad absorption features around 409 nm rather than a flat baseline, yet the main text describes them as "no such feature was observed in either control group". This should be clarified.

Answer:

Thank you for your suggestions. The original statement "no such feature was observed in either control group" was imprecise. As your correctly notes, weak and broad absorption features are detectable around 409 nm in the control groups, likely reflecting trace levels of heme b present at concentrations insufficient for reliable spectroscopic resolution. In contrast, the sharp and well-defined Soret band at 409 nm in M. b-H_2AQC@CF reflects a substantially higher local concentration of labile heme b stabilized by π-π interactions with H_2AQC.

The following text has been added to the manuscript:

UV-Vis spectroscopy revealed a sharp Soret band at 409 nm exclusively in the EPS of the M. b-H_2AQC@CF group, consistent with monomeric ferric (Fe^{3+}) heme, while the control groups exhibited only weak and broad absorption in this region, possibly attributable to trace levels of heme b below the threshold for reliable spectroscopic

resolution.

4. The identification of heme b relies solely on comparison with a heme c standard (cytochrome c), with α/β band differences of only 4-6 nm. A heme b standard should be included as a positive control to validate the assignment.

Answer：

Thank you for raising this important point. The heme b assignment is supported by comparison with established literature reference values. The α band of the EPS sample (556 nm) differs by only 1 nm from the literature heme b reference (557 nm; Barr & Guo, Bio Protoc., 2015), compared to a 6 nm difference from the directly measured heme c standard (550 nm) (*Berry & Trumpower, Anal. Biochem., 1987*). This, combined with corroborating EPR and LDS-PAGE, supports the heme b assignment.

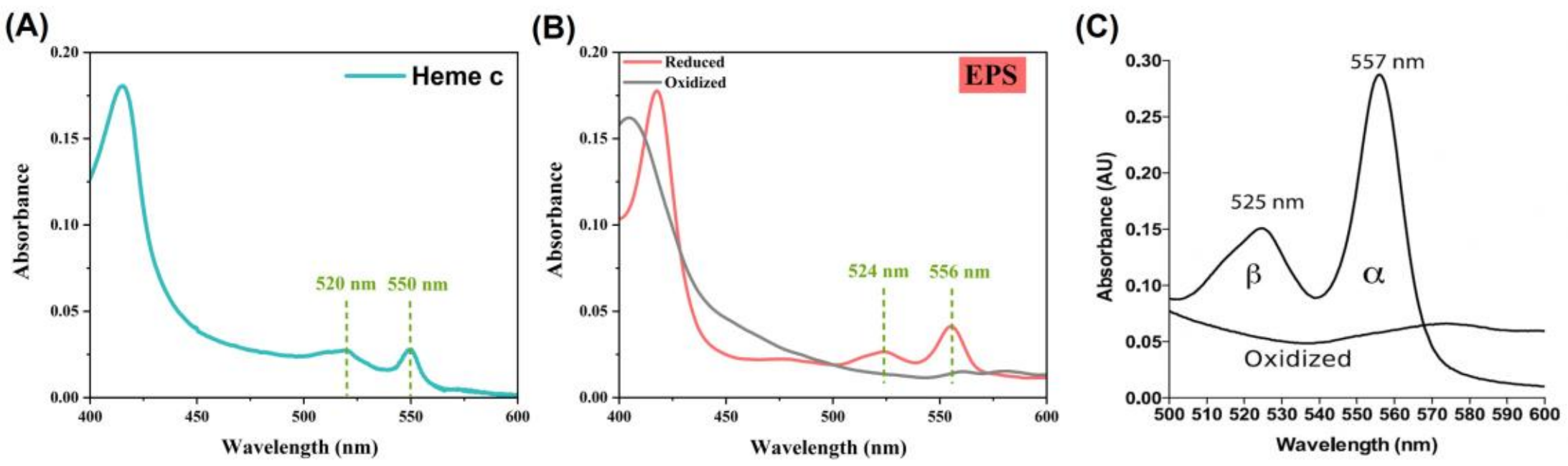

(A) Bovine heart cytochrome c with β and α bands at 520 nm and 550 nm, respectively; (B) EPS sample showing β and α bands at 524 nm and 556 nm, respectively; (C) Heme-b reference spectrum with β and α bands at 525 nm and 557 nm, respectively.

Reference：

Berry, E. A.; Trumpower, B. L. Simultaneous determination of hemes a, b, and c from pyridine hemochrome spectra. Anal. Biochem. 1987, 161 (1), 1-15.

5. Several methodological details require clarification: (1) The "fresh medium" is not specified, including whether it still contains cysteine, methanol, or other potential electron donors. (2) The EIS methodology is absent from the SI, and the equivalent circuit should be justified. (3) The normalization basis for the ATP and qPCR data is not specified (per mg protein? per cm² electrode area?).

Answer：

Thank you for your suggestions.

(1) The fresh medium used during electrochemical testing was prepared without cysteine, methanol, or any other exogenous organic electron donors, ensuring that CO_2 served as the sole carbon and electron acceptor source throughout the measurements. The materials and methods section has been revised to explicitly state this: Prior to performance evaluation for electrocatalytic CO_2 reduction to methane, the electrolyte in both chambers was replaced with fresh, sterile medium free of exogenous organic electron donors, and the cathode headspace was subsequently flushed with 99.999% pure CO_2 to serve as the sole carbon source.

(2) The equivalent circuit Rohm(RctW/CPE), shown in Figure 2B, was selected to reflect the physical processes at the biotic-abiotic interface. Rohm represents the ohmic resistance of the electrolyte; Rct captures the interfacial charge transfer resistance and serves as the primary parameter for comparing EET efficiency across groups; the constant phase element (CPE) replaces an ideal capacitor to account for the surface heterogeneity of the biofilm; and the Warburg element (W) describes diffusion-limited mass transport at the electrode-biofilm interface. The quality of fit for all three groups is confirmed by the close agreement between experimental data and fitted curves shown in the insets of Figure 2B.

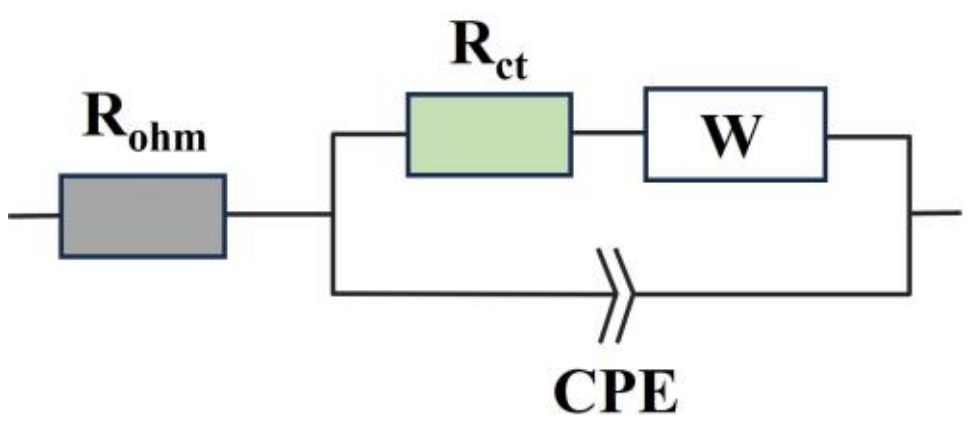

The EIS methodology and equivalent circuit justification have been added to the Supporting Information as follows: EIS measurements were performed in a standard three-electrode system using a redox probe electrolyte containing 5 mM $K_3[Fe(CN)_6]/K_4[Fe(CN)_6]$ in 1 M KCl. The frequency range was set from 0.01 Hz to 1.0×10^5 Hz with a sinusoidal AC perturbation amplitude of 10 mV. Data fitting was performed using ZsimpWin software.

(3) Biomass was quantified by qPCR of the mcrA gene, with copy numbers normalized to the projected electrode area (copies cm^{-2}), as described in the Materials and Methods. For visualization in Figure 1C, the area-normalized mcrA copy numbers were further expressed as relative biomass (normalized to the M. b-CF group, set as 1) to facilitate direct comparison of the fold-differences among the three biohybrid groups. The y-axis of Figure 1C has been revised from "a.u." to "Relative biomass" accordingly. ATP content was normalized to total protein extracted from the biofilm samples and expressed as nmol mg^{-1} protein. The revised values for M. b-CF, M. b-AQC@CF, and M. b-H_2AQC@CF are 2.23, 2.72, and 8.27 nmol mg^{-1} protein, respectively, and Figure 1D has been updated accordingly.

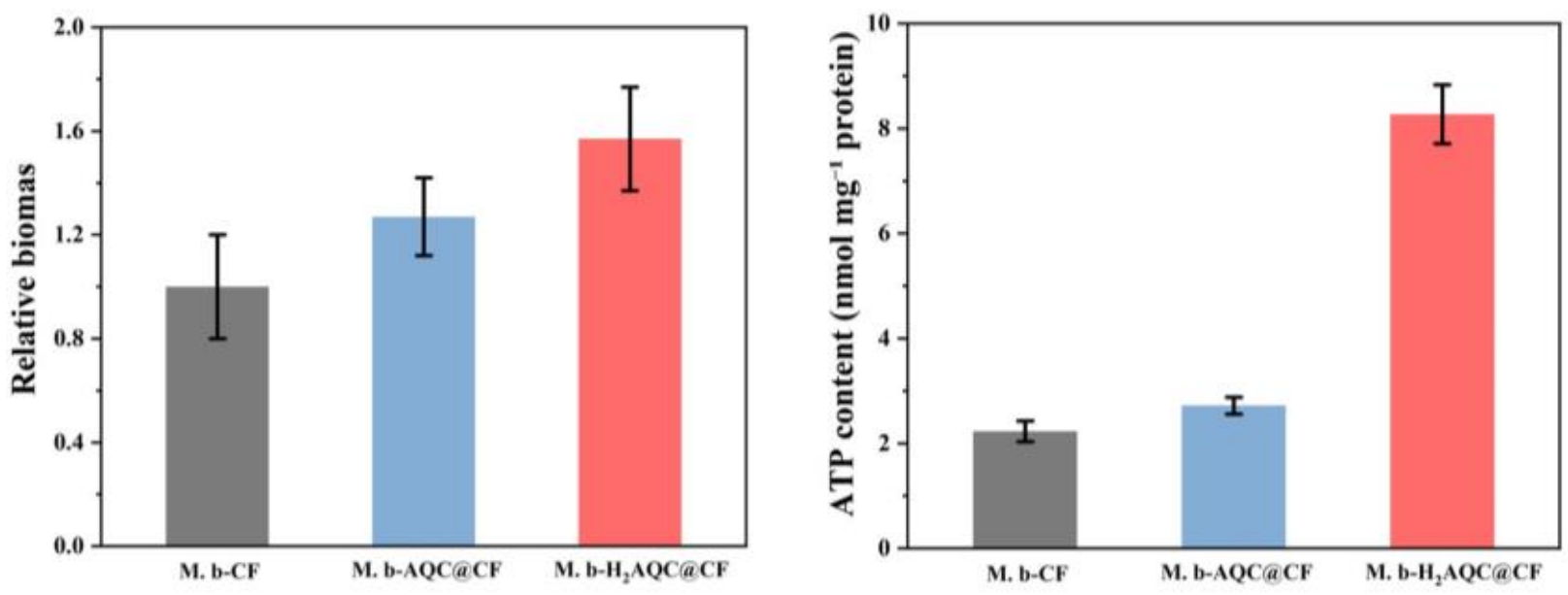

Relative biomass quantification based on total DNA concentration; ATP activity of *M. barkeri* biofilms
